# Molecular Evolution of Vertebrate Neurotrophins: Co-Option of the Highly Conserved Nerve Growth Factor Gene into the Advanced Snake Venom Arsenalf

**DOI:** 10.1371/journal.pone.0081827

**Published:** 2013-11-29

**Authors:** Kartik Sunagar, Bryan Grieg Fry, Timothy N. W. Jackson, Nicholas R. Casewell, Eivind A. B. Undheim, Nicolas Vidal, Syed A. Ali, Glenn F. King, Karthikeyan Vasudevan, Vitor Vasconcelos, Agostinho Antunes

**Affiliations:** 1 CIMAR/CIIMAR, Centro Interdisciplinar de Investigação Marinha e Ambiental, Universidade do Porto, Porto, Portugal; 2 Departamento de Biologia, Faculdade de Ciências, Universidade do Porto, Porto, Portugal; 3 Venom Evolution Lab, School of Biological Sciences, The University of Queensland, St. Lucia, Queensland, Australia; 4 Institute for Molecular Bioscience, University of Queenland, St Lucia, Queensland, Australia; 5 Molecular Ecology and Evolution Group, School of Biological Sciences, Bangor University, Bangor, United Kingdom; 6 Alistair Reid Venom Research Unit, Liverpool School of Tropical Medicine, Liverpool, United Kingdom; 7 Département Systématique et Evolution, Service de Systématique Moléculaire, UMR 7138, Muséum National d’Histoire Naturelle, Paris, France; 8 HEJ Research Institute of Chemistry, International Center for Chemical and Biological Sciences (ICCBS), University of Karachi, Karachi, Pakistan; 9 Wildlife Institute of India, Dehradun, Uttarakhand, India; National Institute of Allergy and Infectious Diseases, United States of America

## Abstract

Neurotrophins are a diverse class of structurally related proteins, essential for neuronal development, survival, plasticity and regeneration. They are characterized by major family members, such as the nerve growth factors (NGF), brain-derived neurotrophic factors (BDNF) and neurotrophin-3 (NT-3), which have been demonstrated here to lack coding sequence variations and follow the regime of negative selection, highlighting their extremely important conserved role in vertebrate homeostasis. However, in stark contrast, venom NGF secreted as part of the chemical arsenal of the venomous advanced snake family Elapidae (and to a lesser extent Viperidae) have characteristics consistent with the typical accelerated molecular evolution of venom components. This includes a rapid rate of diversification under the significant influence of positive-selection, with the majority of positively-selected sites found in the secreted β-polypeptide chain (74%) and on the molecular surface of the protein (92%), while the core structural and functional residues remain highly constrained. Such focal mutagenesis generates active residues on the toxin molecular surface, which are capable of interacting with novel biological targets in prey to induce a myriad of pharmacological effects. We propose that caenophidian NGFs could participate in prey-envenoming by causing a massive release of chemical mediators from mast cells to mount inflammatory reactions and increase vascular permeability, thereby aiding the spread of other toxins and/or by acting as proapoptotic factors. Despite their presence in reptilian venom having been known for over 60 years, this is the first evidence that venom-secreted NGF follows the molecular evolutionary pattern of other venom components, and thus likely participates in prey-envenomation.

## Introduction

Venom, a complex biochemical cocktail of biologically active components, such as proteins, peptides, amino acids, neurotransmitters and polyamines, has underpinned the diversification and evolutionary success of several animal lineages [1]. This key evolutionary innovation is employed by a plethora of animals for predation, competitor deterrence and defence [2–5]. The scientific consensus is that venom components originate via toxin recruitment events, as part of which physiological protein-encoding genes are duplicated and the new copies are selectively expressed in the venom gland [5–15]. Over the years, our understanding of the origin and diversification of snake venoms has greatly increased, largely due to advances in transcriptomics and proteomics [16–22]. However, the precise role of certain proteins, which are secreted as part of the biochemical venom arsenal, still remains to be elucidated. Nerve growth factor (NGF), a key member of the neurotrophin family, is one such class of protein whose presence in snake venoms has been intriguing. Since its discovery in the late 1950s , NGF has been reported from the venoms of various caenophidian (advanced) snakes, including members of the front-fanged elapid and viperid families as well as from venomous lizards [23–29], but its function and relative importance in snake venoms remains unknown [8].

Neurotrophins represent a family of structurally related proteins, crucial for neuronal development, survival, death, regeneration and plasticity. According to the classical neurotrophic hypothesis, neurotrophins are produced in limiting amounts and the survival of the innervating neurons is dependent on winning the competition for sufficient quantities of these factors [30,31]. Neurotrophins contain gene family members such as nerve growth factor (NGF), brain-derived neurotrophic factor (BDNF), neurotrophin-3 (NT-3) and neurotrophin-4/5 (NT-4/5) [32–35], all of which function by interacting with the p75 neurotrophin receptor (NTR) in their proneurotrophin forms and various structurally related tropomyosin-related kinase or Tyrosine kinase receptors (Trk) in their active cleaved form [36,37]. While NGF specifically activates TrkA [38], BDNF and NT-4/5 interact with TrkB [36,39]. NT-3 primarily interacts with TrkC and is unique in also being capable of weakly binding to both TrkA and TrkB [36].

 Neurotrophins have been extensively studied not only because they are perceived as one of the primary factors responsible for the complexity of vertebrate nervous systems, but also because of their involvement in cognition and memory. Knockout of the genes encoding NGF, BDNF and NT-3 genes is fatal in mice, highlighting the importance of these proteins for survival and normal neuronal development. Not surprisingly, abnormalities associated with the production of neurotrophins have been linked with neuropathies and neurodegenerative disorders.

 In order to investigate the role of NGF in the venom of Toxicofera reptiles [24], we have investigated the molecular evolution of these proteins in reptilian (turtles; squamates: Laterata, Scinciformata, Gekkota; Toxicofera lizards: Anguimorpha and Iguania; Henophidia snakes; advanced snakes: Elapidae, Viperidae and ‘non-front-fanged’ advanced snakes) and mammalian lineages, by employing sophisticated protein and codon-level selection assessments. We further compare the molecular evolution of NGF with the other major members of the neurotrophin family, namely BDNF and NT-3, in a wide array of reptilian and mammalian lineages. Molecular evolution analyses conducted on a dataset of 1183 nucleotide sequences revealed that these genes have remained largely unchanged since their origin over 300 million years (the split between mammals and reptiles: www.timetree.org) due to the extremely important functions they play in vertebrate homeostasis.

## Results

**Figure 1:**
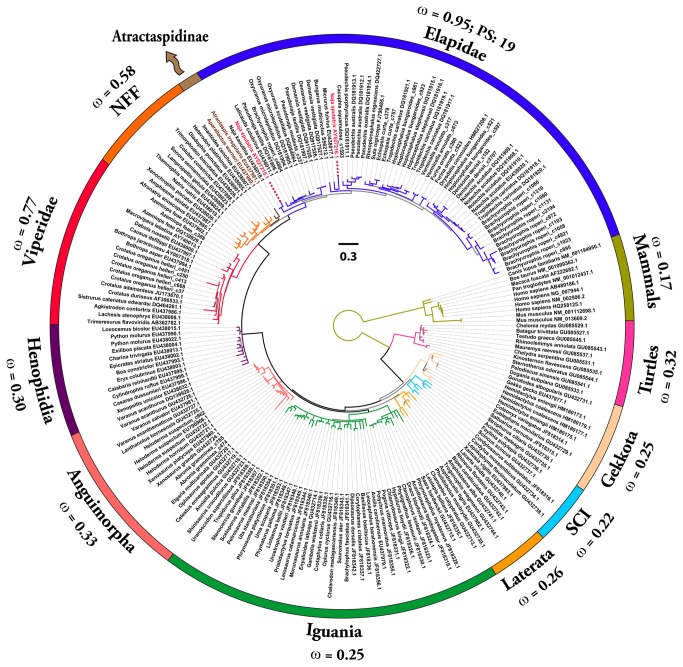
Bayesian molecular phylogeny of nerve growth factors (NGF). Branches with the Bayesian posterior probability (B.P.P) of less than 0.85 are highlighted in grey (remaining in colours). Site model 8 (M8) computed ω values for respective lineages are presented. The number of positively selected sites (PP ≥ 0.95) detected by M8’s Bayes-Empirical Bayes (BEB) approach in Elapidae lineage is also indicated. Elapid sequences representing putative duplicate genes are indicated with red labels [NFF: “non-front-fanged” advanced snakes; SCI: Scinciformata].

**Figure 2:**
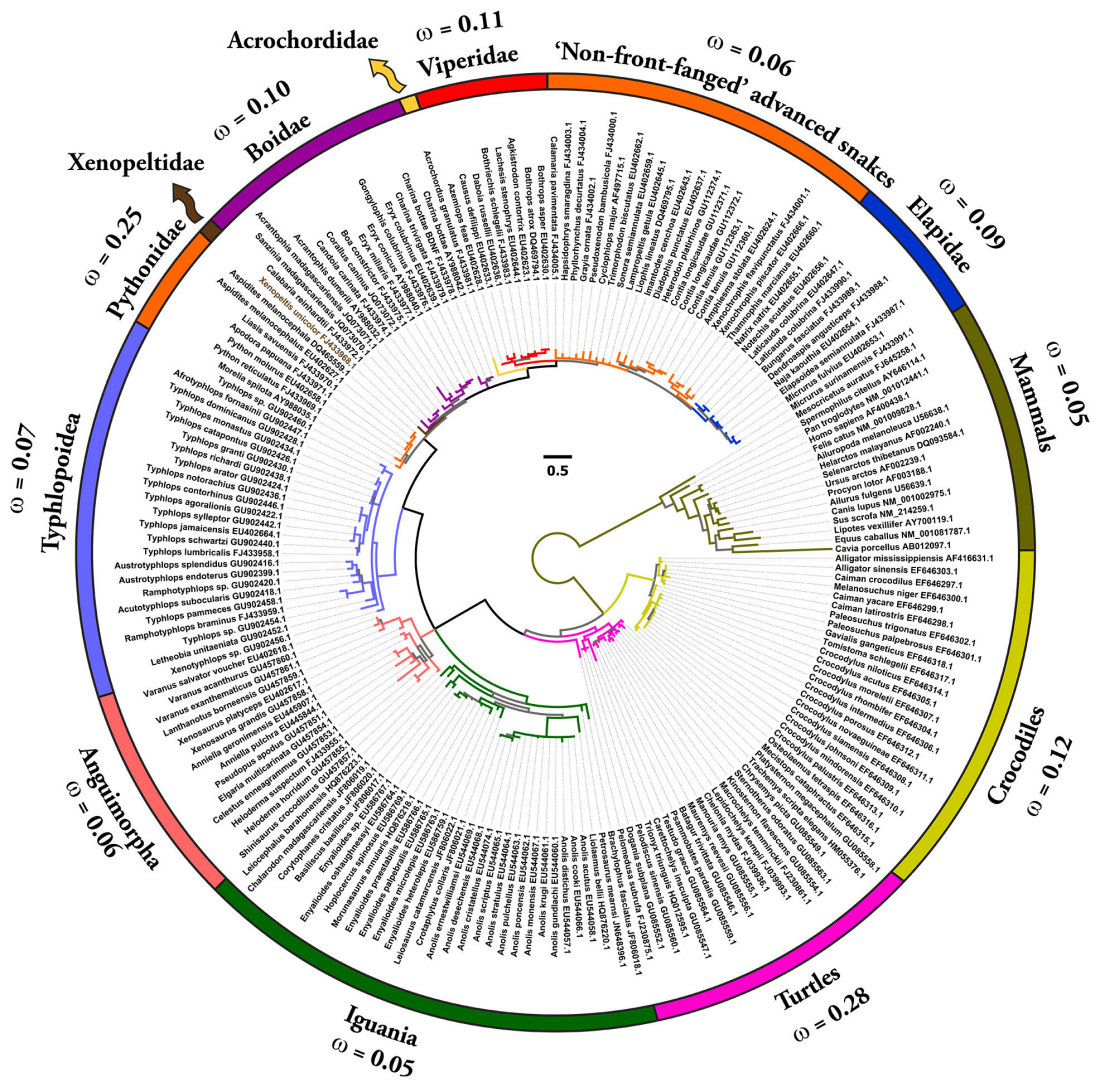
Bayesian molecular phylogeny of brain-derived neurotrophic factors (BDNF). Branches with the Bayesian posterior probability (B.P.P) of less than 0.85 are highlighted in grey (remaining in colours). Site model 8 (M8) computed ω values for respective lineages are presented.

**Figure 3:**
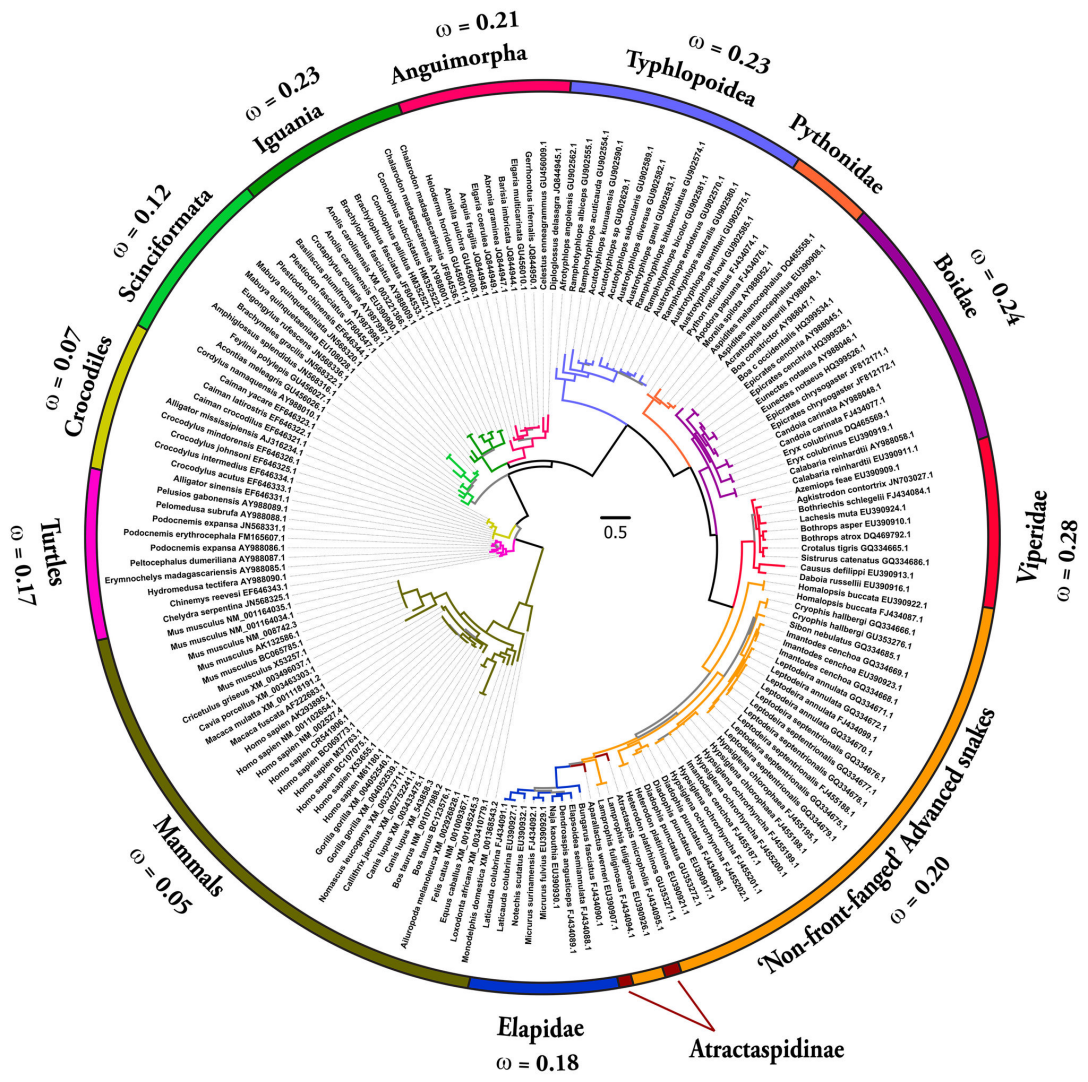
Bayesian molecular phylogeny of neurotrophin-3 (NT-3). Branches with the Bayesian posterior probability (B.P.P) of less than 0.85 are highlighted in grey (remaining in colours). Site model 8 (M8) computed ω values for respective lineages are presented.

Bayesian and maximum-likelihood analyses of NGF, BDNF and NT-3 genes retrieved trees with the same topology (Figures 1-3; Figure S1-S4), which were in concordance with the earlier reported phylogenies of neurotrophins [40].

One ratio model (ORM), the simplest of the lineage-specific models, computed ω of less than 0.50, therefore indicating an influence of negative selection on NGF, BDNF and NT-3 genes in all of the mammalian and reptilian lineages examined (Tables S1-S3). This highly conservative model can only detect positive selection when the ω ratio averaged over all the sites along the lineages in a phylogenetic tree is significantly greater than one. Nevertheless, the computed ω of 0.86 for the Elapidae NGF highlights a relatively greater accumulation of variation in this lineage (Table S1).

**Figure 4:**
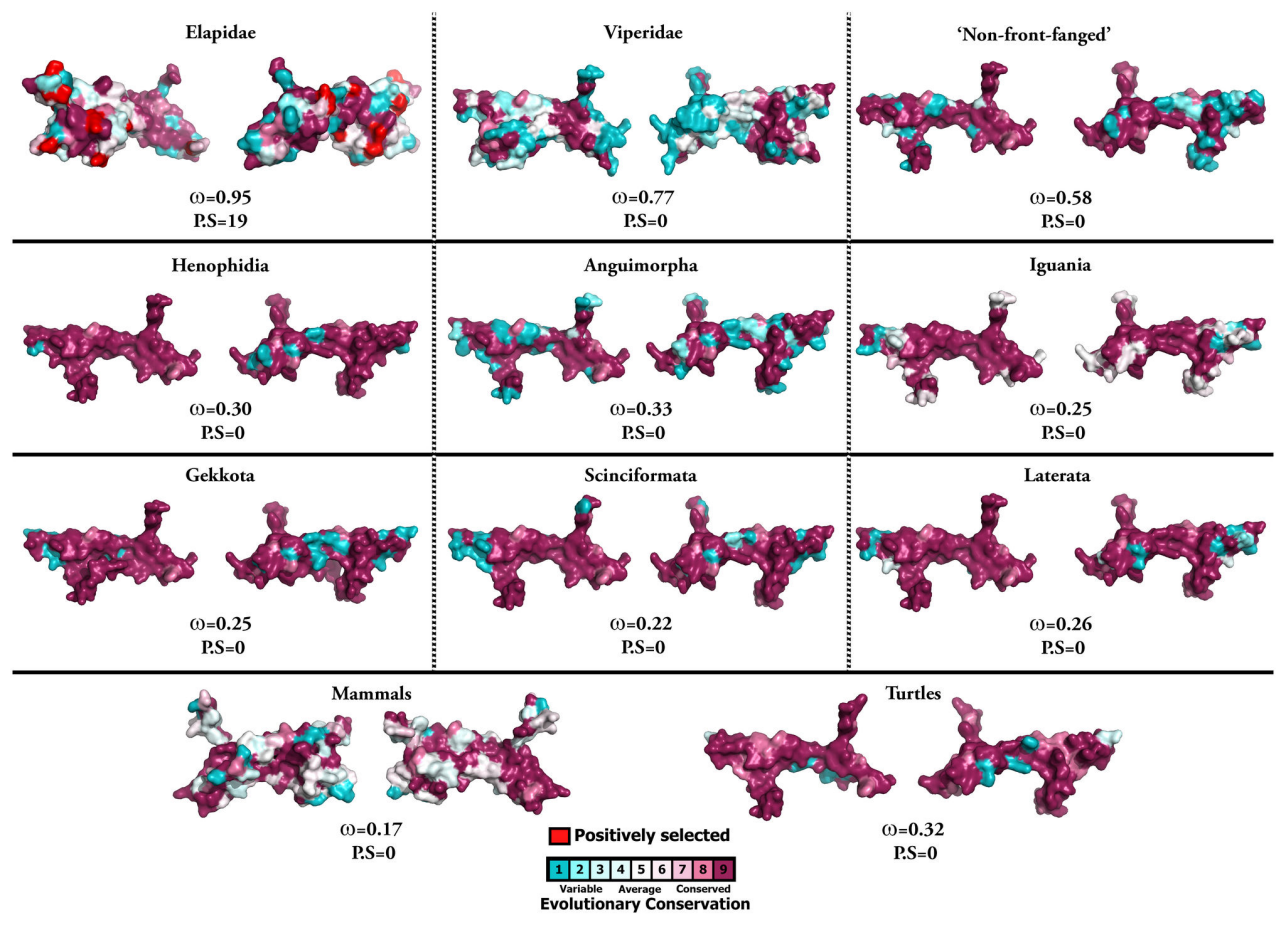
Molecular evolution of nerve growth factors. Three-dimensional homology models of nerve growth factors, depicting the locations of positively selected sites (in red). The ω values (Model 8) for the respective taxa along with the number of positively selected sites (PP ≥ 0.95, Bayes-Empirical Bayes approach) are indicated.

**Figure 5:**
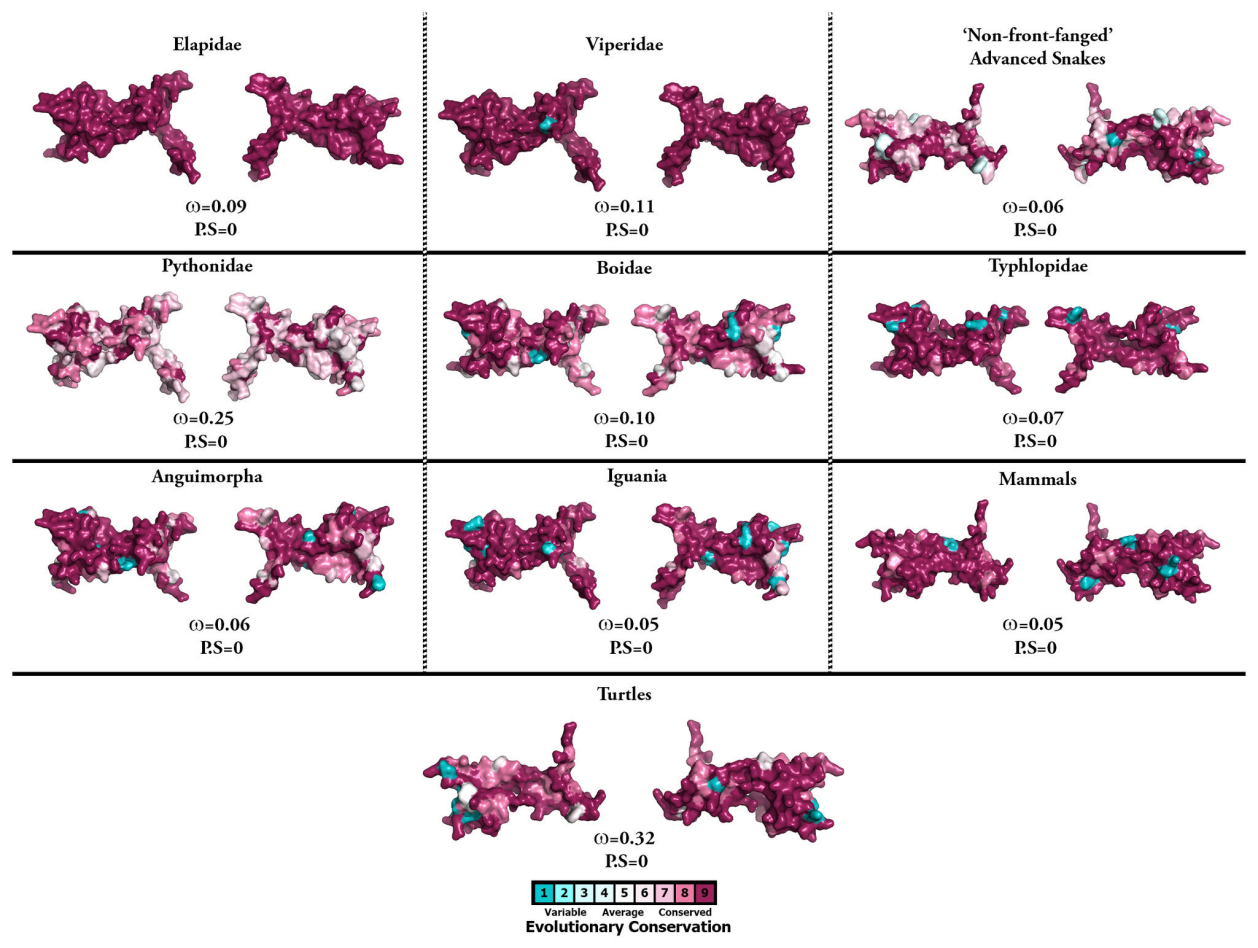
Molecular evolution of brain-derived neurotrophic factors. Three-dimensional homology models of brain-derived neurotrophic factors (BDNF), depicting the molecular evolution of these non-venomous homologues. The ω values (Model 8) for the respective taxa (PP ≥ 0.95, Bayes-Empirical Bayes approach) are indicated.

To detect episodic diversifying selection, which only affects certain sites in the protein, we employed site-specific models (Tables 1-3; Tables S1-S3). Like ORM, site model 8 (M8) also indicated a strong influence of negative selection on various reptilian and mammalian NGF, BDNF and NT-3 genes (Tables 1-3; Tables S1-S3; Figures 4 and 5). However, Elapidae NGF was found to be rapidly evolving (ω = 0.95). The Bayes Empirical Bayes (BEB) approach in M8 identified as many as 19 positively-selected sites (13% of the total sites with ω of 4.09) in Elapidae NGF, indicating the strong influence of positive selection on this lineage (Table 1 and Table S1). Although, M8 failed to detect variation in the Viperidae (ω = 0.77) and ‘non-front-fanged’ advanced snake (ω = 0.58) NGF lineages, as many as 9% (ω = 2.40) and 8% (ω = 2.50) of the total codon sites were detected as rapidly diversifying in the respective lineages. Single Likelihood Ancestral Counting (SLAC), Fixed Effects Likelihood (FEL), Random Effects Likelihood (REL), Mixed Effects Model of Evolution (MEME), Fast, Unconstrained Bayesian AppRoximation (FUBAR) and integrative approach, conclusively supported these findings and highlighted the complete lack of variations in NGF, BDNF and NT-3 genes in various reptilian and mammalian lineages examined (Tables 1-3). However, elapid venom NGF was found to be rapidly evolving under the influence of positive selection (integrative analyses: 34 positively selected sites). Viperidae NGF was also found to accumulate relatively greater variations (integrative analyses: 8 positively selected sites) in comparison to the non-venomous reptilian and mammalian NGF lineages. Analysis of NGF from the venom-glands of ‘non-front-fanged’ advanced snakes and venomous lizards (Anguimorpha and Iguania) was hampered by the scarcity of sequences. Despite this, the integrative approach was able to identify five positively selected sites in the ‘non-front-fanged’ advanced snake lineages.

**Table 1:** Molecular evolution of Nerve Growth Factor (NGF).

		**SLAC^a^**	**FEL^b^**	**REL^c^**	**FUBAR^d^**	**Integrative**	**MEME^e^**	**M8**	**M2a**	**Clade**
**Elapidae**										
	ω>**1^f^**	2	13	28	22	34		19	15	
	ω<**1^g^**	12	20	11	18	22	21	(14+5)	(13+2)	2.38
	ω**=**	0.90	-	1.93	-	-		0.95	0.94	
**Viperidae**										
	ω>**1^f^**	0	0	2	0	8		0	0	
	ω<**1^g^**	3	13	0	7	13	6	-	-	1.03
	ω**=**	0.65	-	0.88	-	-		0.77	0.77	
**NFF**										
	ω>**1^f^**	0	1	3	2	5		0	0	
	ω<**1^g^**	1	2	0	3	3	2	-	-	0.57
	ω**=**	0.59	-	0.67	-	-		0.58	0.58	
**Henophidia**										
	ω>**1^f^**	0	0	0	0	1		0	0	
	ω<**1^g^**	3	12	1	13	13	1	-	-	0.0001
	ω**=**	0.30	-	0.43	-	-		0.30	0.30	
**Iguania**										
	ω>**1^f^**	0	0	0	0	4		0	0	
	ω<**1^g^**	35	55	73	83	87	4	-	-	0.20
	ω**=**	0.26	-	0.76	-	-		0.25	0.29	
**Anguimorpha**										
	ω>**1^f^**	0	0	0	0	1		0	0	
	ω<**1^g^**	4	13	0	15	15	1	-	-	0.20
	ω**=**	0.33	-	0.48	-	-		0.33	0.36	
**Gekkota**										
	ω>**1^f^**	0	0	3	1	5		0	0	
	ω<**1^g^**	1	15	0	16	16	1	-	-	-
	ω**=**	0.24	-	0.34	-	-		0.25	0.25	
**Scinciformata**										
	ω>**1^f^**	0	0	0	0	1		0	0	
	ω<**1^g^**	8	18	All	18	19	1	-	-	-
	ω**=**	0.22	-	0.26	-	-		0.22	0.25	
**Laterata**										
	ω>**1^f^**	0	0	0	0	0		0	0	
	ω<**1^g^**	5	12	0	14	15	0	-	-	-
	ω**=**	0.26	-	0.36	-	-		0.26	0.27	
**Turtles**										
	ω>**1^f^**	0	0	0	1	1		0	0	
	ω<**1^g^**	9	13	0	14	14	0	-	-	-
	ω**=**	0.33	-	0.38	-	-		0.32	0.34	
**Mammals**										
	ω>**1^f^**	0	1	0	0	4		0	0	
	ω<**1^g^**	24	54	All	86	86	3	-	-	-
	ω**=**	0.21	-	0.22	-	-		0.17	0.11	

**a**: Single Likelihood Ancestor Counting

**b**: Fixed-effects likelihood

**c**: Random-effects likelihood

**d**: Fast, Unconstrained Bayesian AppRoximation

**Integrative**: Sites detected in common by SLAC, FEL, REL, FUBAR and MEME

**e**: Sites detected as experiencing episodic diversifying selection (0.05 significance) by the Mixed Effects Model Evolution (MEME)**M8**: Positively-selected sites detected using the Bayes Empirical Bayes approach implemented in M8. Sites detected at 0.99 and 0.95 significance are indicated in the parenthesis**M2a**: Positively-selected sites detected using the Bayes Empirical Bayes approach implemented in M2a. Sites detected at 0.99 and 0.95 significance are indicated in the parenthesis**Clade**: Omega computed by the clade model

**f**: Number of positively selected sites at 0.05 significance (for SLAC, FEL) or 50 Bayes factor (for REL) / number of sites under pervasive diversifying selection at the posterior probability ≥0.9 (FUBAR)

**g**: Number of negatively selected sites at 0.05 significance (for SLAC, FEL) or 50 Bayes factor (for REL) / number of sites under pervasive purifying selection at the posterior probability ≥0.9 (FUBAR)

**ω**: mean dN/dS**NFF**: “non-front-fanged” advanced snakes

**Table 2:** Molecular evolution of Brain-derived Neurotrophic Factors (BDNF).

		**SLAC^a^**	**FEL^b^**	**REL^c^**	**FUBAR^d^**	**Integrative**	**MEME^e^**	**M8**	**M2a**
**Elapidae**									
	ω>**1^f^**	0	0	0	0	0		0	0
	ω<**1^g^**	1	3	0	3	3	0	-	-
	ω**=**	0.09	-	0.25	-	-		0.09	0.09
**Viperidae**									
	ω>**1^f^**	0	0	0	0	0		0	0
	ω<**1^g^**	1	3	13	4	13	0	-	-
	ω**=**	0.10	-	-	-	-		0.11	0.11
**NFF**									
	ω>**1^f^**	0	0	0	0	0		0	0
	ω<**1^g^**	5	19	14	22	22	0	-	-
	ω**=**	0.08	-	0.35	-	-		0.06	0.06
**Typhlopoidea**									
	ω>**1^f^**	0	0	0	0	0		0	0
	ω<**1^g^**	27	47	0	93	93	0	-	-
	ω**=**	0.07	-	0.11	-	-		0.07	0.07
**Boidae**									
	ω>**1^f^**	0	0	1	1	1		0	0
	ω<**1^g^**	3	10	3	10	10	0	-	-
	ω**=**	0.0	-	0.28	-	-		0.09	0.09
**Pythonidae**									
	ω>**1^f^**	0	0	0	1	1		0	0
	ω<**1^g^**	0	2	11	7	11	0	-	-
	ω**=**	0.21	-	0.23	-	-		0.15	0.15
**Iguania**									
	ω>**1^f^**	0	0	0	0	0		0	0
	ω<**1^g^**	24	56	111	99	24	1	-	-
	ω**=**	0.05	-	-	-	0.05		0.05	0.05
**Anguimorpha**									
	ω>**1^f^**	0	0	2	0	0		0	0
	ω<**1^g^**	9	22	9	23	9	0	-	-
	ω**=**	0.08	-	0.15	-	0.08		0.06	0.06
**Crocodiles**									
	ω>**1^f^**	0	0	0	0	0		0	0
	ω<**1^g^**	0	8	20	1	0	0	-	-
	ω**=**	0.09	-	-	-	0.09		0.12	0.12
**Turtles**									
	ω>**1^f^**	0	0	4	0	5		0	0
	ω<**1^g^**	6	18	2	18	18	1	-	-
	ω**=**	0.10	-	0.14	-	-		0.05	0.06
**Mammals**									
	ω>**1^f^**	0	0	2	0	4		0	0
	ω<**1^g^**	48	72	51	113	72	2	-	-
	ω**=**	0.05	-		-	-		0.05	0.06

**a**: Single Likelihood Ancestor Counting

**b**: Fixed-effects likelihood

**c**: Random-effects likelihood

**d**: Fast, Unconstrained Bayesian AppRoximation

**Integrative**: Sites detected in common by SLAC, FEL, REL, FUBAR and MEME

**e**: Sites detected as experiencing episodic diversifying selection (0.05 significance) by the Mixed Effects Model Evolution (MEME)

**M8**: Positively-selected sites detected using the Bayes Empirical Bayes approach implemented in M8. Sites detected at 0.99 and 0.95 significance are indicated in the parenthesis

**M2a**: Positively-selected sites detected using the Bayes Empirical Bayes approach implemented in M2a. Sites detected at 0.99 and 0.95 significance are indicated in the parenthesis

**f**: Number of positively selected sites at 0.05 significance (for SLAC, FEL) or 50 Bayes factor (for REL) / number of sites under pervasive diversifying selection at the posterior probability ≥0.9 (FUBAR)

**g**: Number of negatively selected sites at 0.05 significance (for SLAC, FEL) or 50 Bayes factor (for REL) / number of sites under pervasive purifying selection at the posterior probability ≥0.9 (FUBAR)

**ω**: mean dN/dS**NFF**: “non-front-fanged” advanced snakes

**Table 3:** Molecular evolution of of Neurotrophin 3 (NT-3).

		**SLAC^a^**	**FEL^b^**	**REL^c^**	**FUBAR^d^**	**Integrative**	**MEME^e^**	**M8**	**M2a**
**Elapidae**									
	ω>**1^f^**	0	0	0	0	0		0	0
	ω<**1^g^**	1	4	0	4	4	0	-	-
	ω**=**	0.19	-	0.21	-	-		0.18	0.19
**Viperidae**									
	ω>**1^f^**	0	0	0	0	0		0	0
	ω<**1^g^**	0	5	0	3	5	0	-	-
	ω**=**	0.30	-	0.49	-	-		0.28	0.30
**NFF**									
	ω>**1^f^**	0	2	5	1	8		0	0
	ω<**1^g^**	43	61	79	82	95	2	-	-
	ω**=**	0.22	-	0.35	-	-		0.20	0.27
**Typhlopoidea**									
	ω>**1^f^**	1	2	8	2	10		0	0
	ω<**1^g^**	48	69	49	106	106	2	-	-
	ω**=**	0.24	-	0.49	-	-		0.23	0.26
**Boidae**									
	ω>**1^f^**	0	0	0	1	3		0	0
	ω<**1^g^**	7	23	10	26	26	2	-	-
	ω**=**	0.24	-	0.51	-	-		0.24	0.28
**Scinciformata**									
	ω>**1^f^**	0	0	0	0	0		0	0
	ω<**1^g^**	7	27	75	30	75	0	-	-
	ω**=**	0.13	-	-	-	-		0.12	0.13
**Iguania**									
	ω>**1^f^**	0	2	4	3	7		0	0
	ω<**1^g^**	27	41	23	58	58	3	-	-
	ω**=**	0.25	-	0.43	-	-		0.23	0.26
**Anguimorpha**									
	ω>**1^f^**	0	0	0	0	1		0	0
	ω<**1^g^**	7	20	All	23	23	1	-	-
	ω**=**	0.24	-	0.32	-	-		0.21	0.24
**Crocodiles**									
	ω>**1^f^**	0	0	0	0	0		0	0
	ω<**1^g^**	0	1	all	1	1	0	-	-
	ω**=**	0.08	-	0.08	-	-		0.07	0.07
**Turtles**									
	ω>**1^f^**	0	0	0	0	0		0	0
	ω<**1^g^**	3	4	all	5	5	0	-	-
	ω**=**	0.19	-	0.21	-	-		0.17	0.17
**Mammals**									
	ω>**1^f^**	0	0	1	0	2		0	0
	ω<**1^g^**	77	124	134	167	172	1	-	-
	ω**=**	0.06	-		-	-		0.05	0.07

**a**: Single Likelihood Ancestor Counting

**b**: Fixed-effects likelihood

**c**: Random-effects likelihood

**d**: Fast, Unconstrained Bayesian AppRoximation

**Integrative**: Sites detected in common by SLAC, FEL, REL, FUBAR and MEME

**e**: Sites detected as experiencing episodic diversifying selection (0.05 significance) by the Mixed Effects Model Evolution (MEME)

**M8**: Positively-selected sites detected using the Bayes Empirical Bayes approach implemented in M8. Sites detected at 0.99 and 0.95 significance are indicated in the parenthesis

**M2a**: Positively-selected sites detected using the Bayes Empirical Bayes approach implemented in M2a. Sites detected at 0.99 and 0.95 significance are indicated in the parenthesis

**f**: Number of positively selected sites at 0.05 significance (for SLAC, FEL) or 50 Bayes factor (for REL) / number of sites under pervasive diversifying selection at the posterior probability ≥0.9 (FUBAR)

**g**: Number of negatively selected sites at 0.05 significance (for SLAC, FEL) or 50 Bayes factor (for REL) / number of sites under pervasive purifying selection at the posterior probability ≥0.9 (FUBAR)

**ω**: mean dN/dS**NFF**: “non-front-fanged” advanced snakes

We further evaluated selection pressures along the elapid and viperid NGF lineages using two-ratio model (which assesses selection pressures only across lineages) and the branch-site test A (which assesses selection pressures across the sites and along the lineages). The two-ratio model failed (p>0.05) to detect positive selection in elapid (ω=0.85) and viperid NGF (ω=0.82). However, branch-site test A indicated a strong influence of positive selection on elapid NGF [ω = 3.90; 4.5% positively selected (PS) sites; significant at 0.001 after Bonferroni correction] and to a lesser extent on Viperidae NGF (ω = 2.01 and 8.1% of PS-sites; significant at 0.001 after Bonferroni correction: Table S4). We further employed the clade model c approach to simultaneously compute and compare the ω values of various Toxicofera NGF lineages (Table 1). Clade model analyses indicated the influence of positive selection in shaping the evolution of Elapidae NGF (ω=2.38), while the remaining lineages were found to be constrained by negative selection (but again with the caveat that fewer ‘non-front-fanged’ advanced snake and Toxicofera lizard venom-gland specific NGF sequences were available; Table 1). 

To derive further support for the sites detected as positively selected by the nucleotide analyses of elapid NGF, we employed a complementary amino acid-level approach implemented in TreeSAAP (Table 4). Since a mutated amino acid can share similar/identical biochemical and structural properties with the ancestral amino acid being replaced, not all non-synonymous mutations may affect the structure and function of a protein. Hence, the evaluation of the influence of selection on the biochemical and structural properties of proteins provides further insights about the strength of a mutation in affecting the fitness of an organism. With this combined approach, we were able to identify 13 positively selected sites (68% of positively selected sites) in elapid NGF (Table 4). Mapping of these mutations onto the three-dimensional structure of NGF revealed that a majority of detected hypermutable sites (74%) were concentrated in the secreted region (β-polypeptide chain) of Elapidae NGF (Figure S5). Mutation mapping also revealed that a greater proportion of amino acid residues (92%; remaining sites couldn’t be assigned to exposed/buried class) were exposed to the surrounding medium, indicating that these Elapid venom proteins evolve through focal mutagenesis (Table 4).

**Table 4:** Nucleotide and complementary protein-level selection assessment of Elapidae Nerve Growth Factors (NGF).

**Codon**	**Amino Aacid**	**M2a^a^**	**M8^b^**	**Property^c^**	**Magnitude^d^**	**ASA**
57	R	4.241±0.515	3.894±0.510	*-*	-	-
		(0.994)	(0.998)*			
72	A	4.260±0.453	3.899±0.493	*-*	-	-
		(0.999)**	(1.0)**			
**116**	**T**	**4.227±0.553**	**3.892±0.513**	***R_α_, α_m_***	**6, 7**	**-**
		(0.990)*	(0.997)**			
**123**	**A**	4.243±0.509	**3.895±0.506**	***R_α_, α_m_***	**6, 7**	**-**
		(0.994)	( 0.998)*			
125	R	4.132±0.769	3.857±0.601	*-*	-	-
		(0.961)*	(0.986)*			
**127**	**N**	**4.262±0.448**	**3.900±0.492**	***R_α_, α_m_***	**6, 7**	**-**
		(1.0)**	(1.0)**			
**133**	**Q**	**4.262±0.446**	**3.900±0.491**	***R_α_, α_m_***	**6, 7**	**-**
		(1.0)**	(1.0)**			
**144**	**D**	**4.258±0.459**	**3.899±0.495**	***R_α_, α_m_***	**6, 7**	**69.5**
		(0.999)**	(1.0)**			**Exposed**
**150**	**T**	**4.252±0.481**	**3.897±0.500**	***α_m_***	**6**	**77.5**
		(0.997)**	(0.999)**			**Exposed**
**155**	**R**	**4.146±0.740**	**3.867±0.576**	***α_m_***	**6**	**87.0**
		(0.965)*	(0.990)*			**Exposed**
158	V	4.247±0.498	3.896±0.504	*-*	-	**76.9**
		(0.995)**	(0.999)**			**Exposed**
**163**	**E**	**4.257±0.464**	**3.898±0.496**	***α_m_***	**6**	**65.9**
		(0.998)*	(0.999)**			**Exposed**
**167**	**L**	4.024±0.940	**3.824±0.670**	***α_m_***	**6**	**65.0**
		(0.929)	(0.976)*			**Exposed**
170	E	4.260±0.453	3.900±0.493	*-*	*-*	73.0
		(0.999)**	(1.0)**			Exposed
182	R	3.951±1.036	3.789±0.736	*-*	-	57.3
		(0.907)	(0.965)*			Exposed
**207**	**Q**	**4.255±0.471**	**3.898±0.499**	***R_α_***	**6**	**32.2**
		(0.998)**	0.999**			**NA**
**211**	**R**	**4.260±0.455**	**3.899±0.493**	***R_α_***	**6**	**53.1**
		(0.999)*	(1.0)**			**Exposed**
**219**	**Q**	**4.262±0.446**	**3.900±0.491**	***R_α_***	**6**	**73.8**
		(1.0)**	(1.0)**			**Exposed**
**240**	**D**	4.004±0.970	**3.804±0.712**	***R_α_***	**6**	**66.9**
		(0.923)	(0.969)*			**Exposed**
**167**	**L**	4.024±0.940	**3.824±0.670**	***α_m_***	**6**	**65.0**
		(0.929)	(0.976)*			**Exposed**
170	E	4.260±0.453	3.900±0.493	*-*	*-*	73.0
		(0.999)**	(1.0)**			Exposed
182	R	3.951±1.036	3.789±0.736	*-*	-	57.3
		(0.907)	(0.965)*			Exposed
**207**	**Q**	**4.255±0.471**	**3.898±0.499**	***R_α_***	**6**	**32.2**
		(0.998)**	0.999**			**NA**
**211**	**R**	**4.260±0.455**	**3.899±0.493**	***R_α_***	**6**	**53.1**
		(0.999)*	(1.0)**			**Exposed**
**219**	**Q**	**4.262±0.446**	**3.900±0.491**	***R_α_***	**6**	**73.8**
		(1.0)**	(1.0)**			**Exposed**
**240**	**D**	4.004±0.970	**3.804±0.712**	***R_α_***	**6**	**66.9**
		(0.923)	(0.969)*			**Exposed**

**Amino-acid** property symbols used: Power to be in the middle of α-helix (α_m_), Solvent accessible reduction ratio (**R_α_**)

PAML

**a, b**: Bayes Empirical Bayes (BEB) posterior probability and post-mean omega (indicated in brackets) for the sites detected as positively selected by the site models M2a and M8, respectively. Sites detected as positively selected at 0.95 and 0.99 posterior probability by the Bayes Empirical Bayes approach of M8 are represented by * and **, respectively.

TreeSAAP

**c**: amino acid property experiencing positive diversifying selection

**d**: magnitude of selection on the amino acid property

**ASA**: Accessible surface area.

Note: Codon sites with significant support from both nucleotide and protein-level selection analyses are highlighted in bold.

Evolutionary fingerprint analyses, which is based on the probability distribution of site-to-site synonymous (α or dS) and nonsynonymous (β or dN) substitution rates in an alignment, identified a significantly large proportion of sites in elapid NGF as evolving under the influence of positive selection, while the remaining reptilian and mammalian NGF, BDNF and NT-3 lineages were depicted as constrained by extreme negative selection pressures (Figure S6-S8). It is noteworthy that a small fraction of sites in all snake lineages (Caenophidia and Henophidia) seem to have accumulated a greater proportion of non-synonymous to synonymous mutations, although these observations lacked significant statistical support (indicated by the density and compactness of the circles). Branch-site REL identified four, three and two branches (p<0.05), respectively in elapid, viperid and ‘non-front-fanged’ advanced snake NGFs as evolving under the influence of episodic diversifying selection. This highlights the episodic nature of natural selection on the evolution of advanced snake venom-specific nerve growth factors (Figure S9). This test failed to identify positive diversifying selection in other non-venomous reptilian or mammalian NGF and any BDNF and NT-3 gene lineages, including those of the advanced snakes.

## Discussion

### Distinct rates and trajectories of neurotrophin evolution

Evidence provided by various analyses (codeml site, branch, branch-site and clade-specific models: M8, M2a, M3, M0, two-ratio model, branch-site test A, clade model c; HyPhy: SLAC, FEL, REL, MEME, FUBAR, integrative analyses, branch-site REL; TreeSAAP: amino acid-level selection assessment and the evolutionary fingerprint analysis) not only revealed the strong influence of positive diversifying selection on Elapidae venom NGF (and to a lesser extent on the venom NGF of other advanced snake lineages, particularly the Viperidae NGF), but also highlighted the role of purifying selection pressures in shaping the molecular evolution of various non-venomous reptilian and mammalian NGF lineages (Tables 1-3; Figures 1-5; Tables S1-S4; Figures 4 and 5; Figures S6-S9). The accumulation of variation in advanced snake NGFs, in a fashion similar to other venom proteins [5,7,8] is suggestive of a role in envenomation and prey-capture. As envenomation is their primary method of prey subjugation, elapid and viperid snakes rely on the swift and potent action of venom for foraging. Hence, most elapid and viperid venom components experience extreme selection pressures, likely as a result of a co-evolutionary arms race with their prey [5,7]. Although NGF constitutes a very small proportion (1-5 mg/g of venom) of the total venom in most snakes [29,41–43], the venom of snakes of the genus *Oxyuranus* (Taipans) has been shown to contain larger quantities of NGF [44–46]. NGF seems to make up nearly 0.5% (w/w) of the total venom injected by these snakes [44–46]. Thus, NGF could be secreted in elevated amounts by several species of caenophidian snakes, which could be enough to cause toxicity in the prey. ‘Non-front-fanged’ advanced snakes have been shown to possess several rapidly evolving venom components that participate in prey envenoming [47–52]. Selection analyses in the present study identify several residues and branches in both the ‘non-front-fanged’ and viperid advanced snake NGF lineages as evolving under positive Darwinian selection (Table 1: integrative approach; Table S4: branch-site and clade models; Figure S6, S9b and S9c). However, due to the scarcity of NGF sequences from both these lineages, the extent to which NGF participates in their envenoming remains elusive.

The aforementioned analyses also demonstrated the influence of negative selection on the evolution of all reptilian and mammalian BDNF and NT-3 gene lineages examined in this study. The lack of variation in these genes despite their origin over 300 million years ago is probably due to their significance in neuronal development, maintenance and survival. NGF, BDNF and NT-3 deficiencies have been correlated with severe peripheral neuropathy and death of the organism [53].

### Focal mutagenesis of venom nerve growth factors

The synthesis and secretion of venom proteins is an energetically expensive process [54–56]. Hence, mutations that disrupt the structure/function of proteins are filtered out of the population by negative selection pressures, favouring the conservation of catalytic and structurally important core residues. As a result, most mutations in proteins can be found concentrated in structurally and/or functionally unimportant regions. Moreover, the accumulation of point mutations under the influence of positive Darwinian selection in certain regions of the toxin, such as the molecular surface (a phenomenon we refer to as focal mutagenesis), may confer adaptive significance. We and others have postulated that predatory venom components undergo focal mutagenesis that results in the generation of a diversity of novel amino acids (and their side-chains) on the molecular surface, which could *non-specifically* interact with prey cells and cause a myriad of pharmacological effects [11,52,57–61]. Mapping of hypermutable sites onto the three-dimensional homology model of elapid NGFs and the calculation of accessible surface area (ASA) ratio for each residue indicated that 92% of the positively selected residues in these proteins are located on the molecular surface (Table 4; remaining sites couldn’t be conclusively assigned to exposed/buried class). Moreover, a large proportion of these positively selected sites (74%) were found concentrated in the β-chain of Elapidae NGF, which is the only secreted region of the mature toxin and hence the only region that is likely to experience a coevolutionary arms race (Figure S5). Thus, it is highly likely that elapid NGFs follow the regime of focal mutagenesis and favour the accumulation of hypermutable sites in specific regions of the secreted toxin that are able to influence the fitness of the animal. Rapidly evolving caenophidian NGFs with a diversity of amino acids on their molecular surface could not only induce a plethora of pharmacological effects by non-specifically interacting with novel receptors of the prey, but could further aid in evading the prey immune response [62].

### Putative role of nerve growth factors in venom

Nerve growth factors are known to inhibit venom metalloproteinase-dependent proteolysis [63]. Hence, the presence of large amounts of NGF, relative to metalloproteinases in viper venom [64,65] has been viewed as an indication of their role in prevention of venom autolysis [63]. However, additional plausible mechanisms of action exist for both the direct and indirect participation of rapidly evolving caenophidian NGF in prey envenoming, potentially resulting in an increase in the potency of the toxicoferan venom arsenal. The injection of large amounts of NGF into the lymphatic system of prey would result in NGF overdose, triggering a variety of reactions. Nerve growth factors are also known to cause apoptosis of cells lacking the expression of TrkA receptors [66–68] and thus the caenophidian venom NGFs could act as cytotoxic proapoptotic factors, after being introduced into tissues where they are not typically found. Venom components like hyaluronidase have been shown to degrade hyaluronic acid in the extra-cellular matrix and function as spreading factors, facilitating the easier diffusion of other venom components [69]. NGF, which is known to trigger the release of granules containing histamines, serotonins and other chemical mediators from mastocytes [70,71], could perform a similar ancillary venom function by facilitating the efficient absorption of other venom components. The degranulation of mast cells that are located primarily in perivascular spaces, often close to neurons and blood vessels, has been correlated with increased vascular permeability and even neurogenic inflammations [72]. While controlled degranulation aids in orchestrating and mounting acute inflammatory reactions, massive releases can be associated with anaphylaxis, bronchoconstriction (suffocation) and vasodilation [73]. Over the years, both glycosylated and non-glycosylated forms of NGFs have been reported from snake venoms [44,74–76]. Glycosylation has been proposed to prolong the circulation time of serum proteins by increasing their stability [77]. Interestingly, Asn 23, which has been postulated as a putative glycosylation site in mature venom NGF [78,79], is highly conserved in the entire squamate lineage (except in a few elapid snakes: genus *Naja*, *Notechis scutatus*, *Pseudechis australis*, *Pseudechis porphyriacus*, etc.), while being completely absent from mammalian and turtle NGFs (Figure S5). These observations are not surprising since mammalian NGFs are not known to undergo glycosylation. Snake venom NGFs could undergo posttranslational glycosylation in order to circulate longer in the bloodstream of the prey and spread further throughout the prey animal’s system. Thus, caenophidian NGFs could directly and/or indirectly participate in prey-envenoming through a number of plausible mechanisms.

### Putative duplication of nerve growth factors in *Elapidae*

Genes encoding venom proteins are known to evolve via the birth-and-death model of evolution where new genes are created by repeated duplication events, and are subsequently either maintained in the genome, deleted or become non-functional pseudogenes [7,8]. The molecular phylogeny of NGFs clearly indicates the presence of two independent NGF genes in *Naja sputatrix*, with very strong support for the node separating them (915 bootstrap and 1.0 Bayesian posterior probability: Figure 1). The phylogenetic placement of these two gene homologues suggests that the gene duplication event responsible for their origin occurred in an early elapid ancestor (Figure 1) – we might therefore expect to find additional copies of NGF in other elapid snakes in the future. Although one might initially assume that the original copy of NGF would continue the ancestral role in homeostasis, thereby releasing the novel duplicate gene to evolve a function specific to venom, in *N. sputatrix* both NGF genes were found to be expressed in the venom gland [80]. However, considering how important NGF and other neurotrophins appear to be for the survival of an organism [53], it is highly unlikely that both gene copies would be exclusively associated with a role in prey envenoming. We therefore propose that co-expression of an NGF gene occurs in different tissue types, similar to that recently proposed for other toxin families [81]. Specifically, we postulate that despite the elevated mutation rate in the Elapidae NGF, the ancestral copy of the gene has retained its biochemical functions by undergoing focal mutagenesis and continues to ensure homeostasis. A greater accumulation of mutations on the molecular surface and the loops of the protein ensures the preservation of structurally and functionally important core residues. Clearly, a large number of residues across the entire vertebrate NGF lineage appear to be highly conserved (Figure S5). Moreover, it has been demonstrated on numerous occasions that snake venom NGFs exhibit biological activities similar to their mammalian counterparts [44,74]. This would seemingly free the duplicate elapid NGF gene to participate exclusively in prey envenoming. In contrast to the Elapidae NGF, only one copy was found in the venom glands of the Anguimorpha lizard *Abronia graminea*, which was identical to the one recovered from genomic sequencing for use in taxonomy studies [27]. Moreover, despite extensive BLAST searches using sequence templates from a wide array of mammalian and reptilian NGFs against the genomes of *Anolis carolinensis* (Iguania) and *Python molurus* (Henophidia), we were only able to retrieve a single copy of the NGF gene. Hence, we further speculate that the ancestral NGF gene is co-expressed in physiological tissues and the venom-gland, while the new gene duplicates are more likely to be tissue-specifically expressed in the venom gland alone. Experimental investigation in the future regarding the copy number of NGF should reveal if the NGF duplication occurred in an early elapid ancestral lineage or at the base of the Toxicofera phylogenetic tree.

### Note on the usage of NGF in the phylogenetic reconstructions of Toxicofera reptiles

NGF is a commonly used gene in reptilian phylogenetic studies [82–85]. However, the detection of gene duplication events and the accelerated evolution of the advanced snake NGFs indicate that these genes should not be employed in phylogenetic inferences of Toxicofera reptiles, as these factors can undermine such analyses [86].

## Conclusions

In this study, we show that BDNF and NT-3, two major members of the neurotrophin family, lack variations completely and evolve under the regime of negative selection, indicating their extreme importance in the maintenance of homeostasis. In complete contrast, this study points for the first time towards venom-specific NGF evolving in a manner typical of toxic proteins and consequently suggests a hitherto unappreciated role in envenomation. We highlight an exquisite mechanism of venom evolution where focal mutagenesis has facilitated the transformation of a key regulatory protein into a toxin, without hampering its participation in homeostasis. Structure-function studies of mutant NGF may reveal a suite of novel activities, some of which may be of use in drug design and development.

## Materials and Methods

### Phylogenetic Analyses

Phylogenetic analyses were performed to allow the reconstruction of the molecular evolutionary history of vertebrate NGF, BDNF and NT-3 genes. Nucleotide sequences were downloaded from the National Center for Biotechnology Information database (NCBI: http://www.ncbi.nlm.nih.gov/) and a dataset of 1183 sequences (NGF: 308; BDNF: 434; NT-3: 441) was compiled. Accession numbers of all sequences analysed in this study are available in Tables S5.1-S5.3. Unpublished sequences are available in the File S1. Resultant sequence sets were aligned using MUSCLE [87]. The phylogenetic relationships were determined using Bayesian and maximum-likelihood approaches. MrBayes version 3.2 [88] was used for Bayesian inference. Tree searches were run using four Markov chains for a minimum of 10 million generations, sampling every 100th tree. The log likelihood score of each saved tree was plotted against the number of generations to establish the point at which the log-likelihood scores of the analyses reached their asymptote. 25% of the total trees sampled were conservatively discarded as burnin. The posterior probabilities for clades were established by constructing a majority rule consensus tree for all trees generated after the completion of the burnin. The analyses were repeated three times to make sure that the trees generated were not clustered around local optima. An optimal maximum likelihood phylogenetic tree was obtained using PhyML 3.0 [89] and node support was evaluated with 1,000 bootstrapping replicates.

### Test for Recombination

To overcome the effects of recombination on the phylogenetic and evolutionary interpretations [90], we employed Single Breakpoint algorithm implemented in the HyPhy package and assessed recombination on all the toxin forms examined in this study [91,92]. When potential breakpoints were detected using the small sample Akaike information Criterion (AICc), the sequences were compartmentalized before conducting selection analyses, so as to allow recombining units to have distinct phylogeny. 

### Selection Analyses

We evaluated the influence of natural selection on various members of vertebrate neurotrophins using maximum-likelihood models [93,94] implemented in CODEML of the PAML [95]. In order to detect evolutionary selection pressures acting upon individual lineages, we employed the two-ratio model as well as the optimized branch-site test [96,97]. The two-ratio model evaluates selection across the lineages alone, while the branch-site model allows ω to vary across the sites of the protein and along the branches in the tree. The latter is known for its reasonable power and accuracy to detect short bursts of episodic adaptations [97]. However, both the two-ratio and branch-site models require the foreground (lineages under positive selection) and background lineages (lineages lacking positive selection) to be defined *a priori*. Such predefined biological hypotheses are often unavailable and it becomes difficult to define the foreground lineages. Therefore, we treated each clade being compared as foreground branches alternatively and tested multiple hypotheses. A likelihood-ratio test was then conducted by comparing the model that allows ω to be greater than 1 in the foreground branch, with a null model that does not (ω constrained 1). It has been suggested that while implementing multiple hypotheses using branch and branch-site models, it is necessary to control the family-wise error rate (FWER or Type I error) [97]. We used Bonferroni correction to account for such errors. It uses α/*n* as the significance level to test each hypothesis; where ‘α’ is the significance level and ‘*n*’ is the number of independent true null hypotheses. We further utilized branch-site REL implemented in HyPhy to identify lineages affected by positive selection pressures. Unlike the aforementioned lineage-specific models, branch-site REL does not require the identification of foreground and background branches *a priori*.

Because lineage-specific models either assume a single ω for the entire tree or assess the effects of selection only along the branches, they often fail to identify regions in proteins that might be affected by episodic selection pressures and ultimately underestimate the strength of selection. Hence, we employed site-specific models which estimate positive selection statistically as a non-synonymous-to-synonymous nucleotide-substitution rate ratio (ω) significantly greater than 1. We compared likelihood values for three pairs of models with different assumed ω distributions as no a priori expectation exists for the same: M0 (constant ω rates across all sites) versus M3 (allows the ω to vary across sites within ‘*n*’ discrete categories, *n* ≥ 3); M1a (a model of neutral evolution) where all sites are assumed to be either under negative (ω < 1) or neutral selection (ω = 1) versus M2a (a model of positive selection) which in addition to the site classes mentioned for M1a, assumes a third category of sites; sites with ω >1 (positive selection) and M7 (β) versus M8 (β and ω), and models that mirror the evolutionary constraints of M1 and M2 but assume that ω values are drawn from a β distribution [98]. Only if the alternative models (M3, M2a and M8: allow sites with ω >1) show a better fit in Likelihood Ratio Test (LRT) relative to their null models (M0, M1a and M7: do not allow sites ω >1), are their results considered significant. LRT is estimated as twice the difference in maximum likelihood values between nested models and compared with the χ^2^ distribution with the appropriate degree of freedom (i.e., the difference in the number of parameters between the two models). The Bayes empirical Bayes (BEB) approach [97] was used to identify amino acids under positive selection by calculating the posterior probabilities that a particular amino acid belongs to a given selection class (neutral, conserved or highly variable). Sites with greater posterior probability (PP ≥ 95%) of belonging to the ‘ω > 1 class’ were inferred to be positively selected.

SLAC, FEL, REL and FUBAR [99,100] implemented in HyPhy [101] were employed to provide additional support to the aforementioned analyses and to detect sites evolving under the influence of positive and negative selection. MEME [102] was also used to detect episodic diversifying selection. Further support for the results of the nucleotide-level selection analyses was obtained and the radicalness of mutations were assessed using a complementary protein-level approach implemented in TreeSAAP [103].

Direct comparison of ω values computed from different datasets can be misleading, as they can have different proportion of sites under selection. Hence, we assessed the selection pressures shaping the evolution of NGF along various toxicoferan reptilian lineages by employing clade model analyses implemented in codeml and simultaneously estimated ω values [104]. The significance of the analysis was tested by comparing the likelihood of this model with that of model M1a. To clearly depict the proportion of sites under different regimes of selection, an evolutionary fingerprint analysis was carried out using the evolutionary selection distance (ESD) algorithm implemented in datamonkey [91,105,106]. Evolutionary fingerprint analysis fits a versatile general discrete bivariate model of site-to-site variation in selection pressures and comprises a description of the number of selective classes, the dN/dS (ω) ratio for each class and the assignment of sites to classes [91,106].

### Structural Analyses

To depict the natural selection pressures influencing the evolution of various neurotrophins, we mapped the sites under positive selection on the homology models created using Phyre 2 webserver [107]. Pymol 1.3 [108] was used to visualize and generate the images of homology models. The consurf webserver [109] was used for mapping the evolutionary selection pressures on the three-dimensional homology models. GETAREA [110] was used to calculate the ASA ratio or the solvent exposure of amino-acid side chains. It uses the atom co-ordinates of the PDB file and indicates if a residue is buried or exposed to the surrounding medium by comparing the ratio between side-chain ASA and the “random coil” values per residue. An amino-acid is considered to be buried if ASA is less than 20% and exposed if ASA is ≥ 50%.

## Supporting Information

Table S1 **(S1.1 – S1.11) Details of selection analyses of nerve growth factors (NGF).** a: dn/ds (weighted average). b: Significance of the model in comparison with the null model. c: Number of sites with ω > 1 under the Bayes empirical Bayes approach with a posterior probability (PP) more than or equal to 0.99 and 0.95. * Models which allow ω > 1.
(PDF)

Table S2 **(S2.1 – S2.11) Details of selection analyses of brain-derived neurotrophic factors (BDNF).** a: dn/ds (weighted average). b: Significance of the model in comparison with the null model. c: Number of sites with ω > 1 under the Bayes empirical Bayes approach with a posterior probability (PP) more than or equal to 0.99 and 0.95. * Models which allow ω > 1.
(PDF)

Table S3 **(S3.1-S3.11) Details of selection analyses of neurotrophin-3 (NT3).** a: dn/ds (weighted average). b: Significance of the model in comparison with the null model. c: Number of sites with ω > 1 under the Bayes empirical Bayes approach with a posterior probability (PP) more than or equal to 0.99 and 0.95. * Models which allow ω > 1.
(PDF)

Table S4 **Lineage-specific selection analyses of nerve growth factors (NGF).** a: dn/ds (weighted average). b: Significance of the model in comparison with the null model. * Significant after Bonferroni correction. NS: Not significant. Significantly detected positively selected lineages are highlighted in bold.
(PDF)

Table S5 **(S5.1-S5.3) Sequences analysed.**
(PDF)

Figure S1 **Maximum-likelihood molecular phylogeny of nerve growth factors (NGF).** Branches with bootstrap support of less than 850 (out of 1000 bootstrap replicates) are highlighted in grey. [NFF: “non-front-fanged” advanced snakes; Atr: Atractaspidinae ; Sci: Scinciformata].
(PDF)

Figure S2 **Maximum-likelihood molecular phylogeny of brain-derived neurotrophic factors (BDNF).** Branches with bootstrap support of less than 850 (out of 1000 bootstrap replicates) are highlighted in grey.
(PDF)

Figure S3 **Maximum-likelihood molecular phylogeny of neurotrophin-3 (NT3).** Branches with bootstrap support of less than 850 (out of 1000 bootstrap replicates) are highlighted in grey.
(PDF)

Figure S4 **Maximum-likelihood phylogeny of neurotrophins.** Branches with bootstrap support of less than 850 (out of 1000 bootstrap replicates) are highlighted in grey. [NFF: ‘non-front-fanged’ advanced snakes; Pyt: Pythonidae; Mam: Mammals].
(PDF)

Figure S5 **Alignment of vertebrate nerve growth factors.**
(PDF)

Figure S6 **Evolutionary fingerprint of nerve growth factors (NGF).** Estimates of the distribution of synonymous (a) and non-synonymous (b) substitution ratesinferred for various reptilian and mammalian nerve growth factor (NGF) lineages are shown here. The ellipses reflect a Gaussian-approximated variance in each individual rate estimate, and coloured pixels show the density of the posterior sample of the distribution for a given rate. The diagonal line represents the idealized neutral evolution regime (ω = 1), points above and below the line correspond to positive selection (ω>1) and negative selection (ω<1), respectively. Site model 8 omega (w) along with the total number of positively selected sites detected by its Bayes Empirical Bayes (BEB) approach are also indicated below.
(PDF)

Figure S7 **Evolutionary fingerprint of brain-derived neurotrophic factors (BDNF).** Estimates of the distribution of synonymous (a) and non-synonymous (b) substitution rates inferred for various reptilian and mammalian brain-derived neurotrophic factor (BDNF) lineages are shown here. The ellipses reflect a Gaussian-approximated variance in each individual rate estimate, and coloured pixels show the density of the posterior sample of the distribution for a given rate. The diagonal line represents the idealized neutral evolution regime (ω = 1), points above and below the line correspond to positive selection (ω>1) and negative selection (ω<1), respectively. Site model 8 omega (w) along with the total number of positively selected sites detected by its Bayes Empirical Bayes (BEB) approach are also indicated below.
(PDF)

Figure S8 **Evolutionary fingerprint of neurotrophin-3 (NT3).** Estimates of the distribution of synonymous (a) and non-synonymous (b) substitution rates inferred for various reptilian and mammalian neurotrophin-3 (NT-3) lineages are shown here. The ellipses reflect a Gaussian-approximated variance in each individual rate estimate, and coloured pixels show the density of the posterior sample of the distribution for a given rate. The diagonal line represents the idealized neutral evolution regime (ω = 1), points above and below the line correspond to positive selection (ω>1) and negative selection (ω<1), respectively. Site model 8 omega (w) along with the total number of positively selected sites detected by its Bayes Empirical Bayes (BEB) approach is also indicated below.
(PDF)

Figure S9 **Branch-site REL: Caenophidian nerve growth factors (NGF).** The hue of each colour indicates strength of selection, with primary red corresponding to ω > 5, primary blue to ω = 0 and grey to ω=1. The width of each colour component represents the proportion of sites in the corresponding class. Thicker branches have been classified as undergoing episodic diversifying selection (indicated by arrows) by the sequential likelihood ratio test at corrected p ≤ 0.05.
(PDF)

File S1 **Unpublished sequences.**
(DOCX)
