# Correction: Molecular Evolution of Vertebrate Neurotrophins: Co-Option of the Highly Conserved Nerve Growth Factor Gene into the Advanced Snake Venom Arsenalf

**DOI:** 10.1371/annotation/accecc73-91b2-45d4-bb33-774b1f394ca1

**Published:** 2013-12-17

**Authors:** Kartik Sunagar, Bryan Grieg Fry, Timothy N. W. Jackson, Nicholas R. Casewell, Eivind A. B. Undheim, Nicolas Vidal, Syed A. Ali, Glenn F. King, Karthikeyan Vasudevan, Vitor Vasconcelos, Agostinho Antunes

The word "arsenal" was misspelled in the title of the article. The correct title is: "Molecular Evolution of Vertebrate Neurotrophins: Co-Option of the Highly Conserved Nerve Growth Factor Gene into the Advanced Snake Venom Arsenal." The correct Citation is: Sunagar K, Fry BG, Jackson TNW, Casewell NR, Undheim EAB, et al. (2013) Molecular Evolution of Vertebrate Neurotrophins: Co-Option of the Highly Conserved Nerve Growth Factor Gene into the Advanced Snake Venom Arsenal. PLoS ONE 8(11): e81827. doi:10.1371/journal.pone.0081827.

