# Supplementary material for: Molecular Evolution of Vertebrate Neurotrophins: Co-Option of the Highly Conserved Nerve Growth Factor Gene into the Advanced Snake Venom Arsenalf
Source: PLoS One. 2013 Nov 29;8(11):e81827. doi: 10.1371/journal.pone.0081827 (PMC3843689; doi:10.1371/journal.pone.0081827)
Supplement: Table S1 — (S1.1 – S1.11) Details of selection analyses of nerve growth factors (NGF). a: dn/ds (weighted average). b: Significance of the model in comparison with the null model. c: Number of sites with ω > 1 under the Bayes empirical Bayes approach with a posterior probability (PP) more than or equal to 0.99 and 0.95. * Models which allow ω > 1. (PDF) [file pone.0081827.s001.pdf]

Table S1.1 Maximum-likelihood parameter estimates for Elapidae nerve growth factors

Model	Likelihood (ι)	ω_0[a]	Parameters	Sign.[b]	No. of Sites with $\omega > 1$[c]
					**B.E.B**
**M0 (One ratio)**	-4971.215787	0.86	$= \omega_0$		-
**M1 (Neutral)**	-4872.409131	0.46	P_0: 0.622<br>ω_0: 0.14<br>P_1: 0.377<br>ω1: 1.0		-
**M2 (Selection)***	-4806.688787	0.94	P_0: 0.544<br>ω_0: 0.19<br>P_1: 0.335<br>ω_1:1.0<br>P_2: 0.119<br>ω_2: 4.23	$P < 0.05$	13 (PP ≥ 0.99)<br>3 (P ≥ 0.95)
**M3 (Discrete)***	-4806.119053	0.97	P_0: 0.633<br>ω_0: 0.24<br>P_1: 0.261<br>ω_1: 1.31<br>P_2: 0.104<br>ω_2: 4.56	$P << 0.001$	-
**M7 (beta)**	-4884.775245	0.50	p: 0.27770<br>q: 0.27293		-
**M8 (beta and ω)***	-4808.052643	0.95	p_0: 0.87<br>p: 0.538<br>q: 0.574<br>p1: 0.130<br>ω: 4.09	$P < 0.05$	14 (PP ≥ 0.99)<br>5 (P > 0.95)

Legend:

a: dn/ds (weighted average)

b: Significance of the model in comparison with the null model

c: Number of sites with $\omega > 1$ under the Bayes empirical Bayes approach with a posterior probability (PP) more than or equal to 0.99 and 0.95

* Models which allow $\omega > 1$

Table S1.2 Maximum-likelihood parameter estimates for Viperidae nerve growth factors

Model	Likelihood (ι)	ω_0[a]	Parameters	Sign.[b]	No. of Sites with $\omega > 1$[c]
					**B.E.B**
**M0 (One ratio)**	-2096.277194	0.72	$= \omega_0$		-
**M1 (Neutral)**	-2088.212820	0.64	P_0: 0.359<br>ω_0: 0.0<br>P_1: 0.640<br>ω1:1.0		-
**M2 (Selection)***	-2087.182712	0.77	P_0: 0.361<br>ω_0: 0.0<br>P_1: 0.538<br>ω_1:1.0<br>P_2: 0.099<br>ω_2:2.39	$P > 0.05^{N.S}$	0 (PP ≥ 0.99)<br>0 (P ≥ 0.95)
**M3 (Discrete)***	-2087.182664	0.77	P_0: 0.362<br>ω_0: 0.0<br>P_1: 0.542<br>ω_1: 1.0<br>P_2: 0.094<br>ω_2: 2.42	P << 0.001	-
**M7 (beta)**	-2088.414645	0.60	p: 0.00811<br>q: 0.00500		-
**M8 (beta and ω)***	-2087.182783	0.77	p_0: 0.902<br>p: 0.007<br>q: 0.005<br>p1: 0.0976<br>ω: 2.40	$P > 0.05^{N.S}$	0 (PP ≥ 0.99)<br>0 (P > 0.95)

Legend:

a: dn/ds (weighted average)

b: Significance of the model in comparison with the null model

c: Number of sites with ω > 1 under the Bayes empirical Bayes approach with a posterior probability (PP) more than or equal to 0.99 and 0.95

* Models which allow ω > 1

$P > 0.05^{N.S}$: Not significant at 0.05

Table S1.3 Maximum-likelihood parameter estimates for 'non-front-fanged' advanced snake nerve growth factors

Model	Likelihood (ι)	ω_0[a]	Parameters	Sign.[b]	No. of Sites with $\omega > 1$[c]
					**B.E.B**
**M0 (One ratio)**	-1627.071386	0.54	$= \omega_0$		-
**M1 (Neutral)**	-1618.386483	0.51	P_0: 0.487<br>ω_0: 0.0<br>P_1: 0.512<br>ω1: 1.0		-
**M2 (Selection)***	-1617.549643	0.58	P_0: 0.561<br>ω_0: 0.07<br>P_1: 0.373<br>ω_1:1.0<br>P_2: 0.065<br>ω_2: 2.63	P << 0.001	0 (PP ≥ 0.99)<br>0 (P ≥ 0.95)
**M3 (Discrete)***	-1617.506927	0.58	P_0: 0.419<br>ω_0: 0.0<br>P_1: 0.481<br>ω_1: 0.71<br>P_2: 0.099<br>ω_2: 2.41	P << 0.001	-
**M7 (beta)**	-1618.399985	0.50	p: 0.01595<br>q: 0.01445		-
**M8 (beta and ω)***	-1617.527922	0.58	p_0: 0.917<br>p: 0.179<br>q: 0.253<br>p1: 0.082<br>ω: 2.50	P << 0.001	0 (PP ≥ 0.99)<br>0 (P > 0.95)

Legend:

a: dn/ds (weighted average)

b: Significance of the model in comparison with the null model

c: Number of sites with ω > 1 under the Bayes empirical Bayes approach with a posterior probability (PP) more than or equal to 0.99 and 0.95

* Models which allow ω > 1

Table S1.4 Maximum-likelihood parameter estimates for Henophidia nerve growth factors

**Model**	**Likelihood (ι)**	**ω_0**[a]	**Parameters**	**Sign.**[b]	**No. of Sites with ω > 1**[c]
					**B.E.B**
**M0 (One ratio)**	-1515.110043	0.28	$= \omega_0$		-
**M1 (Neutral)**	-1504.271757	0.29	P_0: 0.726<br>ω_0: 0.02<br>P_1: 0.273<br>ω1: 1.0		-
**M2 (Selection)***	-1504.231832	0.30	P_0: 0.767<br>ω_0: 0.0<br>P_1: 0.370<br>ω_1:1.0<br>P_2: 0.232<br>ω_2: 1.13	P << 0.001	0 (PP ≥ 0.99)<br>0 (P > 0.95)
**M3 (Discrete)***	-1504.231832	0.30	P_0: 0.512<br>ω_0: 0.04<br>P_1: 0.254<br>ω_1: 0.04<br>P_2: 0.232<br>ω_2: 1.13	P << 0.001	-
**M7 (beta)**	-1504.332117	0.30	p: 0.03702<br>q: 0.08621		-
**M8 (beta and ω)***	-1504.235244	0.30	p_0: 0.767<br>p: 5.124<br>q: 99.0<br>p1: 0.232<br>ω: 1.13	P << 0.001	0 (PP ≥ 0.99)<br>0 (P > 0.95)

Legend:

a: dn/ds (weighted average)

b: Significance of the model in comparison with the null model

c: Number of sites with ω > 1 under the Bayes empirical Bayes approach with a posterior probability (PP) more than or equal to 0.99 and 0.95

* Models which allow ω > 1

Table S1.5 Maximum-likelihood parameter estimates for Iguania nerve growth factors

Model	Likelihood (ι)	ω_0[a]	Parameters	Sign.[b]	No. of Sites with $\omega > 1$[c]
					**B.E.B**
**M0 (One ratio)**	-3790.346051	0.24	$= \omega_0$		-
**M1 (Neutral)**	-3702.296471	0.29	P_0: 0.750<br>ω_0: 0.05<br>P_1: 0.249<br>ω1: 1.0		-
**M2 (Selection)***	-3702.296471	0.29	P_0: 0.750<br>ω_0: 0.05<br>P_1: 0.162<br>ω_1:1.0<br>P_2: 0.086<br>ω_2: 1.0	P << 0.001	0 (PP ≥ 0.99)<br>0 (P > 0.95)
**M3 (Discrete)***	-3700.970435	0.25	P_0: 0.431<br>ω_0: 0.05<br>P_1: 0.301<br>ω_1: 0.05<br>P_2: 0.266<br>ω_2: 0.83	P << 0.001	-
**M7 (beta)**	-3705.105730	0.25	p: 0.19627<br>q: 0.57899		-
**M8 (beta and ω)***	-3704.801826	0.25	p_0: 0.996<br>p: 0.201<br>q: 0.607<br>p1: 0.003<br>ω: 2.77	P << 0.001	0 (PP ≥ 0.99)<br>0 (P > 0.95)

Legend:

a: dn/ds (weighted average)

b: Significance of the model in comparison with the null model

c: Number of sites with ω > 1 under the Bayes empirical Bayes approach with a posterior probability (PP) more than or equal to 0.99 and 0.95

* Models which allow ω > 1

Table S1.6 Maximum-likelihood parameter estimates for Anguimorpha nerve growth factors

Model	Likelihood (ι)	ω_0[a]	Parameters	Sign.[b]	No. of Sites with ω > 1[c]
					**B.E.B**
**M0 (One ratio)**	-1825.008075	0.31	$= \omega_0$		-
**M1 (Neutral)**	-1817.374921	0.36	P_0: 0.684<br>ω_0: 0.075<br>P_1: 0.315<br>ω1: 1.0		-
**M2 (Selection)***	-1817.374921	0.36	P_0: 0.684<br>ω_0: 0.07<br>P_1: 0.232<br>ω_1:1.0<br>P_2: 0.082<br>ω_2: 1.0	$P << 0.001$	0 (PP ≥ 0.99)<br>0 (P > 0.95)
**M3 (Discrete)***	-1815.825499	0.31	P_0: 0.506<br>ω_0: 0.0<br>P_1: 0.252<br>ω_1: 0.64<br>P_2: 0.240<br>ω_2: 0.64	$P << 0.001$	-
**M7 (beta)**	-1816.464744	0.33	p: 0.21976<br>q: 0.44133		-
**M8 (beta and ω)***	-1816.464750	0.33	p_0: 0.999<br>p: 0.219<br>q: 0.441<br>p1: 0.00001<br>ω: 1.0	$P << 0.001$	0 (PP ≥ 0.99)<br>0 (P > 0.95)

Legend:

a: dn/ds (weighted average)

b: Significance of the model in comparison with the null model

c: Number of sites with ω > 1 under the Bayes empirical Bayes approach with a posterior probability (PP) more than or equal to 0.99 and 0.95

* Models which allow ω > 1

Table S1.7 Maximum-likelihood parameter estimates for Gekkota nerve growth factors

Model	Likelihood (ι)	ω_0[a]	Parameters	Sign.[b]	No. of Sites with ω > 1[c]
					**B.E.B**
**M0 (One ratio)**	-1612.059249	0.23	= ω_0		-
**M1 (Neutral)**	-1598.686340	0.22	P_0: 0.838<br>ω_0: 0.08<br>P_1: 0.161<br>ω1: 1.0		-
**M2 (Selection)***	-1597.762199	0.25	P_0: 0.858<br>ω_0: 0.095<br>P_1: 0.128<br>ω_1:1.0<br>P_2: 0.013<br>ω_2: 3.70	$P << 0.001$	0 ($PP \geq 0.99$)<br>0 ($P > 0.95$)
**M3 (Discrete)***	-1597.734005	0.25	P_0: 0.811<br>ω_0: 0.07<br>P_1: 0.172<br>ω_1: 0.78<br>P_2: 0.015<br>ω_2: 3.56	$P << 0.001$	-
**M7 (beta)**	-1599.515007	0.23	p: 0.15007<br>q: 0.48881		-
**M8 (beta and ω)***	-1597.805828	0.25	p_0: 0.980<br>p: 0.311<br>q: 1.25<br>p1: 0.019<br>ω: 3.33	$P << 0.001$	0 ($PP \geq 0.99$)<br>0 ($P > 0.95$)

Legend:

a: dn/ds (weighted average)

b: Significance of the model in comparison with the null model

c: Number of sites with ω > 1 under the Bayes empirical Bayes approach with a posterior probability (PP) more than or equal to 0.99 and 0.95

* Models which allow ω > 1

Table S1.8 Maximum-likelihood parameter estimates for Scinciformata nerve growth factors

Model	Likelihood (ι)	ω_0[a]	Parameters	Sign.[b]	No. of Sites with ω > 1[c]
					**B.E.B**
**M0 (One ratio)**	-1704.723234	0.20	$= \omega_0$		-
**M1 (Neutral)**	-1701.003298	0.25	P_0: 0.818<br>ω_0: 0.09<br>P_1: 0.181<br>ω1: 1.0		-
**M2 (Selection)***	-1701.003298	0.25	P_0: 0.531<br>ω_0: 0.0<br>P_1: 0.241<br>ω_1:0.46<br>P_2: 0.227<br>ω_2: 0.46	P << 0.001	0 (PP ≥ 0.99)<br>0 (P > 0.95)
**M3 (Discrete)***	-1697.979157	0.21	P_0: 0.343<br>ω_0: 0.0<br>P_1: 0.592<br>ω_1: 2.93<br>P_2: 0.064<br>ω_2: 13.44	P << 0.001	-
**M7 (beta)**	-1698.961126	0.22	p: 0.34022<br>q: 1.17860		-
**M8 (beta and ω)***	-1698.961169	0.22	p_0: 0.999<br>p: 0.340<br>q: 1.178<br>p1: 0.00001<br>ω: 1.0	P << 0.001	0 (PP ≥ 0.99)<br>0 (P > 0.95)

Legend:

a: dn/ds (weighted average)

b: Significance of the model in comparison with the null model

c: Number of sites with ω > 1 under the Bayes empirical Bayes approach with a posterior probability (PP) more than or equal to 0.99 and 0.95

* Models which allow ω > 1

Table S1.9 Maximum-likelihood parameter estimates for Laterata nerve growth factors

Model	Likelihood (ι)	ω_0[a]	Parameters	Sign.[b]	No. of Sites with $\omega > 1$[c]
					**B.E.B**
**M0 (One ratio)**	-1687.358175	0.24	$= \omega_0$		-
**M1 (Neutral)**	-1671.898713	0.27	P_0: 0.766<br>ω_0: 0.05<br>P_1: 0.233<br>ω1: 1.0		-
**M2 (Selection)***	-1671.898713	0.27	P_0: 0.766<br>ω_0: 0.05<br>P_1: 0.166<br>ω_1:1.0<br>P_2: 0.067<br>ω_2: 1.0	$P << 0.001$	0 ($PP \geq 0.99$)<br>0 ($P > 0.95$)
**M3 (Discrete)***	-1671.696617	0.26	P_0: 0.167<br>ω_0: 0.03<br>P_1: 0.538<br>ω_1: 0.03<br>P_2: 0.294<br>ω_2: 0.80	$P << 0.001$	-
**M7 (beta)**	-1671.866588	0.26	p: 0.13468<br>q: 0.37099		-
**M8 (beta and ω)***	-1671.866589	0.26	p_0: 0.999<br>p: 0.134<br>q: 0.371<br>p1: 0.00001<br>ω: 1.0	$P << 0.001$	0 ($PP \geq 0.99$)<br>0 ($P > 0.95$)

Legend:

a: dn/ds (weighted average)

b: Significance of the model in comparison with the null model

c: Number of sites with $\omega > 1$ under the Bayes empirical Bayes approach with a posterior probability (PP) more than or equal to 0.99 and 0.95

* Models which allow $\omega > 1$

Table S1.10 Maximum-likelihood parameter estimates for turtle nerve growth factors

Model	Likelihood (ι)	ω_0[a]	Parameters	Sign.[b]	No. of Sites with $\omega > 1$[c]
					**B.E.B**
**M0 (One ratio)**	-1348.106425	0.30	$= \omega_0$		-
**M1 (Neutral)**	-1340.646247	0.34	P_0: 0.734<br>ω_0: 0.11<br>P_1: 0.265<br>ω1: 1.0		-
**M2 (Selection)***	-1340.646217	0.34	P_0: 0.734<br>ω_0: 0.11<br>P_1: 0.183<br>ω_1:1.0<br>P_2: 0.081<br>ω_2: 1.0	P << 0.001	0 (PP ≥ 0.99)<br>0 (P > 0.95)
**M3 (Discrete)***	-1340.152735	0.31	P_0: 0.365<br>ω_0: 0.0<br>P_1: 0.323<br>ω_1: 0.24<br>P_2: 0.310<br>ω_2: 0.77	P << 0.001	-
**M7 (beta)**	-1340.175892	0.32	p: 0.31471<br>q: 0.66249		-
**M8 (beta and ω)***	-1340.175892	0.32	p_0: 1.0<br>p: 0.314<br>q: 0.662<br>p1: 0.0<br>ω: 1.0	P << 0.001	0 (PP ≥ 0.99)<br>0 (P > 0.95)

Legend:

a: dn/ds (weighted average)

b: Significance of the model in comparison with the null model

c: Number of sites with ω > 1 under the Bayes empirical Bayes approach with a posterior probability (PP) more than or equal to 0.99 and 0.95

* Models which allow ω > 1

Table S1.11 Maximum-likelihood parameter estimates for mammalian nerve growth factors

Model	Likelihood (ι)	ω_0[a]	Parameters	Sign.[b]	No. of Sites with $\omega > 1$[c]
					B.E.B
**M0 (One ratio)**	-2588.712520	0.09	$= \omega_0$		-
**M1 (Neutral)**	-2575.113733	0.17	P_0: 0.863<br>ω_0: 0.04<br>P_1: 0.136<br>ω1: 1.0		-
**M2 (Selection)***	-2575.113733	0.17	P_0: 0.863<br>ω_0: 0.04<br>P_1: 0.070<br>ω_1:1.0<br>P_2: 0.065<br>ω_2: 1.0	P << 0.001	0 (PP ≥ 0.99)<br>0 (P > 0.95)
**M3 (Discrete)***	-2552.967953	0.11	P_0: 0.475<br>ω_0: 0.007<br>P_1: 0.392<br>ω_1: 0.116<br>P_2: 0.132<br>ω_2: 0.491	P << 0.001	-
**M7 (beta)**	-2553.020726	0.11	p: 0.36046<br>q: 2.71196		-
**M8 (beta and ω)***	-2553.020729	0.11	p_0: 1.0<br>p: 0.360<br>q: 2.71<br>p1: 0.0<br>ω: 1.0	P << 0.001	0 (PP ≥ 0.99)<br>0 (P > 0.95)

Legend:

a: dn/ds (weighted average)

b: Significance of the model in comparison with the null model

c: Number of sites with ω > 1 under the Bayes empirical Bayes approach with a posterior probability (PP) more than or equal to 0.99 and 0.95

* Models which allow ω > 1
