# Supplementary material for: Molecular Evolution of Vertebrate Neurotrophins: Co-Option of the Highly Conserved Nerve Growth Factor Gene into the Advanced Snake Venom Arsenalf
Source: PLoS One. 2013 Nov 29;8(11):e81827. doi: 10.1371/journal.pone.0081827 (PMC3843689; doi:10.1371/journal.pone.0081827)
Supplement: Table S3 — (S3.1-S3.11) Details of selection analyses of neurotrophin-3 (NT3). a: dn/ds (weighted average). b: Significance of the model in comparison with the null model. c: Number of sites with ω > 1 under the Bayes empirical Bayes approach with a posterior probability (PP) more than or equal to 0.99 and 0.95. * Models which allow ω > 1. (PDF) [file pone.0081827.s003.pdf]

Table S3.1 Maximum-likelihood parameter estimates for Elapidae neurotrophin-3

Model	Likelihood (ι)	ω_0[a]	Parameters	Sign.[b]	No. of Sites with $\omega > 1$[c]
					**B.E.B**
**M0 (One ratio)**	-694.206382	0.49	$= \omega_0$		-
**M1 (Neutral)**	-673.832213	0.43	P_0: 0.578<br>ω0: 0.02<br>P_1: 0.421<br>ω1: 1.0		-
**M2 (Selection)***	-669.684563	0.92	P_0: 0.511<br>ω_0: 0.02<br>P_1: 0.366<br>ω_1:1.0<br>P_2: 0.121<br>ω_2:4.59	$P < 0.05$	0 ($PP \geq 0.99$)<br>1 ($P \geq 0.95$)
**M3 (Discrete)***	-669.242390	0.79	P_0: 0.471<br>ω_0: 0.0<br>P_1: 0.391<br>ω_1: 0.67<br>P_2: 0.137<br>ω_2: 3.84	$P << 0.001$	-
**M7 (beta)**	-673.779182	0.42	p: 0.02013<br>q: 0.02583		-
**M8 (beta and ω)***	-669.558752	0.84	p_0: 0.873<br>p: 0.082<br>q: 0.143<br>p1: 0.126<br>ω: 4.18	$P < 0.05$	1 ($PP \geq 0.99$)<br>3 ($P > 0.95$)

Legend:

a: dn/ds (weighted average)

b: Significance of the model in comparison with the null model

c: Number of sites with $\omega > 1$ under the Bayes empirical Bayes approach with a posterior probability (PP) more than or equal to 0.99 and 0.95

* Models which allow $\omega > 1$

Table S3.2 Maximum-likelihood parameter estimates for Viperidae neurotrophin-3

Model	Likelihood (ι)	ω_0[a]	Parameters	Sign.[b]	No. of Sites with ω > 1[c]
					**B.E.B**
**M0 (One ratio)**	-787.105320	0.31	$= \omega_0$		-
**M1 (Neutral)**	-768.396299	0.71	P_0: 0.289<br>ω0: 0.001<br>P_1: 0.710<br>ω1:1.0		-
**M2 (Selection)***	-768.396299	0.71	P_0: 0.289<br>ω_0: 0.001<br>P_1: 0.499<br>ω_1:1.0<br>P_2: 0.210<br>ω_2:1.0	$P > 0.05^{N.S}$	0 (PP ≥ 0.99)<br>0 (P ≥ 0.95)
**M3 (Discrete)***	-761.777562	0.41	P_0: 0.250<br>ω_0: 0.0<br>P_1: 0.264<br>ω_1: 0.10<br>P_2: 0.484<br>ω_2: 0.80	P << 0.001	-
**M7 (beta)**	-763.149655	0.44	p: 0.18348<br>q: 0.22677		-
**M8 (beta and ω)***	-763.149658	0.44	p_0: 0.999<br>p: 0.183<br>q: 0.226<br>p1: 0.00001<br>ω: 1.0	$P > 0.05^{N.S}$	0 (PP ≥ 0.99)<br>0 (P > 0.95)

Legend:

a: dn/ds (weighted average)

b: Significance of the model in comparison with the null model

c: Number of sites with ω > 1 under the Bayes empirical Bayes approach with a posterior probability (PP) more than or equal to 0.99 and 0.95

* Models which allow ω > 1

$P > 0.05^{N.S}$: Not significant at 0.05

Table S3.3 Maximum-likelihood parameter estimates for 'non-front-fanged' advanced snake neurotrophin-3

Model	Likelihood (ι)	ω_0[a]	Parameters	Sign.[b]	No. of Sites with ω > 1[c]
					**B.E.B**
**M0 (One ratio)**	-744.714302	1.88	= ω_0		-
**M1 (Neutral)**	-741.684732	0.70	P_0: 0.292<br>ω0: 0.0<br>P_1: 0.707<br>ω1: 1.0		-
**M2 (Selection)***	-731.444226	2.24	P_0: 0.239<br>ω_0: 0.0<br>P_1: 0.370<br>ω_1:1.0<br>P_2: 0.390<br>ω_2: 4.78	P << 0.001	3 (PP ≥ 0.99)<br>2 (P ≥ 0.95)
**M3 (Discrete)***	-729.662821	2.60	P_0: 0.343<br>ω_0: 0.0<br>P_1: 0.592<br>ω_1: 2.93<br>P_2: 0.064<br>ω_2: 13.44	P << 0.001	-
**M7 (beta)**	-741.689003	0.70	p: 0.01169<br>q: 0.00500		-
**M8 (beta and ω)***	-731.500666	2.25	p_0: 0.561<br>p: 0.005<br>q: 0.005<br>p1: 0.438<br>ω: 4.51	P << 0.001	4 (PP ≥ 0.99)<br>5 (P > 0.95)

Legend:

a: dn/ds (weighted average)

b: Significance of the model in comparison with the null model

c: Number of sites with ω > 1 under the Bayes empirical Bayes approach with a posterior probability (PP) more than or equal to 0.99 and 0.95

* Models which allow ω > 1

Table S3.4 Maximum-likelihood parameter estimates for Typhlopoidea neurotrophin-3

Model	Likelihood (ι)	ω_0[a]	Parameters	Sign.[b]	No. of Sites with ω > 1[c]
					**B.E.B**
**M0 (One ratio)**	-744.714302	1.88	$= \omega_0$		-
**M1 (Neutral)**	-741.684732	0.70	P_0: 0.292<br>ω0: 0.0<br>P_1: 0.707<br>ω1: 1.0		-
**M2 (Selection)***	-731.444226	2.24	P_0: 0.239<br>ω_0: 0.0<br>P_1: 0.370<br>ω_1:1.0<br>P_2: 0.390<br>ω_2: 4.78	P << 0.001	3 (PP ≥ 0.99)<br>2 (P ≥ 0.95)
**M3 (Discrete)***	-729.662821	2.60	P_0: 0.343<br>ω_0: 0.0<br>P_1: 0.592<br>ω_1: 2.93<br>P_2: 0.064<br>ω_2: 13.44	P << 0.001	-
**M7 (beta)**	-741.689003	0.70	p: 0.01169<br>q: 0.00500		-
**M8 (beta and ω)***	-731.500666	2.25	p_0: 0.561<br>p: 0.005<br>q: 0.005<br>p1: 0.438<br>ω: 4.51	P << 0.001	4 (PP ≥ 0.99)<br>5 (P > 0.95)

Legend:

a: dn/ds (weighted average)

b: Significance of the model in comparison with the null model

c: Number of sites with ω > 1 under the Bayes empirical Bayes approach with a posterior probability (PP) more than or equal to 0.99 and 0.95

* Models which allow ω > 1

Table S3.5 Maximum-likelihood parameter estimates for Boidae (Henophidia) neurotrophin-3

Model	Likelihood (ι)	ω_0[a]	Parameters	Sign.[b]	No. of Sites with ω > 1[c]
					**B.E.B**
**M0 (One ratio)**	-744.714302	1.88	$= \omega_0$		-
**M1 (Neutral)**	-741.684732	0.70	P_0: 0.292<br>ω0: 0.0<br>P_1: 0.707<br>ω1: 1.0		-
**M2 (Selection)***	-731.444226	2.24	P_0: 0.239<br>ω_0: 0.0<br>P_1: 0.370<br>ω_1:1.0<br>P_2: 0.390<br>ω_2: 4.78	P << 0.001	3 (PP ≥ 0.99)<br>2 (P ≥ 0.95)
**M3 (Discrete)***	-729.662821	2.60	P_0: 0.343<br>ω_0: 0.0<br>P_1: 0.592<br>ω_1: 2.93<br>P_2: 0.064<br>ω_2: 13.44	P << 0.001	-
**M7 (beta)**	-741.689003	0.70	p: 0.01169<br>q: 0.00500		-
**M8 (beta and ω)***	-731.500666	2.25	p_0: 0.561<br>p: 0.005<br>q: 0.005<br>p1: 0.438<br>ω: 4.51	P << 0.001	4 (PP ≥ 0.99)<br>5 (P > 0.95)

Legend:

a: dn/ds (weighted average)

b: Significance of the model in comparison with the null model

c: Number of sites with ω > 1 under the Bayes empirical Bayes approach with a posterior probability (PP) more than or equal to 0.99 and 0.95

* Models which allow ω > 1

Table S3.6 Maximum-likelihood parameter estimates for Scinciformata neurotrophin-3

Model	Likelihood (ι)	ω_0[a]	Parameters	Sign.[b]	No. of Sites with $\omega > 1$[c]
					**B.E.B**
**M0 (One ratio)**	-744.714302	1.88	$= \omega_0$		-
**M1 (Neutral)**	-741.684732	0.70	P_0: 0.292<br>ω0: 0.0<br>P_1: 0.707<br>ω1: 1.0		-
**M2 (Selection)***	-731.444226	2.24	P_0: 0.239<br>ω_0: 0.0<br>P_1: 0.370<br>ω_1:1.0<br>P_2: 0.390<br>ω_2: 4.78	$P << 0.001$	3 ($PP \geq 0.99$)<br>2 ($P \geq 0.95$)
**M3 (Discrete)***	-729.662821	2.60	P_0: 0.343<br>ω_0: 0.0<br>P_1: 0.592<br>ω_1: 2.93<br>P_2: 0.064<br>ω_2: 13.44	$P << 0.001$	-
**M7 (beta)**	-741.689003	0.70	p: 0.01169<br>q: 0.00500		-
**M8 (beta and ω)***	-731.500666	2.25	p_0: 0.561<br>p: 0.005<br>q: 0.005<br>p1: 0.438<br>ω: 4.51	$P << 0.001$	4 ($PP \geq 0.99$)<br>5 ($P > 0.95$)

Legend:

a: dn/ds (weighted average)

b: Significance of the model in comparison with the null model

c: Number of sites with $\omega > 1$ under the Bayes empirical Bayes approach with a posterior probability (PP) more than or equal to 0.99 and 0.95

* Models which allow $\omega > 1$

Table S3.7 Maximum-likelihood parameter estimates for Iguania neurotrophin-3

Model	Likelihood (ι)	ω_0[a]	Parameters	Sign.[b]	No. of Sites with $\omega > 1$[c]<br>B.E.B
**M0 (One ratio)**	-744.714302	1.88	$= \omega_0$		-
**M1 (Neutral)**	-741.684732	0.70	P_0: 0.292<br>ω0: 0.0<br>P_1: 0.707<br>ω1: 1.0		-
**M2 (Selection)***	-731.444226	2.24	P_0: 0.239<br>ω_0: 0.0<br>P_1: 0.370<br>ω_1:1.0<br>P_2: 0.390<br>ω_2: 4.78	$P \ll 0.001$	3 ($PP \geq 0.99$)<br>2 ($P \geq 0.95$)
**M3 (Discrete)***	-729.662821	2.60	P_0: 0.343<br>ω_0: 0.0<br>P_1: 0.592<br>ω_1: 2.93<br>P_2: 0.064<br>ω_2: 13.44	$P \ll 0.001$	-
**M7 (beta)**	-741.689003	0.70	p: 0.01169<br>q: 0.00500		-
**M8 (beta and ω)***	-731.500666	2.25	p_0: 0.561<br>p: 0.005<br>q: 0.005<br>p1: 0.438<br>ω: 4.51	$P \ll 0.001$	4 ($PP \geq 0.99$)<br>5 ($P > 0.95$)

Legend:

a: dn/ds (weighted average)

b: Significance of the model in comparison with the null model

c: Number of sites with $\omega > 1$ under the Bayes empirical Bayes approach with a posterior probability (PP) more than or equal to 0.99 and 0.95

* Models which allow $\omega > 1$

Table S3.8 Maximum-likelihood parameter estimates for Anguimorpha neurotrophin-3

Model	Likelihood (ι)	ω_0[a]	Parameters	Sign.[b]	No. of Sites with ω > 1[c]
					**B.E.B**
**M0 (One ratio)**	-744.714302	1.88	$= \omega_0$		-
**M1 (Neutral)**	-741.684732	0.70	P_0: 0.292<br>ω0: 0.0<br>P_1: 0.707<br>ω1: 1.0		-
**M2 (Selection)***	-731.444226	2.24	P_0: 0.239<br>ω_0: 0.0<br>P_1: 0.370<br>ω_1:1.0<br>P_2: 0.390<br>ω_2: 4.78	P << 0.001	3 (PP ≥ 0.99)<br>2 (P ≥ 0.95)
**M3 (Discrete)***	-729.662821	2.60	P_0: 0.343<br>ω_0: 0.0<br>P_1: 0.592<br>ω_1: 2.93<br>P_2: 0.064<br>ω_2: 13.44	P << 0.001	-
**M7 (beta)**	-741.689003	0.70	p: 0.01169<br>q: 0.00500		-
**M8 (beta and ω)***	-731.500666	2.25	p_0: 0.561<br>p: 0.005<br>q: 0.005<br>p1: 0.438<br>ω: 4.51	P << 0.001	4 (PP ≥ 0.99)<br>5 (P > 0.95)

Legend:

a: dn/ds (weighted average)

b: Significance of the model in comparison with the null model

c: Number of sites with ω > 1 under the Bayes empirical Bayes approach with a posterior probability (PP) more than or equal to 0.99 and 0.95

* Models which allow ω > 1

Table S3.9 Maximum-likelihood parameter estimates for crocodilian neurotrophin-3

Model	Likelihood (ι)	ω_0[a]	Parameters	Sign.[b]	No. of Sites with ω > 1[c]
					**B.E.B**
**M0 (One ratio)**	-744.714302	1.88	$= \omega_0$		-
**M1 (Neutral)**	-741.684732	0.70	P_0: 0.292<br>ω0: 0.0<br>P_1: 0.707<br>ω1: 1.0		-
**M2 (Selection)***	-731.444226	2.24	P_0: 0.239<br>ω_0: 0.0<br>P_1: 0.370<br>ω_1:1.0<br>P_2: 0.390<br>ω_2: 4.78	P << 0.001	3 (PP ≥ 0.99)<br>2 (P ≥ 0.95)
**M3 (Discrete)***	-729.662821	2.60	P_0: 0.343<br>ω_0: 0.0<br>P_1: 0.592<br>ω_1: 2.93<br>P_2: 0.064<br>ω_2: 13.44	P << 0.001	-
**M7 (beta)**	-741.689003	0.70	p: 0.01169<br>q: 0.00500		-
**M8 (beta and ω)***	-731.500666	2.25	p_0: 0.561<br>p: 0.005<br>q: 0.005<br>p1: 0.438<br>ω: 4.51	P << 0.001	4 (PP ≥ 0.99)<br>5 (P > 0.95)

Legend:

a: dn/ds (weighted average)

b: Significance of the model in comparison with the null model

c: Number of sites with ω > 1 under the Bayes empirical Bayes approach with a posterior probability (PP) more than or equal to 0.99 and 0.95

* Models which allow ω > 1

Table S3.10 Maximum-likelihood parameter estimates for turtle neurotrophin-3

Model	Likelihood (ι)	ω_0[a]	Parameters	Sign.[b]	No. of Sites with $\omega > 1$[c]
					**B.E.B**
**M0 (One ratio)**	-744.714302	1.88	$= \omega_0$		-
**M1 (Neutral)**	-741.684732	0.70	P_0: 0.292<br>ω0: 0.0<br>P_1: 0.707<br>ω1: 1.0		-
**M2 (Selection)***	-731.444226	2.24	P_0: 0.239<br>ω_0: 0.0<br>P_1: 0.370<br>ω_1:1.0<br>P_2: 0.390<br>ω_2: 4.78	$P << 0.001$	3 ($PP \geq 0.99$)<br>2 ($P \geq 0.95$)
**M3 (Discrete)***	-729.662821	2.60	P_0: 0.343<br>ω_0: 0.0<br>P_1: 0.592<br>ω_1: 2.93<br>P_2: 0.064<br>ω_2: 13.44	$P << 0.001$	-
**M7 (beta)**	-741.689003	0.70	p: 0.01169<br>q: 0.00500		-
**M8 (beta and ω)***	-731.500666	2.25	p_0: 0.561<br>p: 0.005<br>q: 0.005<br>p1: 0.438<br>ω: 4.51	$P << 0.001$	4 ($PP \geq 0.99$)<br>5 ($P > 0.95$)

Legend:

a: dn/ds (weighted average)

b: Significance of the model in comparison with the null model

c: Number of sites with $\omega > 1$ under the Bayes empirical Bayes approach with a posterior probability (PP) more than or equal to 0.99 and 0.95

* Models which allow $\omega > 1$

Table S3.11 Maximum-likelihood parameter estimates for mammalian neurotrophin-3

Model	Likelihood (ι)	ω_0[a]	Parameters	Sign.[b]	No. of Sites with $\omega > 1$[c]
					**B.E.B**
**M0 (One ratio)**	-744.714302	1.88	$= \omega_0$		-
**M1 (Neutral)**	-741.684732	0.70	P_0: 0.292<br>ω0: 0.0<br>P_1: 0.707<br>ω1: 1.0		-
**M2 (Selection)***	-731.444226	2.24	P_0: 0.239<br>ω_0: 0.0<br>P_1: 0.370<br>ω_1:1.0<br>P_2: 0.390<br>ω_2: 4.78	P << 0.001	3 (PP ≥ 0.99)<br>2 (P ≥ 0.95)
**M3 (Discrete)***	-729.662821	2.60	P_0: 0.343<br>ω_0: 0.0<br>P_1: 0.592<br>ω_1: 2.93<br>P_2: 0.064<br>ω_2: 13.44	P << 0.001	-
**M7 (beta)**	-741.689003	0.70	p: 0.01169<br>q: 0.00500		-
**M8 (beta and ω)***	-731.500666	2.25	p_0: 0.561<br>p: 0.005<br>q: 0.005<br>p1: 0.438<br>ω: 4.51	P << 0.001	4 (PP ≥ 0.99)<br>5 (P > 0.95)

Legend:

a: dn/ds (weighted average)

b: Significance of the model in comparison with the null model

c: Number of sites with $\omega > 1$ under the Bayes empirical Bayes approach with a posterior probability (PP) more than or equal to 0.99 and 0.95

* Models which allow $\omega > 1$
