# Supplementary material for: Molecular Evolution of Vertebrate Neurotrophins: Co-Option of the Highly Conserved Nerve Growth Factor Gene into the Advanced Snake Venom Arsenalf
Source: PLoS One. 2013 Nov 29;8(11):e81827. doi: 10.1371/journal.pone.0081827 (PMC3843689; doi:10.1371/journal.pone.0081827)
Supplement: Table S4 — Lineage-specific selection analyses of nerve growth factors (NGF). a: dn/ds (weighted average). b: Significance of the model in comparison with the null model. * Significant after Bonferroni correction. NS: Not significant. Significantly detected positively selected lineages are highlighted in bold. (PDF) [file pone.0081827.s004.pdf]

Table S4. Lineage-specific estimates for Toxicofera nerve growth factors (NGF)

Model	ω[a]	Likelihood (ι)	Prop. of sites with ω > 1	Sign[d]
<u>**Elapidae**</u>				
Two-ratio Model	0.85	-7902.275528	-	**p > 0.05[NS]**
**Branch-site Model A**	**3.90**	**-7731.163474**	**4.5%**	***p << 0.001**
**Clade Model C**	**2.38**	**-10800.007151**	**2.6%**	***p << 0.001**
<u>**Viperidae**</u>				
Two-ratio Model	0.82	-7909.191912	-	**p > 0.05[NS]**
**Branch-site Model A**	**2.01**	**-7786.640851**	**8.1%**	**p < 0.05**
**Clade Model C**	**1.03**	**-10800.007151**	**2.6%**	***p << 0.001**

Legend:

a: dn/ds (weighted average)

b: Significance of the model in comparison with the null model

* Significant after Bonferroni correction

NS: Not significant

Significantly detected positively selected lineages are highlighted in bold
