# Supplementary material for: Molecular Evolution of Vertebrate Neurotrophins: Co-Option of the Highly Conserved Nerve Growth Factor Gene into the Advanced Snake Venom Arsenalf
Source: PLoS One. 2013 Nov 29;8(11):e81827. doi: 10.1371/journal.pone.0081827 (PMC3843689; doi:10.1371/journal.pone.0081827)
Supplement: Table S5 — (S5.1-S5.3) Sequences analysed. (PDF) [file pone.0081827.s005.pdf]

Supplementary File S5.1 Nerve growth factor (NGF) sequence sets

	Elapidae	Accession
1	Bungarus multicinctus	S56212.1
2	Cacophis squamulosa	GC_RL8_rep_c1593
3	Demansia vestigiata	DQ917528.1
4	Demansia vestigiata	DQ917526.1
5	Demansia vestigiata	DQ917527.1
6	Drysdalia coronoides	HM627208.1
7	Echiopsis curta	Ecu_RL8_rep_c378
8	Echiopsis curta	Ecu_RL8_rep_c757
9	Furina ornata	For_RL9_rep_c360
10	Furina ornata	For_RL9_rep_c523
11	Hoplocephalus bungaroides	Hbu_RL1_rep_c521
12	Hoplocephalus bungaroides	Hbu_RL1_rep_c597
13	Hoplocephalus bungaroides	Hbu_RL1_rep_c786
14	Hoplocephalus bungaroides	Hbu_RL1_rep_c923
15	Hoplocephalus bungaroides	Hbu_RL1_rep_c961
16	Hoplocephalus stephensii	DQ181915.1
17	Hoplocephalus stephensii	DQ181916.1
18	Laticauda colubrina	EU438011.1
19	Micrurus fulvius	EU438017.1
20	Naja kaouthia	EU438018.1
21	Naja sputatrix	AY527216.1
22	Naja sputatrix	AY527215.1
23	Notechis scutatus	DQ181908.1
24	Notechis scutatus	DQ181910.1
25	Notechis scutatus	DQ181909.1
26	Notechis scutatus	EU438020.1
27	Oxyuranus microlepidotus	DQ181904.1
28	Oxyuranus microlepidotus	DQ181905.1
29	Oxyuranus scutellatus	DQ084064.1
30	Parasuta nigriceps	FJ790485.1
31	Pseudechis australis	DQ181912.1
32	Pseudechis australis	DQ181914.1
33	Pseudechis australis	DQ181913.1
34	Pseudechis porphyriacus	DQ181911.1
35	Pseudonaja modesta	Pmo_RL10_rep_c337
36	Pseudonaja textilis	DQ181907.1
37	Pseudonaja textilis	DQ181906.1
38	Rhinoplocephalus nigrescens	DQ422727.1
39	Simoselaps roperi	Bse_RL2_rep_c1017
40	Simoselaps roperi	Bse_RL2_rep_c1023
41	Simoselaps roperi	Bse_RL2_rep_c1058
42	Simoselaps roperi	Bse_RL2_rep_c1060
43	Simoselaps roperi	Bse_RL2_rep_c1086
44	Simoselaps roperi	Bse_RL2_rep_c1103
45	Simoselaps roperi	Bse_RL2_rep_c1131
46	Simoselaps roperi	Bse_RL2_rep_c1310
47	Simoselaps roperi	Bse_RL2_rep_c2194
48	Simoselaps roperi	Bse_RL2_rep_c3514
49	Simoselaps roperi	Bse_RL2_rep_c4864

	Viperidae	Accession
1	Agkistrodon contortrix	EU437986.1
2	Azemiops feae	DQ139929.1
3	Azemiops feae	Afe_RL4_rep_c388
4	Azemiops feae	EU437992.1
5	Bitis gabonica	AY430406.1
6	Bothrops asper	EU437994.1
7	Bothrops jararacussu	AY007318.1
8	Causus defilippi	EU437997.1
9	Crotalus adamanteus	JU173670.1
10	Crotalus durissus	AF306533.1
11	Crotalus o helleri	CohCI9_RL3_c37
12	Crotalus o helleri	CohID2_RL5_rep_c250
13	Crotalus o helleri	CohLL5_RL4_rep_c451
14	Crotalus o helleri	CohLL5_RL4_rep_c668
15	Crotalus o helleri	CohPH1_RL6_rep_c413
16	Daboia russellii	EU438000.1
17	Lachesis stenophrys	EU438008.1
18	Macrovipera lebetina	AY740013.1
19	Sistrurus catenatus edwardsi	DQ464261.1
20	Protobothrops flavoviridis	AB362782.1

	'Non-front-fanged'	Accession
1	Afronatrix anoscopus	EU437985.1
2	Amphiesma stolata	EU437987.1
3	Coluber constrictor	EU437998.1
4	Diadophis punctatus	EU438001.1
5	Heterodon platirhinos	EU438005.1
6	Imantodes cenchoa	EU438007.1
7	Lampropeltis getula	EU438009.1
8	Natrix natrix	EU438019.1
9	Sonora semiannulata	EU438023.1
10	Thamnophis marcianus	EU438024.1
11	Trimorphodon biscutatus	EU438026.1
12	Xenochrophis piscator	EU438030.1

	Henophidia	Accession
1	Boa constrictor	EU437993.1
2	Calabaria reinhardtii	EU437995.1
3	Casarea dussumieri	EU437996.1
4	Charina trivirgata	EU438013.1
5	Cylindrophis ruffus	EU437999.1
6	Epicrates striatus	EU438002.1
7	Eryx colubrinus	EU438003.1
8	Exiliboa placata	EU438004.1
9	Loxocemus bicolor	EU438015.1
10	Python molurus	EU437990.1
11	Python molurus	EU438022.1
12	Xenopeltis unicolor	EU438032.1

50	Simoselaps roperi	Bse_RL2_rep_c4921
51	Simoselaps roperi	Bse_RL2_rep_c895
52	Simoselaps roperi	Bse_RL2_rep_c972
53	Tropidechis carinatus	DQ181919.1
54	Tropidechis carinatus	DQ181918.1
55	Tropidechis carinatus	DQ181920.1
56	Tropidechis carinatus	DQ181921.1
57	Tropidechis carinatus	DQ181917.1
58	Vermicella annulata	Van_RL12_rep_c317
59	Vermicella annulata	Van_RL12_rep_c673
60	Denisonia devisii	Dde_RL7_rep_c182
61	Denisonia devisii	Dde_RL7_rep_c307

	**Iguania**	**Accession**
1	Acanthosaura lepidogaster	JF818319.1
2	Anolis carolinensis	EU437979.1
3	Basiliscus basiliscus	JF818336.1
4	Brachylophus fasciatus	JF818341.1
5	Chalarodon madagascariensis	JF818349.1
6	Chamaeleo calyptratus	GU432713.1
7	Chlamydosaurus kingii	JF818322.1
8	Corytophanes cristatus	JF818337.1
9	Crotaphytus collaris	JF818338.1
10	Ctenophorus isolepis	JF818323.1
11	Dipsosaurus dorsalis	JF818342.1
12	Draco blanfordii	JF818324.1
13	Enyalioides laticeps	GU432714.1
14	Gambelia wislizenii	JF818339.1
15	Hypsilurus boydii	JF818326.1
16	Leiocephalus barahonensis	JF818356.1
17	Leiolepis belliana	JF818335.1
18	Leiosaurus catamarcensis	JF818344.1
19	Liolaemus bellii	JF818347.1
20	Morunasaurus annularis	JF818340.1
21	Oplurus cyclurus	GU432716.1
22	Petrosaurus mearnsi	JF818350.1
23	Phrynocephalus mystaceus	JF818328.1
24	Phrynosoma platyrhinos	JF818351.1
25	Phymaturus palluma	JF818348.1
26	Physignathus lesueurii	JF818330.1
27	Pogona vitticeps	JF818331.1
28	Polychrus marmoratus	JF818355.1
29	Pristidactylus torquatus	JF818345.1
30	Saara hardwickii	GU432715.1
31	Sauromalus ater	JF818343.1
32	Sceloporus variabilis	JF818352.1
33	Stenocercus guentheri	JF818357.1
34	Tropidurus plica	JF818358.1
35	Uma scoparia	JF818353.1
36	Uranoscodon superciliosus	JF818359.1
37	Urostrophus vautieri	JF818346.1
38	Uta stansburiana	JF818354.1

	**Gekkota**	**Accession**
1	Coleonyx variegatus	JF818314.1
2	Eublepharis macularius	GU432729.1
3	Gekko gecko	EU437977.1
4	Gonatodes albogularis	GU432731.1
5	Hemidactylus brookii	HM180146.1
6	Hemidactylus coalescens	HM180179.1
7	Hemidactylus coalescens	HM180177.1
8	Hemidactylus coalescens	HM180143.1
9	Hemidactylus coalescens	HM180180.1
10	Hemidactylus coalescens	HM180178.1
11	Hemidactylus coalescens	HM180176.1
12	Hemidactylus eniangii	HM180175.1
13	Hemidactylus eniangii	HM180173.1
14	Hemidactylus eniangii	HM180141.1
15	Hemidactylus eniangii	HM180139.1
16	Hemidactylus eniangii	HM180137.1
17	Hemidactylus eniangii	HM180174.1
18	Hemidactylus eniangii	HM180142.1
19	Hemidactylus eniangii	HM180140.1
20	Hemidactylus fasciatus	HM180159.1
21	Hemidactylus fasciatus	HM180157.1
22	Hemidactylus fasciatus	HM180155.1
23	Hemidactylus fasciatus	HM180153.1
24	Hemidactylus fasciatus	HM180151.1
25	Hemidactylus fasciatus	HM180149.1
26	Hemidactylus fasciatus	HM180145.1
27	Hemidactylus fasciatus	HM180158.1
28	Hemidactylus fasciatus	HM180156.1
29	Hemidactylus fasciatus	HM180154.1
30	Hemidactylus fasciatus	HM180152.1
31	Hemidactylus fasciatus	HM180144.1
32	Hemidactylus kyaboboensis	HM180171.1
33	Hemidactylus kyaboboensis	HM180169.1
34	Hemidactylus kyaboboensis	HM180167.1
35	Hemidactylus kyaboboensis	HM180165.1
36	Hemidactylus kyaboboensis	HM180163.1
37	Hemidactylus kyaboboensis	HM180161.1
38	Hemidactylus kyaboboensis	HM180172.1
39	Hemidactylus kyaboboensis	HM180170.1

40	Hemidactylus kyaboboensis	HM180168.1
41	Hemidactylus kyaboboensis	HM180166.1
42	Hemidactylus kyaboboensis	HM180164.1
43	Hemidactylus kyaboboensis	HM180162.1
44	Hemidactylus kyaboboensis	HM180160.1
45	Hemidactylus kyaboboensis	HM180150.1
46	Hemidactylus kyaboboensis	HM180148.1
47	Saltuarius cornutus	JF818315.1
48	Strophurus ciliaris	GU432730.1

	**Laterata**	**Accession**
1	Amphisbaena alba	GU432741.1
2	Aspidoscelis tigris	EU437982.1
3	Bipes canaliculatus	GU432742.1
4	Lacerta viridis	GU432740.1
5	Pholidobolus macbrydei	GU432739.1
6	Rhineura floridana	GU432743.1
7	Trogonophis wiegmanni	GU432744.1

	**Scinciformata**	**Accession**
1	Acontias meleagris	GU432735.1
2	Cordylosaurus subtesselatus	JF818316.1
3	Cordylus mossambicus	GU432734.1
4	Feylinia polylepis	GU432736.1
5	Lepidophyma flavimaculatum	GU432738.1
6	Scincus scincus	GU432737.1
7	Xantusia vigilis	EU437983.1

	**Anguimorpha**	**Accession**
1	Anniella pulchra	GU432718.1
2	Elgaria multicarinata	GU432720.1
3	Heloderma horridum	GU432721.1
4	Heloderma suspectum	GU432722.1
5	Heloderma suspectum	EU790966.1
6	Lanthanotus borneensis	GU432725.1
7	Pseudopus apodus	GU432717.1
8	Shinisaurus crocodilurus	GU432723.1
9	Varanus exanthematicus	GU432727.1
10	Varanus salvator	EU437981.1
11	Xenosaurus grandis	GU432724.1
12	Xenosaurus platyceps	EU437980.1
13	Varanus acanthurus	GU432726.1
14	Celestus enneagrammus	GU432719.1
15	Varanus acanthurus	DQ139928.1
16	Abronia gramminea	Abro_RL8_rep_c474
17	Abronia gramminea	Abro_RL8_rep_c785
18	Heloderma suspectum	Hsu_RL11_rep_c662

	**Turtles**	**Accession**
1	Actinemys marmorata	GU085534.1
2	Actinemys marmorata	GQ896037.1
3	Batagur trivittata	GU085527.1
4	Carettochelys insculpta	GU085528.1
5	Chelonia mydas	GU085529.1
6	Chelydra serpentina	GU085531.1
7	Chrysemys picta	GQ896038.1
8	Clemmys guttata	GQ896039.1
9	Deirochelys reticularia	GQ896040.1
10	Dermochelys coriacea	GU085532.1
11	Dogania subplana	GU085533.1
12	Emydoidea blandingii	GQ896041.1

	**Mammals**	**Accession**
1	Bos taurus	NM_001099362.1
2	Bos taurus	Y09566.1
3	Canis familiaris	AY800386.1
4	Canis lupus familiaris	NM_001194950.1
5	Gorilla gorilla	AY091926.1
6	Gorilla gorilla	AB037519.1
7	Homo sapiens	AB489186.1
8	Homo sapiens	NG_007944.1
9	Homo sapiens	NM_002506.2
10	Homo sapiens	HQ258125.1
11	Homo sapiens	BT019733.1
12	Homo sapiens	AF411526.1
13	Homo sapiens	AF150960.1
14	Homo sapiens	AL049825.18
15	Homo sapiens	X52599.1
16	Homo sapiens	BC126150.1
17	Homo sapiens	BC126148.1
18	Homo sapiens	V01511.1
19	Homo sapiens	CR541855.1
20	Homo sapiens	X53655.1
21	Macaca fuscata	AF222682.1
22	Macaca sp.	AY091928.1
23	Mus musculus	NM_001112698.1
24	Mus musculus	NM_013609.2
25	Mus musculus	AK144588.1
26	Mus musculus	BC156604.1
27	Mus musculus	K01759.1
28	Mus musculus	V00836.1
29	Mus sp	M14805.1
30	Mus sp	M35075.1
31	Pan troglodytes	NM_001012437.1
32	Pan troglodytes	AY091925.1
33	Pongo pygmaeus	AY091927.1
34	Pongo pygmaeus	AB037520.1
35	Praomys natalensis	M22748.1
36	Rattus norvegicus	M36589.1
37	Saimiri boliviensis	AY665218.1
38	Sus scrofa	L31898.1

13	Emys orbicularis	GQ896042.1
14	Glyptemys insculpta	GQ896043.1
15	Glyptemys muhlenbergii	GQ896044.1
16	Graptemys barbouri	GQ896045.1
17	Graptemys caglei	GQ896046.1
18	Graptemys flavimaculata	GQ896047.1
19	Graptemys geographica	GQ896048.1
20	Graptemys gibbonsi	GQ896049.1
21	Graptemys nigrinoda	GQ896050.1
22	Graptemys ouachitensis	GQ896052.1
23	Graptemys ouachitensis	GQ896051.1
24	Graptemys pseudogeographica	GQ896053.1
25	Graptemys pulchra	GQ896054.1
26	Graptemys versa	GQ896055.1
27	Kinosternon flavescens	GU085535.1
28	Macrochelys temminckii	GU085538.1
29	Malaclemys terrapin	GQ896056.1
30	Malaclemys terrapin	GQ896057.1
31	Manouria emys	GU085536.1
32	Mauremys reevesii	GU085537.1
33	Pelodiscus sinensis	GU085541.1
34	Pseudemys concinna	GQ896058.1
35	Pseudemys gorzugi	GQ896059.1
36	Pseudemys nelsoni	GQ896060.1
37	Pseudemys peninsularis	GQ896061.1
38	Pseudemys rubriventris	GQ896062.1
39	Pseudemys texana	GQ896063.1
40	Rhinoclemmys annulata	GU085543.1
41	Sternotherus odoratus	GU085544.1
42	Terrapene carolina	GQ896065.1
43	Terrapene coahuila	GQ896066.1
44	Testudo graeca	GU085545.1
45	Trachemys scripta	GQ896072.1
46	Trachemys scripta	GQ896070.1
47	Trachemys scripta	GQ896071.1

Note: Unpublished sequences are available in supplementary file 2

Supplementary File S5.2 Brain-derived neurotrophic factor (BDNF) sequence sets

	Elapidae	Accession		Viperidae	Accession
1	Bungarus fasciatus	FJ433989.1	1	Agkistrodon contortrix	EU402623.1
2	Dendroaspis angusticeps	FJ433988.1	2	Azemiops feae	EU402628.1
3	Elapsoidea semiannulata	FJ433987.1	3	Bothriechis schlegelii	FJ433983.1
4	Laticauda colubrina	EU402647.1	4	Bothrops asper	EU402630.1
5	Laticauda colubrina	FJ433990.1	5	Bothrops atrox	DQ469794.1
6	Micrurus fulvius	EU402653.1	6	Causus defilippi	EU402633.1
7	Micrurus surinamensis	FJ433991.1	7	Daboia russellii	EU402636.1
8	Naja kaouthia	EU402654.1	8	Lachesis stenophrys	EU402644.1
9	Notechis scutatus	EU402656.1			

	'Non-front-fanged'	Accession		Typhlopidae	Accession
1	Amphiesma stolata	EU402624.1	1	Acutotyphlops sp.	GU902459.1
2	Calamaria pavimentata	FJ434005.1	2	Acutotyphlops subocularis	GU902418.1
3	Contia longicaudae	GU112371.1	3	Afrotyphlops fornasinii	GU902447.1
4	Contia longicaudae	GU112369.1	4	Afrotyphlops lineolatus	GU902451.1
5	Contia longicaudae	GU112367.1	5	Ramphotyphlops endoterus	GU902399.1
6	Contia longicaudae	GU112365.1	6	Ramphotyphlops ganei	GU902412.1
7	Contia longicaudae	GU112372.1	7	Ramphotyphlops guentheri	GU902404.1
8	Contia longicaudae	GU112368.1	8	Ramphotyphlops howi	GU902414.1
9	Contia longicaudae	GU112366.1	9	Ramphotyphlops kimberleyensis	GU902406.1
10	Contia longicaudae	GU112364.1	10	Ramphotyphlops splendidus	GU902416.1
11	Contia tenuis	GU112363.1	11	Letheobia unitaeniata	GU902452.1
12	Contia tenuis	GU112361.1	12	Megatyphlops schlegelii	GU902449.1
13	Contia tenuis	GU112359.1	13	Ramphotyphlops albiceps	GU902382.1
14	Contia tenuis	GU112357.1	14	Ramphotyphlops braminus	FJ433959.1
15	Contia tenuis	GU112355.1	15	Ramphotyphlops lineatus	GU902384.1
16	Contia tenuis	GU112353.1	16	Ramphotyphlops longissimus	GU902408.1
17	Contia tenuis	GU112351.1	17	Ramphotyphlops pilbarensis	GU902400.1
18	Contia tenuis	GU112349.1	18	Ramphotyphlops sp.	GU902420.1
19	Contia tenuis	GU112347.1	19	Ramphotyphlops waitii	GU902402.1
20	Contia tenuis	GU112345.1	20	Rhinotyphlops lalandei	GU902386.1
21	Contia tenuis	GU112343.1	21	Rhinotyphlops newtoni	GU902388.1
22	Contia tenuis	GU112341.1	22	Typhlops agoralionis	GU902422.1
23	Contia tenuis	GU112339.1	23	Typhlops andasibensis	GU902453.1
24	Contia tenuis	GU112337.1	24	Typhlops arator	GU902424.1
25	Contia tenuis	GU112360.1	25	Typhlops catapontus	GU902426.1
26	Contia tenuis	GU112358.1	26	Typhlops contorhinus	GU902446.1
27	Contia tenuis	GU112356.1	27	Typhlops dominicanus	GU902428.1
28	Contia tenuis	GU112354.1	28	Typhlops granti	GU902430.1
29	Contia tenuis	GU112350.1	29	Typhlops hedraeus	GU902392.1
30	Contia tenuis	GU112348.1	30	Typhlops jamaicensis	EU402664.1
31	Contia tenuis	GU112346.1	31	Typhlops jamaicensis	GU902432.1
32	Contia tenuis	GU112342.1	32	Typhlops lumbricalis	FJ433958.1
33	Contia tenuis	GU112340.1	33	Typhlops mirus	GU902394.1
34	Contia tenuis	GU112338.1	34	Typhlops monastus	GU902434.1
35	Cyclophiops major	AF497715.1	35	Typhlops notorachius	GU902436.1
36	Diadophis punctatus	FJ433997.1	36	Typhlops pammeces	GU902458.1
37	Diadophis punctatus	EU402637.1	37	Typhlops reticulatus	GU902396.1
38	Diadophis punctatus	GU112373.1	38	Typhlops richardi	GU902438.1

39	Grayia ornata	FJ434002.1
40	Hapsidophrys smaragdina	FJ434003.1
41	Heterodon platirhinos	GU112374.1
42	Imantodes cenchoa	EU402643.1
43	Lampropeltis getula	EU402645.1
44	Liophis lineatus	DQ469795.1
45	Natrix natrix	EU402655.1
46	Phyllorhynchus decurtatus	FJ434004.1
47	Pseudoxenodon bambusicola	FJ434000.1
48	Sonora semiannulata	EU402659.1
49	Thamnophis marcianus	EU402660.1
50	Trimorphodon biscutatus	EU402662.1
51	Xenochrophis flavipunctatus	FJ434001.1
52	Xenochrophis piscator	EU402666.1

	**Boidae**	**Accession**
1	Acrantophis dumerili	AY988032.1
2	Acrantophis madagascariensis	JQ073071.1
3	Acrantophis madagascariensis	FJ433973.1
4	Boa constrictor	FJ433975.1
5	Boa constrictor	EU402629.1
6	Calabaria reinhardtii	FJ433972.1
7	Calabaria reinhardtii	EU402631.1
8	Candoia carinata	FJ433974.1
9	Charina bottae	AY988042.1
10	Charina bottae BDNF	FJ433978.1
11	Charina trivirgata	FJ433979.1
12	Charina trivirgata	EU402649.1
13	Corallus caninus	JQ073072.1
14	Eryx colubrinus	EU402639.1
15	Eryx conicus	AY988040.1
16	Eryx miliaris	FJ433977.1
17	Gongylophis colubrinus	FJ433976.1
18	Sanzinia madagascariensis	JQ073070.1

	**Iguania**	**Accession**
1	Anolis acutus	EU544058.1
2	Anolis cooki	EU544066.1
3	Anolis cristatellus	EU544074.1
4	Anolis cristatellus	EU544072.1
5	Anolis cristatellus	EU544070.1
6	Anolis cristatellus	EU544073.1
7	Anolis desechensis	EU544068.1
8	Anolis distichus	EU544057.1
9	Anolis ernestwilliamsi	EU544069.1
10	Anolis gundlachi	EU544060.1
11	Anolis krugi	EU544061.1
12	Anolis monensis	EU544067.1
13	Anolis poncensis	EU544062.1
14	Anolis pulchellus	EU544063.1
15	Anolis scriptus	EU544065.1

39	Typhlops schwartzi	GU902440.1
40	Typhlops sp.	GU902460.1
41	Typhlops sp.	GU902454.1
42	Typhlops sylleptor	GU902442.1
43	Xenotyphlops grandidieri	GU902457.1
44	Xenotyphlops sp.	GU902456.1

	**Pythonidae**	**Accession**
1	Apodora papuana	FJ433971.1
2	Aspidites melanocephala	DQ465559.1
3	Aspidites melanocephalus	EU402627.1
4	Liasis savuensis	FJ433970.1
5	Morelia spilota	AY988035.1
6	Python molurus	EU402658.1
7	Python reticulatus	FJ433969.1

	**Anguimorpha**	**Accession**
1	Anniella geronimensis	EU445907.1
2	Anniella geronimensis	EU445908.1
3	Anniella geronimensis	EU445909.1
4	Anniella pulchra	EU445844.1
5	Anniella pulchra	EU445845.1
6	Anniella pulchra	EU445846.1
7	Anniella pulchra	EU445847.1
8	Anniella pulchra	EU445848.1
9	Anniella pulchra	EU445849.1
10	Anniella pulchra	EU445850.1
11	Anniella pulchra	EU445851.1
12	Anniella pulchra	EU445852.1
13	Anniella pulchra	EU445858.1
14	Anniella pulchra	EU445859.1
15	Anniella pulchra	EU445860.1
16	Anniella pulchra	EU445861.1
17	Anniella pulchra	EU445862.1
18	Anniella pulchra	EU445863.1
19	Anniella pulchra	EU445864.1
20	Anniella pulchra	EU445865.1
21	Anniella pulchra	EU445866.1
22	Anniella pulchra	EU445867.1
23	Anniella pulchra	EU445868.1
24	Anniella pulchra	EU445869.1
25	Anniella pulchra	EU445870.1
26	Anniella pulchra	EU445871.1
27	Anniella pulchra	EU445872.1
28	Anniella pulchra	EU445873.1
29	Anniella pulchra	EU445874.1
30	Anniella pulchra	EU445875.1
31	Anniella pulchra	EU445876.1
32	Anniella pulchra	EU445877.1
33	Anniella pulchra	EU445878.1
34	Anniella pulchra	EU445879.1
35	Anniella pulchra	EU445880.1

16	Anolis stratulus	EU544064.1
17	Basiliscus basiliscus	JF806017.1
18	Brachylophus fasciatus	JF806018.1
19	Chalarodon madagascariensis	JF806019.1
20	Corytophanes cristatus	JF806020.1
21	Crotaphytus collaris	JF806021.1
22	Enyalioides heterolepis	EU586759.1
23	Enyalioides heterolepis	EU586768.1
24	Enyalioides heterolepis	EU586760.1
25	Enyalioides laticeps	EU586761.1
26	Enyalioides laticeps	EU586762.1
27	Enyalioides microlepis	EU586763.1
28	Enyalioides oshaughnessyi	EU586764.1
29	Enyalioides palpebralis	EU586765.1
30	Enyalioides praestabilis	EU586766.1
31	Enyalioides sp.	EU586767.1
32	Hoplocercus spinosus	EU586769.1
33	Leiocephalus barahonensis	HQ876223.1
34	Leiosaurus catamarcensis	JF806022.1
35	Liolaemus bellii	HQ876220.1
36	Morunasaurus annularis	HQ876218.1
37	Petrosaurus mearnsi	JN648396.1
38	Petrosaurus mearnsi	HQ876221.1
39	Petrosaurus repens	JN648397.1
40	Phrynosoma coronatum	JN648400.1
41	Phrynosoma platyrhinos	JF806023.1
42	Phymaturus palluma	JF806024.1
43	Polychrus marmoratus	HQ876222.1
44	Pristidactylus	JF806025.1
45	Sauromalus ater	JF806026.1
46	Sceloporus consobrinus	GQ494863.1
47	Sceloporus consobrinus	GQ494861.1
48	Sceloporus consobrinus	GQ494856.1
49	Sceloporus cowlesi	GQ494859.1
50	Sceloporus cowlesi	GQ494857.1
51	Sceloporus hunsakeri	JN648399.1
52	Sceloporus tristichus	GQ494855.1
53	Sceloporus tristichus	GQ494853.1
54	Sceloporus undulatus	GQ494867.1
55	Sceloporus undulatus	GQ494865.1
56	Sceloporus undulatus	GQ494866.1
57	Sceloporus variabilis	JF806027.1
58	Stenocercus guentheri	HQ876224.1
59	Tropidurus plica	JF806028.1
60	Uma scoparia brain-derived	JF806029.1
61	Uranoscodon superciliosus	JF806030.1
62	Urosaurus bicarinatus	JN648384.1
63	Urosaurus clarionensis	JN648382.1
64	Urosaurus gadovi	JN648391.1
65	Urosaurus graciosus	JN648393.1
66	Urosaurus graciosus	JN648392.1
67	Urosaurus lahtelai	JN648385.1

36	Anniella pulchra	EU445881.1
37	Anniella pulchra	EU445882.1
38	Anniella pulchra	EU445883.1
39	Anniella pulchra	EU445884.1
40	Anniella pulchra	EU445885.1
41	Anniella pulchra	EU445886.1
42	Anniella pulchra	EU445887.1
43	Anniella pulchra	EU445888.1
44	Anniella pulchra	EU445889.1
45	Anniella pulchra	EU445890.1
46	Anniella pulchra	EU445891.1
47	Anniella pulchra	EU445892.1
48	Anniella pulchra	EU445893.1
49	Anniella pulchra	EU445894.1
50	Anniella pulchra	EU445895.1
51	Anniella pulchra	EU445896.1
52	Anniella pulchra	EU445897.1
53	Anniella pulchra	EU445898.1
54	Anniella pulchra	EU445899.1
55	Anniella pulchra	EU445900.1
56	Anniella pulchra	EU445901.1
57	Anniella pulchra	EU445902.1
58	Anniella pulchra	EU445903.1
59	Anniella pulchra	EU445904.1
60	Anniella pulchra	EU445905.1
61	Anniella pulchra	EU445906.1
62	Anniella pulchra	HM160579.1
63	Celestus enneagrammus	GU457853.1
64	Elgaria multicarinata	GU457854.1
65	Heloderma horridum	GU457855.1
66	Heloderma suspectum	FJ433955.1
67	Heloderma suspectum	GU457856.1
68	Lanthanotus borneensis	GU457859.1
69	Pseudopus apodus	GU457851.1
70	Shinisaurus crocodilurus	GU457857.1
71	Varanus acanthurus	GU457860.1
72	Varanus exanthematicus	GU457861.1
73	Varanus salvator voucher	EU402618.1
74	Xenosaurus grandis	GU457858.1
75	Xenosaurus platyceps	EU402617.1

	**Crocodiles**	**Accession**
1	Alligator mississippiensis	AF416631.1
2	Alligator mississippiensis	EU275888.1
3	Alligator mississippiensis	EU737934.1
4	Alligator mississippiensis	JN654788.1
5	Alligator sinensis	EF646303.1
6	Caiman crocodilus	EF646297.1
7	Caiman latirostris	EF646298.1
8	Caiman yacare	EF646299.1
9	Crocodylus acutus	EF646305.1
10	Crocodylus intermedius	EF646306.1

68	Urosaurus microscutatus	JN648386.1
69	Urosaurus ornatus	JN648389.1
70	Urosaurus ornatus	JN648390.1
71	Urostrophus vautieri	HQ876219.1
72	Uta squamata	JN648394.1
73	Uta stansburiana	JN648395.1
74	Uta stansburiana	JF806031.1

	**Turtles**	**Accession**
1	Batagur trivittata	GU085546.1
2	Caretta caretta	FJ009029.1
3	Caretta caretta	FJ009022.1
4	Carettochelys insculpta	GU085547.1
5	Chelonia mydas	GU085548.1
6	Chelonia mydas	FJ039950.1
7	Chelonia mydas	FJ039936.1
8	Chelonia mydas	FJ039943.1
9	Chelonia mydas	FJ039929.1
10	Chelonia mydas	FJ039922.1
11	Chelydra serpentina	GU085550.1
12	Chelydra serpentina	FJ230854.1
13	Chinemys reevesi	EF646319.1
14	Chrysemys picta	GU085549.1
15	Dermochelys coriacea	GU085551.1
16	Dermochelys coriacea	FJ039915.1
17	Dermochelys coriacea	FJ039908.1
18	Dogania subplana	GU085552.1
19	Eretmochelys imbricata	FJ039965.1
20	Eretmochelys imbricata	FJ039972.1
21	Erymnochelys madagascariensis	AY988078.1
22	Hydromedusa tectifera	AY988083.1
23	Kinosternon flavescens	GU085554.1
24	Lepidochelys kempii	FJ039993.1
25	Lepidochelys olivacea	FJ039979.1
26	Lepidochelys olivacea	FJ039986.1
27	Macrochelys temminckii	GU085557.1
28	Macrochelys temminckii	FJ230861.1
29	Manouria emys	GU085555.1
30	Mauremys reevesii	GU085556.1
31	Natator depressa	FJ039957.1
32	Pelodiscus sinensis	GU085560.1
33	Pelodiscus sinensis	FJ230868.1
34	Pelomedusa subrufa	GU085561.1
35	Pelomedusa subrufa	FJ230875.1
36	Pelomedusa subrufa	AY988081.1
37	Peltocephalus dumeriliana	AY988080.1
38	Pelusios gabonensis	AY988082.1
39	Platysternon megacephalum	GU085558.1
40	Psammobates pardalis	GU085559.1
41	Staurotypus triporcatus	AY988084.1
42	Sternotherus odoratus	GU085563.1
43	Testudo graeca	GU085564.1

11	Crocodylus johnsoni	EF646309.1
12	Crocodylus mindorensis	EF646310.1
13	Crocodylus moreletii	EF646307.1
14	Crocodylus niloticus	EF646314.1
15	Crocodylus niloticus	JQ073126.1
16	Crocodylus niloticus	JQ073127.1
17	Crocodylus novaeguineae	EF646311.1
18	Crocodylus palustris	EF646313.1
19	Crocodylus porosus	EF646312.1
20	Crocodylus porosus	JN654790.1
21	Crocodylus rhombifer	EF646304.1
22	Crocodylus siamensis	EF646308.1
23	Gavialis gangeticus	EF646318.1
24	Gavialis gangeticus	EU737935.1
25	Mecistops cataphractus	EF646315.1
26	Melanosuchus niger	EF646300.1
27	Osteolaemus tetraspis	EF646316.1
28	Paleosuchus palpebrosus	EF646301.1
29	Paleosuchus trigonatus	EF646302.1
30	Tomistoma schlegelii	EF646317.1

	**Mammals**	**Accession**
1	Ailuropoda melanoleuca	U56638.1
2	Ailurus fulgens	U56639.1
3	Bos taurus	NM_001046607.1
4	Bos taurus	X97914.1
5	Canis lupus	NM_001002975.1
6	Cavia porcellus	AB012097.1
7	Equus caballus	NM_001081787.1
8	Felis catus	NM_001009828.1
9	Helarctos malayanus	AF002240.1
10	Homo sapiens	AF400438.1
11	Homo sapiens	AY656701.1
12	Homo sapiens	M37762.1
13	Homo sapiens	X60201.1
14	Homo sapiens	M61176.1
15	Homo sapiens	BC029795.1
16	Homo sapiens	NM_001143808.1
17	Homo sapiens	AY054393.2
18	Homo sapiens	NM_001143805.1
19	Homo sapiens	AY054396.2
20	Homo sapiens	NM_001143809.1
21	Homo sapiens	AY054399.2
22	Homo sapiens	NM_001143812.1
23	Homo sapiens	EF689013.1
24	Homo sapiens	EF689017.1
25	Homo sapiens	AY054397.2
26	Homo sapiens	NM_001143807.1
27	Homo sapiens	AY054400.2
28	Homo sapiens	NM_170734.3
29	Homo sapiens	NM_001143813.1
30	Homo sapiens	AY054394.2

44	Trachemys scripta elegans	HM055376.1
45	Trachemys scripta elegans	HM055374.1
46	Trachemys scripta elegans	HM055372.1
47	Trachemys scripta elegans	HM055375.1
48	Trachemys scripta elegans	HM055373.1
49	Trionyx triunguis	HQ012595.1
50	Trionyx triunguis	HQ012593.1
51	Trionyx triunguis	HQ012596.1
52	Trionyx triunguis	HQ012594.1
53	Trionyx triunguis	HQ012592.1

31	Homo sapiens	NM_170733.3
32	Homo sapiens	NM_001143806.1
33	Homo sapiens	NM_001143810.1
34	Homo sapiens	NM_170732.4
35	Homo sapiens	NM_001709.4
36	Homo sapiens	NM_001143811.1
37	Homo sapiens	AY054392.2
38	Homo sapiens	NM_170731.4
39	Homo sapiens	NM_001143816.1
40	Homo sapiens	EF689010.1
41	Homo sapiens	NM_170735.5
42	Homo sapiens	EF689009.1
43	Lipotes vexillifer	AY700119.1
44	Mesocricetus auratus	FJ645258.1
45	Mus musculus	AF459642.1
46	Mus musculus	X55573.1
47	Mus musculus	BC034862.1
48	Mus musculus	EF125682.1
49	Mus musculus	AY231132.1
50	Mus musculus	AY057912.1
51	Pan troglodytes	NM_001012441.1
52	Procyon lotor	AF003188.1
53	Rattus norvegicus	AY176065.1
54	Rattus norvegicus	M61175.1
55	Rattus norvegicus	M61178.1
56	Rattus norvegicus	BC087634.1
57	Rattus norvegicus	X67108.1
58	Rattus norvegicus	EF125687.1
59	Rattus norvegicus	EF125688.1
60	Selenarctos thibetanus	DQ093584.1
61	Spermophilus citellus	AY646114.1
62	Sus scrofa	NM_214259.1
63	Ursus arctos	AF002239.1

Supplementary File S5.3 Neurotrophin-3 (NT-3) sequence sets

	Elapidae	Accession
1	Bungarus fasciatus	FJ434090.1
2	Dendroaspis angusticeps	FJ434089.1
3	Elapsoidea semiannulata	FJ434088.1
4	Laticauda colubrina	EU390927.1
5	Laticauda colubrina	FJ434091.1
6	Micrurus fulvius	EU390929.1
7	Micrurus surinamensis	FJ434092.1
8	Naja kaouthia	EU390930.1
9	Notechis scutatus	EU390932.1

	Viperidae	Accession
1	Agkistrodon contortrix	JN703027.1
2	Azemiops feae	EU390909.1
3	Bothriechis schlegelii	FJ434084.1
4	Bothrops asper	EU390910.1
5	Bothrops atrox	DQ469792.1
6	Causus defilippi	EU390913.1
7	Crotalus tigris	GQ334665.1
8	Daboia russellii	EU390916.1
9	Lachesis muta	EU390924.1
10	Sistrurus catenatus	GQ334686.1

	'Non-front-fanged'	Accession
1	Alsophis cantherigerus	FJ434100.1
2	Amastridium sapperi	GQ334663.1
3	Atractus elaps	GU353273.1
4	Atractus wagleri	GQ334664.1
5	Calamaria pavimentata	FJ434106.1
6	Coluber constrictor	EU390914.1
7	Cryophis hallbergi	GQ334666.1
8	Cryophis hallbergi	GU353276.1
9	Diadophis punctatus	EU390917.1
10	Diadophis punctatus	FJ434098.1
11	Diadophis punctatus	GU353272.1
12	Dipsas catesbyi	GU353277.1
13	Dipsas pratti	GQ334667.1
14	Grayia ornata	FJ434103.1
15	Hapsidophrys smaragdina	FJ434104.1
16	Heterodon platirhinos	EU390921.1
17	Heterodon platirhinos	GU353271.1
18	Homalopsis buccata	EU390922.1
19	Homalopsis buccata	FJ434087.1
20	Hypsiglena affinis	GU353278.1
21	Hypsiglena chlorophaea	FJ455195.1
22	Hypsiglena chlorophaea	FJ455198.1
23	Hypsiglena jani	FJ455193.1
24	Hypsiglena ochrorhyncha	FJ455199.1
25	Hypsiglena ochrorhyncha	FJ455200.1
26	Hypsiglena ochrorhyncha	FJ455201.1
27	Hypsiglena ochrorhyncha	FJ455202.1
28	Hypsiglena slevini	FJ455191.1
29	Hypsiglena sp.	FJ455194.1
30	Hypsiglena torquata	FJ455192.1
31	Imantodes cenchoa	EU390923.1
32	Imantodes cenchoa	FJ455187.1
33	Imantodes cenchoa	GQ334668.1
34	Imantodes cenchoa	GQ334669.1
35	Imantodes inornatus	GU353279.1
36	Lampropeltis getula	EU390925.1
37	Lamprophis fuliginosus	EU390926.1

	Typhlopoidea	Accession
1	Acutotyphlops kunuaensis	GU902590.1
2	Acutotyphlops sp	GU902629.1
3	Acutotyphlops subocularis	GU902589.1
4	Afrotyphlops angolensis	GU902562.1
5	Afrotyphlops bibronii	GU902620.1
6	Afrotyphlops congestus	GU902618.1
7	Afrotyphlops fornasinii	GU902617.1
8	Afrotyphlops lineolatus	GU902621.1
9	Afrotyphlops punctatus	GU902567.1
10	Ramphotyphlops diversus	GU902582.1
11	Ramphotyphlops endoterus	GU902570.1
12	Ramphotyphlops ganei	GU902583.1
13	Ramphotyphlops guentheri	GU902575.1
14	Ramphotyphlops howi	GU902585.1
15	Ramphotyphlops kimberleyensis	GU902577.1
16	Ramphotyphlops ligatus	GU902576.1
17	Ramphotyphlops pinguis	GU902586.1
18	Ramphotyphlops splendidus	GU902587.1
19	Ramphotyphlops troglodytes	GU902588.1
20	Megatyphlops mucruso	GU902560.1
21	Megatyphlops schlegelii	GU902619.1
22	Ramphotyphlops acuticauda	GU902554.1
23	Ramphotyphlops albiceps	GU902555.1
24	Ramphotyphlops australis	GU902580.1
25	Ramphotyphlops bicolor	GU902581.1
26	Ramphotyphlops bituberculatus	GU902574.1
27	Ramphotyphlops braminus	GU902556.1
28	Ramphotyphlops grypus	GU902584.1
29	Ramphotyphlops lineatus	GU902557.1
30	Ramphotyphlops longissimus	GU902579.1
31	Ramphotyphlops pilbarensis	GU902571.1
32	Ramphotyphlops polygrammicus	GU902591.1
33	Ramphotyphlops unguirostris	GU902578.1
34	Ramphotyphlops waitii	GU902573.1
35	Rhinotyphlops feae	GU902558.1
36	Rhinotyphlops lalandei	GU902559.1
37	Rhinotyphlops newtoni	GU902561.1

38	Lamprophis fuliginosus	FJ434094.1
39	Leioheterodon madagascariensis	FJ434093.1
40	Leptodeira annulata	FJ434099.1
41	Leptodeira annulata	GQ334670.1
42	Leptodeira annulata	GQ334671.1
43	Leptodeira annulata	GQ334672.1
44	Leptodeira bakeri	GQ334673.1
45	Leptodeira frenata	FJ810242.1
46	Leptodeira maculata	GQ334674.1
47	Leptodeira nigrofasciata	GQ334681.1
48	Leptodeira punctata	GQ334682.1
49	Leptodeira septentrionalis	FJ455188.1
50	Leptodeira septentrionalis	GQ334676.1
51	Leptodeira septentrionalis	GQ334677.1
52	Leptodeira septentrionalis	GQ334678.1
53	Leptodeira septentrionalis	GQ334675.1
54	Leptodeira septentrionalis	GQ334679.1
55	Leptodeira splendida	GQ334680.1
56	Liophis lineatus	DQ469793.1
57	Lycophidion capense	JN703031.1
58	Mehelya capensis	FJ434096.1
59	Natrix natrix	EU390931.1
60	Ninia atrata	GQ334683.1
61	Oxyrhopus petola	GQ334684.1
62	Pareas hamptoni	EU390933.1
63	Phyllorhynchus decurtatus	FJ434105.1
64	Psammophylax variabilis	FJ434097.1
65	Pseudoleptodeira latifasciata	FJ455190.1
66	Pseudoxenodon bambusicola	FJ434101.1
67	Rhadinaea fulvivittis	GU353275.1
68	Sibon nebulatus	FJ455189.1
69	Sibon nebulatus	GQ334685.1
70	Thamnophis marcianus	EU390935.1
71	Trachyboa boulengeri	EU390936.1
72	Trimorphodon biscutatus	EU390937.1
73	Xenochrophis flavipunctatus	FJ434102.1
74	Xenochrophis piscator	EU390941.1

38	Typhlops agoralionis	GU902592.1
39	Typhlops anchaurus	GU902593.1
40	Typhlops anousius	GU902615.1
41	Typhlops arator	GU902594.1
42	Typhlops arenarius	GU902624.1
43	Typhlops brongersmianus	GU902563.1
44	Typhlops capitulatus	GU902595.1
45	Typhlops catapontus	GU902596.1
46	Typhlops caymanensis	GU902597.1
47	Typhlops contorhinus	GU902616.1
48	Typhlops dominicanus	GU902598.1
49	Typhlops elegans	GU902564.1
50	Typhlops eperopeus	GU902614.1
51	Typhlops geotomus	GU902599.1
52	Typhlops granti	GU902600.1
53	Typhlops hedraeus	GU902565.1
54	Typhlops hypomethes	GU902601.1
55	Typhlops jamaicensis	GU902602.1
56	Typhlops lumbricalis	GU902603.1
57	Typhlops mirus	GU902566.1
58	Typhlops monastus	GU902604.1
59	Typhlops naugus	GU902605.1
60	Typhlops notorachius	GU902606.1
61	Typhlops pammeces	GU902628.1
62	Typhlops platycephalus	GU902607.1
63	Typhlops reticulatus	GU902568.1
64	Typhlops richardi	GU902608.1
65	Typhlops rostellatus	GU902609.1
66	Typhlops schwartzi	GU902610.1
67	Typhlops sp	GU902630.1
68	Typhlops sulcatus	GU902611.1
69	Typhlops sylleptor	GU902612.1
70	Typhlops syntherus	GU902613.1
71	Typhlops vermicularis	GU902569.1
72	Xenotyphlops grandidier	GU902627.2
73	Xenotyphlops sp	GU902626.2

	**Boidae**	**Accession**
1	Acrantophis dumerili	AY988049.1
2	Apodora papuana	FJ434076.1
3	Boa constrictor	AY988047.1
4	Boa constrictor occidentalis	HQ399534.1
5	Calabaria reinhardtii	AY988058.1
6	Calabaria reinhardtii	EU390911.1
7	Candoia carinata	AY988048.1
8	Candoia carinata	FJ434077.1
9	Charina bottae	AY988059.1
10	Charina trivirgata	DQ465578.1
11	Corallus caninus	AY988044.1
12	Corallus hortulanus	HQ399535.1
13	Epicrates alvarezi	HQ399531.1

	**Anguimorpha**	**Accession**
1	Abronia graminea	JQ844947.1
2	Anguis fragilis	JQ844948.1
3	Anniella pulchra	GU456008.1
4	Barisia imbricata	JQ844944.1
5	Celestus enneagrammus	GU456009.1
6	Diploglossus delasagra	JQ844945.1
7	Elgaria coerulea	JQ844949.1
8	Elgaria multicarinata	GU456010.1
9	Gerrhonotus infernalis	JQ844950.1
10	Heloderma horridum	GU456011.1
11	Heloderma suspectum	FJ434061.1
12	Heloderma suspectum	GU456012.1
13	Lanthanotus borneensis	GU456015.1

14	Epicrates angulifer	HQ399533.1
15	Epicrates assisi	HQ399529.1
16	Epicrates cenchria	AY988045.1
17	Epicrates cenchria	HQ399528.1
18	Epicrates chrysogaster	JF812171.1
19	Epicrates chrysogaster	JF812172.1
20	Epicrates striatus	DQ465554.1
21	Eryx colubrinus	DQ465569.1
22	Eryx colubrinus	EU390919.1
23	Eryx conicus	AY988057.1
24	Eunectes murinus	HQ399527.1
25	Eunectes notaeus	AY988046.1
26	Eunectes notaeus	HQ399526.1
27	Sanzinia madagascariensis	AY988050.1

	**Pythonidae**	**Accession**
1	Aspidites melanocephalus	DQ465558.1
2	Aspidites melanocephalus	EU390908.1
3	Morelia spilota	AY988052.1
4	Python reticulatus	FJ434074.1
5	Liasis savuensis	FJ434075.1

	**Iguania**	**Accession**
1	Anolis carolinensis	EU390900.1
2	Anolis carolinensis	XM_003221366.1
3	Basiliscus plumifrons	AY987998.1
4	Brachylophus fasciatus	AY988009.1
5	Brachylophus fasciatus	JF804533.1
6	Chalarodon madagascariensis	AY988001.1
7	Chalarodon madagascariensis	JF804536.1
8	Conolophus pallidus	HM352521.1
9	Conolophus subcristatus	HM352522.1
10	Crotaphytus collaris	AY987997.1
11	Crotaphytus collaris	JF804542.1
12	Cyclura carinata	HM352524.1
13	Cyclura nubila	FJ434062.1
14	Cyclura pinguis	GQ853278.1
15	Cyclura ricordi	HM352523.1
16	Dipsosaurus dorsalis	JF804545.1
17	Enyalioides laticeps	GU456004.1
18	Gambelia wislizenii	EU108024.1
19	Gambelia wislizenii	JF804548.1
20	Iguana iguana	HM352529.1
21	Iguana iguana	HM352530.1
22	Leiocephalus barahonensis	JF804551.1
23	Leiosaurus catamarcensis	JF804553.1
24	Liolaemus bellii	JF804554.1
25	Liolaemus elongatus	JN703030.1
26	Liolaemus pictus	AY988003.1
27	Morunasaurus annularis	JF804556.1
28	Oplurus cuvieri	AY988000.1

14	Pseudopus apodus	GU456007.1
15	Pseudopus apodus	JQ844946.1
16	Shinisaurus crocodilurus	GU456013.1
17	Shinisaurus crocodilurus	JQ844951.1
18	Varanus acanthurus	GU456016.1
19	Varanus acanthurus	JQ844905.1
20	Varanus albigularis	JQ844940.1
21	Varanus baritji	JQ844906.1
22	Varanus brevicauda	JQ844907.1
23	Varanus bushi	JQ844908.1
24	Varanus caudolineatus	JQ844909.1
25	Varanus doreanus	JQ844941.1
26	Varanus dumerilii	JQ844942.1
27	Varanus eremius	JQ844910.1
28	Varanus exanthematicus	GU456017.1
29	Varanus exanthematicus	JQ844911.1
30	Varanus giganteus	JQ844912.1
31	Varanus gilleni	JQ844913.1
32	Varanus glauerti	JQ844914.1
33	Varanus glebopalma	JQ844915.1
34	Varanus gouldii	JQ844916.1
35	Varanus indicus	JQ844917.1
36	Varanus jobiensis	JQ844938.1
37	Varanus keithhornei	JQ844918.1
38	Varanus kingorum	JQ844919.1
39	Varanus komodoensis	JQ844920.1
40	Varanus mertensi	JQ844921.1
41	Varanus mitchelli	JQ844922.1
42	Varanus niloticus	JQ844923.1
43	Varanus panoptes	JQ844924.1
44	Varanus panoptes	JQ844925.1
45	Varanus pilbarensis	JQ844926.1
46	Varanus prasinus	JQ844927.1
47	Varanus primordius	JQ844928.1
48	Varanus rosenbergi	JQ844929.1
49	Varanus rudicollis	JQ844943.1
50	Varanus salvadorii	JQ844930.1
51	Varanus salvator	EU390902.1
52	Varanus salvator	JQ844931.1
53	Varanus scalaris	JQ844932.1
54	Varanus semiremex	JQ844933.1
55	Varanus spenceri	JQ844934.1
56	Varanus storri	JQ844935.1
57	Varanus timorensis	JQ844936.1
58	Varanus tristis	JQ844937.1
59	Varanus varius	JQ844939.1
60	Xenosaurus grandis	GU456014.1
61	Xenosaurus platyceps	EU390901.1

29	Oplurus cyclurus	GU456006.1
30	Petrosaurus mearnsi	JF804557.1
31	Phrynosoma cornutum	AY988004.1
32	Phrynosoma platyrhinos	JF804559.1
33	Phymaturus calcogaster	JX969565.1
34	Phymaturus ceii	JX969566.1
35	Phymaturus delheyi	JX969582.1
36	Phymaturus dorsimaculatus	JX969596.1
37	Phymaturus etheridgei	JX969564.1
38	Phymaturus excelsus	JX969567.1
39	Phymaturus extrilidus	JX969605.1
40	Phymaturus felixi	JX969578.1
41	Phymaturus felixi	JX969579.1
42	Phymaturus indistinctus	JX969568.1
43	Phymaturus laurenti	JX969595.1
44	Phymaturus mallimaccii	JX969597.1
45	Phymaturus manuelae	JX969569.1
46	Phymaturus nevadoi	JX969570.1
47	Phymaturus palluma	JF804560.1
48	Phymaturus palluma	JX969598.1
49	Phymaturus patagonicus	JX969571.1
50	Phymaturus payuniae	JX969572.1
51	Phymaturus punae	JX969599.1
52	Phymaturus querque	JX969604.1
53	Phymaturus roigorum	JX969600.1
54	Phymaturus sitesi	JX969584.1
55	Phymaturus somuncurensis	JX969573.1
56	Phymaturus sp.	JX969614.1
57	Phymaturus spectabilis	JX969574.1
58	Phymaturus spurcus	JX969575.1
59	Phymaturus tenebrosus	JX969576.1
60	Phymaturus verdugo	JX969601.1
61	Phymaturus vociferator	JX969602.1
62	Phymaturus zapalensis	JX969577.1
63	Polychrus marmoratus	JF804564.1
64	Pristidactylus torquatus	JF804565.1
65	Sauromalus ater	HM352525.1
66	Sauromalus ater	JF804568.1
67	Sauromalus australis	HM352526.1
68	Sceloporus horridus	AY988005.1
69	Sceloporus variabilis	JF804569.1
70	Stenocercus guentheri	JF804570.1
71	Tropidurus hispidus	AY987996.1
72	Uma scoparia	JF804574.1
73	Uranoscodon superciliosus	JF804575.1
74	Urostrophus vautieri	JF804576.1
75	Uta stansburiana	JF804577.1

	**Scinciformata**	**Accession**
1	Acontias meleagris	GU456026.1
2	Amphiglossus splendidus	JN568316.1
3	Brachymeles gracilis	JN568322.1

	**Crocodiles**	**Accession**
1	Alligator mississipiensis	AJ316234.1
2	Alligator sinensis	EF646331.1
3	Caiman crocodilus	EF646321.1
4	Caiman latirostris	EF646322.1
5	Caiman yacare	EF646323.1
6	Crocodylus acutus	EF646333.1
7	Crocodylus intermedius	EF646334.1
8	Crocodylus johnsoni	EF646325.1
9	Crocodylus mindorensis	EF646326.1
10	Crocodylus moreletii	EF646335.1
11	Crocodylus niloticus	EF646338.1
12	Crocodylus novaeguineae	EF646327.1
13	Crocodylus palustris	EF646337.1
14	Crocodylus porosus	EF646336.1
15	Crocodylus rhombifer	EF646332.1
16	Crocodylus siamensis	EF646324.1
17	Gavialis gangeticus	EF646342.1
18	Mecistops cataphractus	EF646339.1
19	Melanosuchus niger	EF646328.1
20	Osteolaemus tetraspis	EF646340.1
21	Paleosuchus palpebrosus	EF646329.1
22	Paleosuchus trigonatus	EF646330.1
23	Tomistoma schlegelii	EF646341.1

	**Mammals**	**Accession**
1	Ailuropoda melanoleuca	XM_002926828.1
2	Bos taurus	BC123576.1
3	Bos taurus	NM_001077988.2
4	Callithrix jacchus	XM_002752241.1
5	Canis lupus	XM_543858.3
6	Canis lupus	XM_003433475.1
7	Cavia porcellus	XM_003463303.1
8	Cricetulus griseus	XM_003496037.1
9	Equus caballus	XM_001495245.3
10	Felis catus	NM_001009367.1
11	Gorilla gorilla	XM_004052539.1
12	Gorilla gorilla	XM_004052540.1
13	Homo sapien	M37763.1
14	Homo sapien	M61180.1
15	Homo sapien	X53655.1
16	Homo sapien	BC069773.1
17	Homo sapien	CR541906.1
18	Homo sapien	BC107075.1
19	Homo sapien	NM_002527.4
20	Homo sapien	NM_001102654.1
21	Homo sapien	AK293895.1
22	Loxodonta africana	XM_003410779.1

4	Cordylus namaquensis	AY988010.1
5	Eugongylus rufescens	JN568336.1
6	Feylinia polylepis	GU456027.1
7	Mabuya quinquetaeniata	EU108028.1
8	Mabuya quinquetaeniata	JN568320.1
9	Plestiodon chinensis	EF646344.1
10	Plestiodon fasciatus	JF804547.1
11	Plestiodon laticeps	EU108023.1
12	Scincus scincus	GU456028.1
13	Sphenomorphus simus	EU108031.1
14	Sphenomorphus solomonis	JN568332.1
15	Tiliqua scincoides	JN568330.1

	**Turtles**	**Accession**
1	Chelydra serpentina	JN568325.1
2	Chinemys reevesi	EF646343.1
3	Erymnochelys madagascariensis	AY988085.1
4	Hydromedusa tectifera	AY988090.1
5	Pelomedusa subrufa	AY988088.1
6	Peltocephalus dumeriliana	AY988087.1
7	Pelusios gabonensis	AY988089.1
8	Podocnemis erythrocephala	FM165607.1
9	Podocnemis expansa	AY988086.1
10	Podocnemis expansa	JN568331.1
11	Podocnemis lewyana	FM165605.1
12	Podocnemis sextuberculata	FM165608.1
13	Podocnemis unifilis	FM165606.1
14	Podocnemis vogli	FM165609.1
15	Staurotypus triporcatus	AY988091.1

23	Macaca fuscata	AF222683.1
24	Macaca mulatta	XM_001118191.2
25	Monodelphis domestica	XM_001368543.2
26	Mus musculus	X53257.1
27	Mus musculus	BC065785.1
28	Mus musculus	AK132586.1
29	Mus musculus	NM_008742.3
30	Mus musculus	NM_001164034.1
31	Mus musculus	NM_001164035.1
32	Nomascus leucogenys	XM_003273711.1
33	omascus leucogenys	XM_003273712.1
34	Ornithorhynchus	XM_001507068.1
35	Oryctolagus cuniculus	XM_002712831.1
36	Ovis aries	XM_004006944.1
37	Ovis aries	XM_004006945.1
38	Pan paniscus	XM_003820329.1
39	Pan troglodytes	XM_522331.3
40	Papio anubis	XM_003905836.1
41	Pongo abelii	XM_002822796.1
42	Rattus norvegicus	M34643.1
43	Rattus norvegicus	M33968.1
44	Rattus norvegicus	M61179.1
45	Rattus norvegicus	NM_031073.3
46	Rattus norvegicus	NM_001270869.1
47	Rattus norvegicus	NM_001270868.1
48	Rattus norvegicus	NM_001270870.1
49	Saimiri boliviensis	XM_003942177.1
50	Sarcophilus harrisii	XM_003771238.1
