# Supplementary figures and images for: Molecular Evolution of Vertebrate Neurotrophins: Co-Option of the Highly Conserved Nerve Growth Factor Gene into the Advanced Snake Venom Arsenalf
Source: PLoS One. 2013 Nov 29;8(11):e81827. doi: 10.1371/journal.pone.0081827 (PMC3843689; doi:10.1371/journal.pone.0081827)

# S1. Molecular phylogeny of Nerve Growth Factors (NGF)

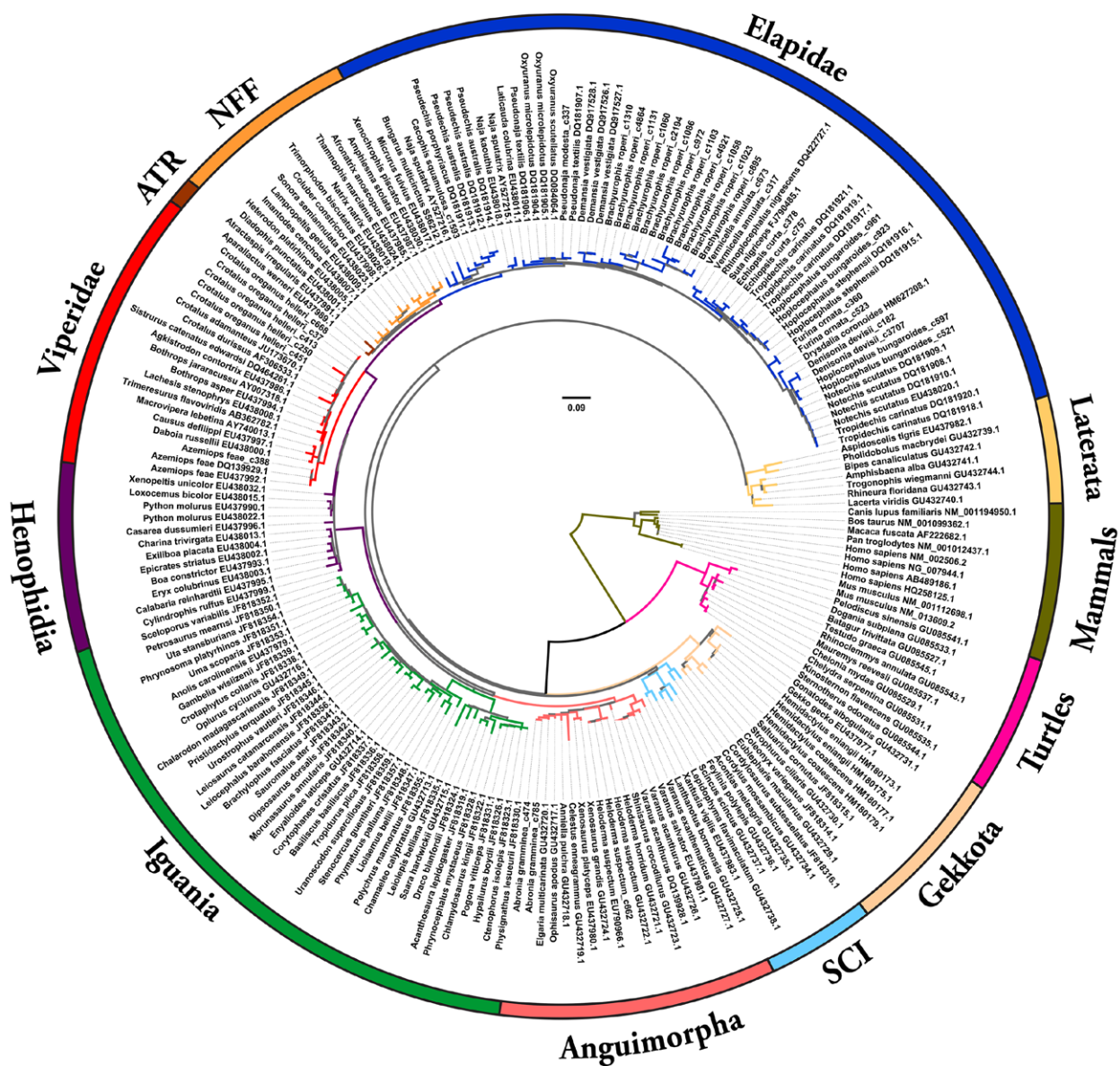

Supplement: Figure S1 — Maximum-likelihood molecular phylogeny of nerve growth factors (NGF). Branches with bootstrap support of less than 850 (out of 1000 bootstrap replicates) are highlighted in grey. [NFF: “non-front-fanged” advanced snakes; Atr: Atractaspidinae ; Sci: Scinciformata]. (PDF) [file pone.0081827.s006.pdf]

S2. Molecular phylogeny of Brain-derived Neurotrophic Factors (BDNF)

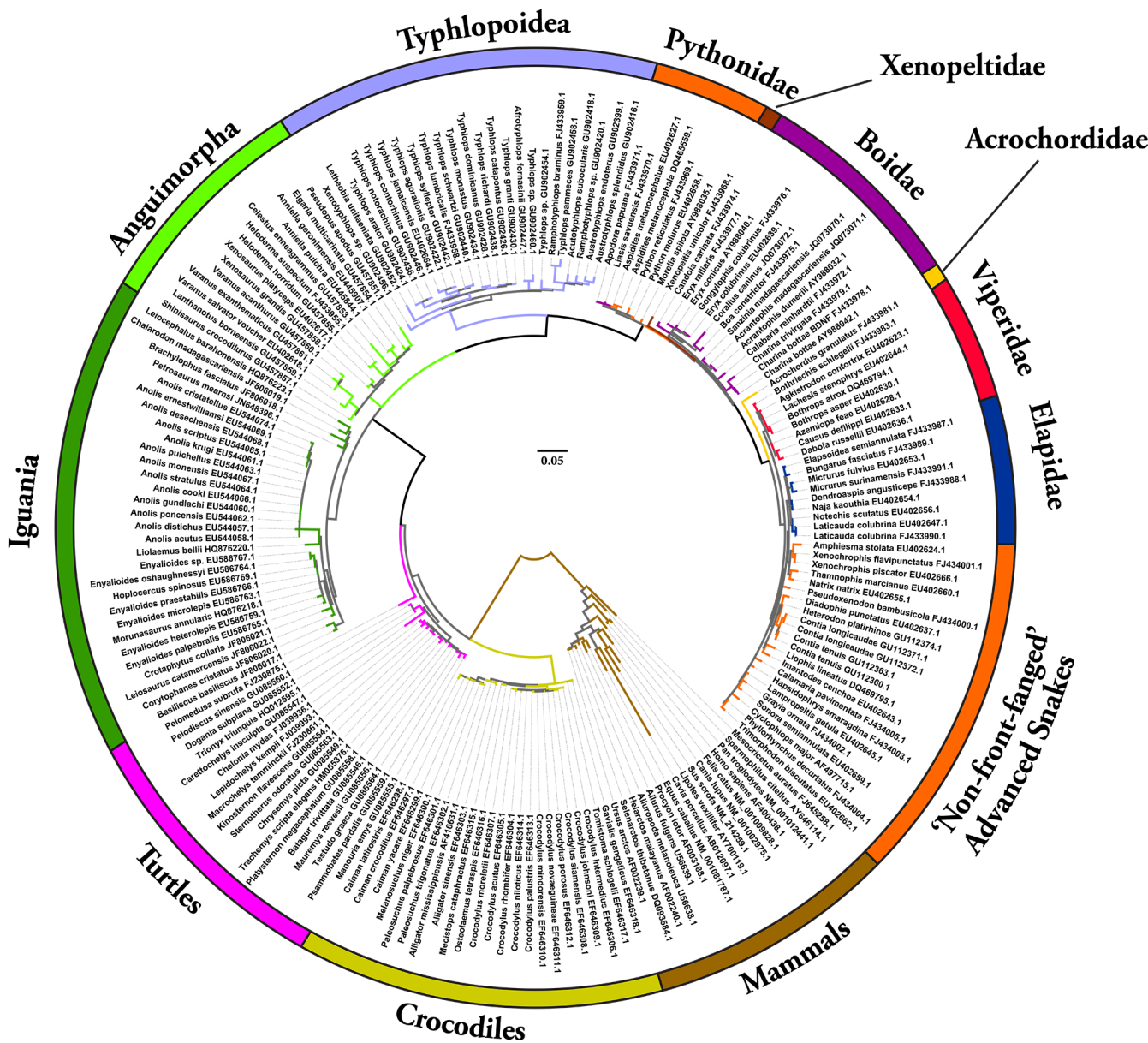

Supplement: Figure S2 — Maximum-likelihood molecular phylogeny of brain-derived neurotrophic factors (BDNF). Branches with bootstrap support of less than 850 (out of 1000 bootstrap replicates) are highlighted in grey. (PDF) [file pone.0081827.s007.pdf]

S3. Molecular phylogeny of Neurotrophin-3 (NT-3)

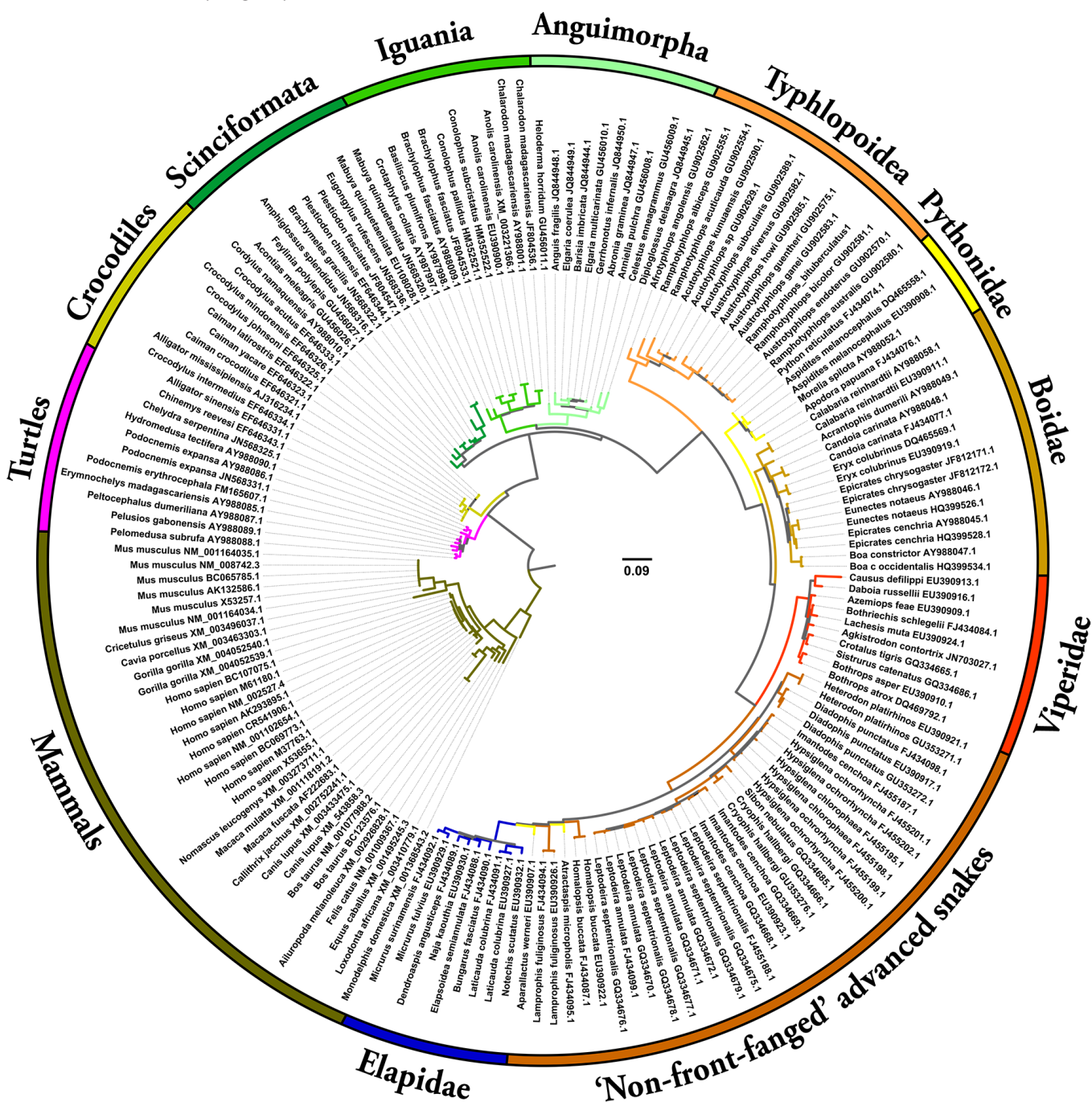

Supplement: Figure S3 — Maximum-likelihood molecular phylogeny of neurotrophin-3 (NT3). Branches with bootstrap support of less than 850 (out of 1000 bootstrap replicates) are highlighted in grey. (PDF) [file pone.0081827.s008.pdf]

## S5. Alignment of vertebrate Nerve Growth Factors (NGF)

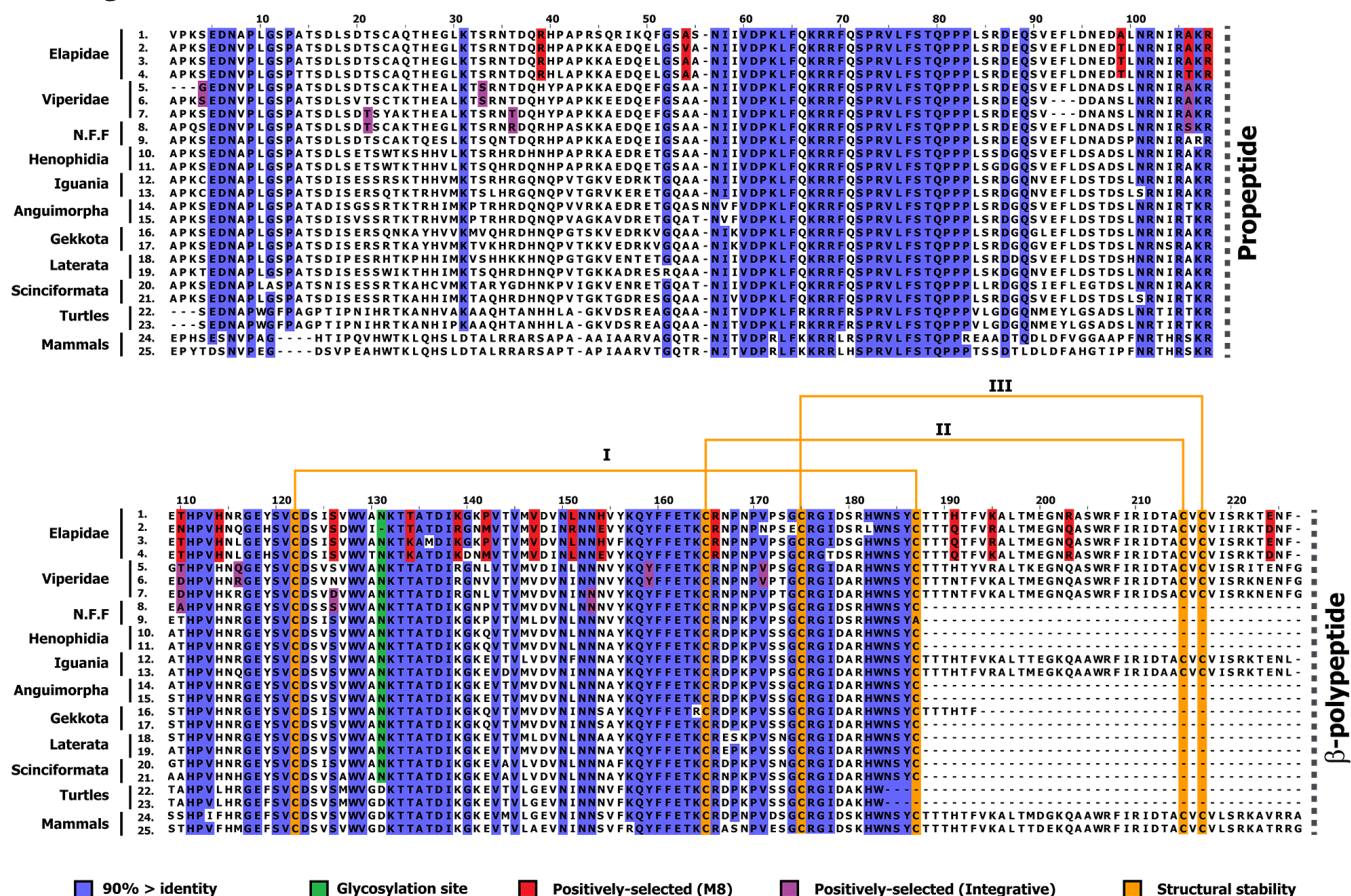

Supplement: Figure S5 — Alignment of vertebrate nerve growth factors. (PDF) [file pone.0081827.s010.pdf]

## S6. Evolutionaryfingerprint of Nerve Growth Factors (NGF)

### Evolutionaryfingerprint

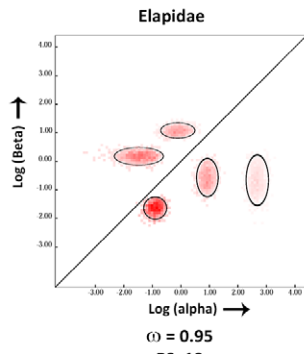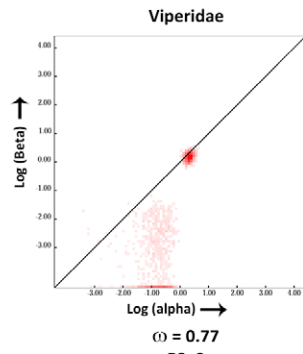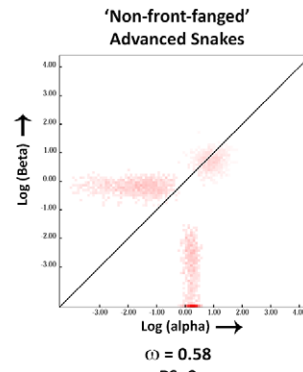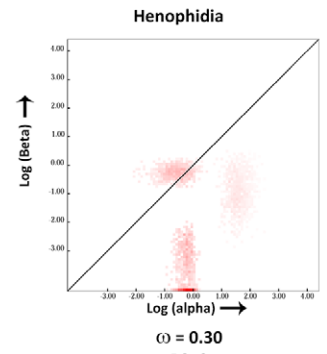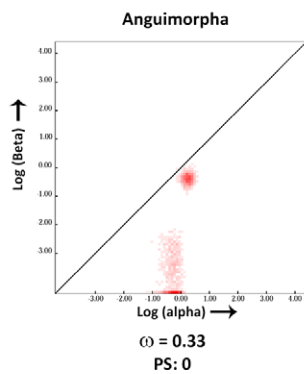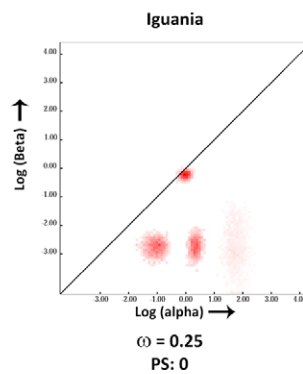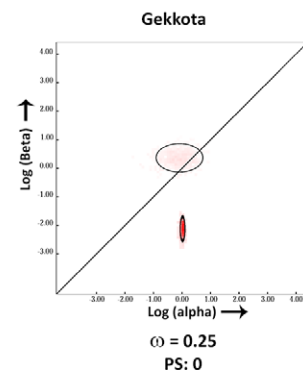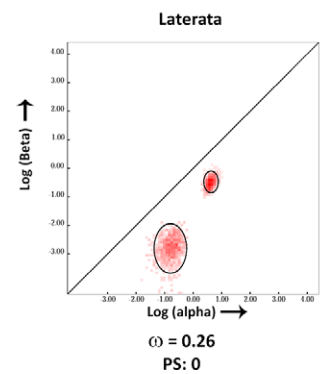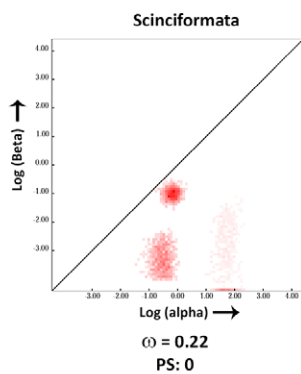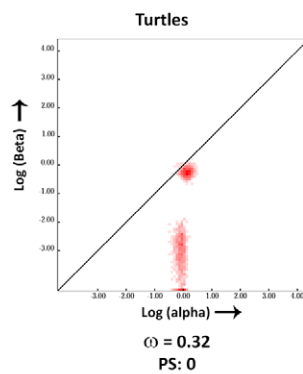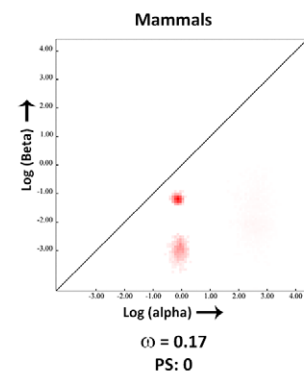

Supplement: Figure S6 — Evolutionary fingerprint of nerve growth factors (NGF). Estimates of the distribution of synonymous (a) and non-synonymous (b) substitution ratesinferred for various reptilian and mammalian nerve growth factor (NGF) lineages are shown here. The ellipses reflect a Gaussian-approximated variance in each individual rate estimate, and coloured pixels show the density of the posterior sample of the distribution for a given rate. The diagonal line represents the idealized neutral evolution regime (ω = 1), points above and below the line correspond to positive selection (ω>1) and negative selection (ω<1), respectively. Site model 8 omega (w) along with the total number of positively selected sites detected by its Bayes Empirical Bayes (BEB) approach are also indicated below. (PDF) [file pone.0081827.s011.pdf]

## 8. Evolutionary fingerprint of Neurotrophin-3 (NT-3)

### Evolutionary fingerprint

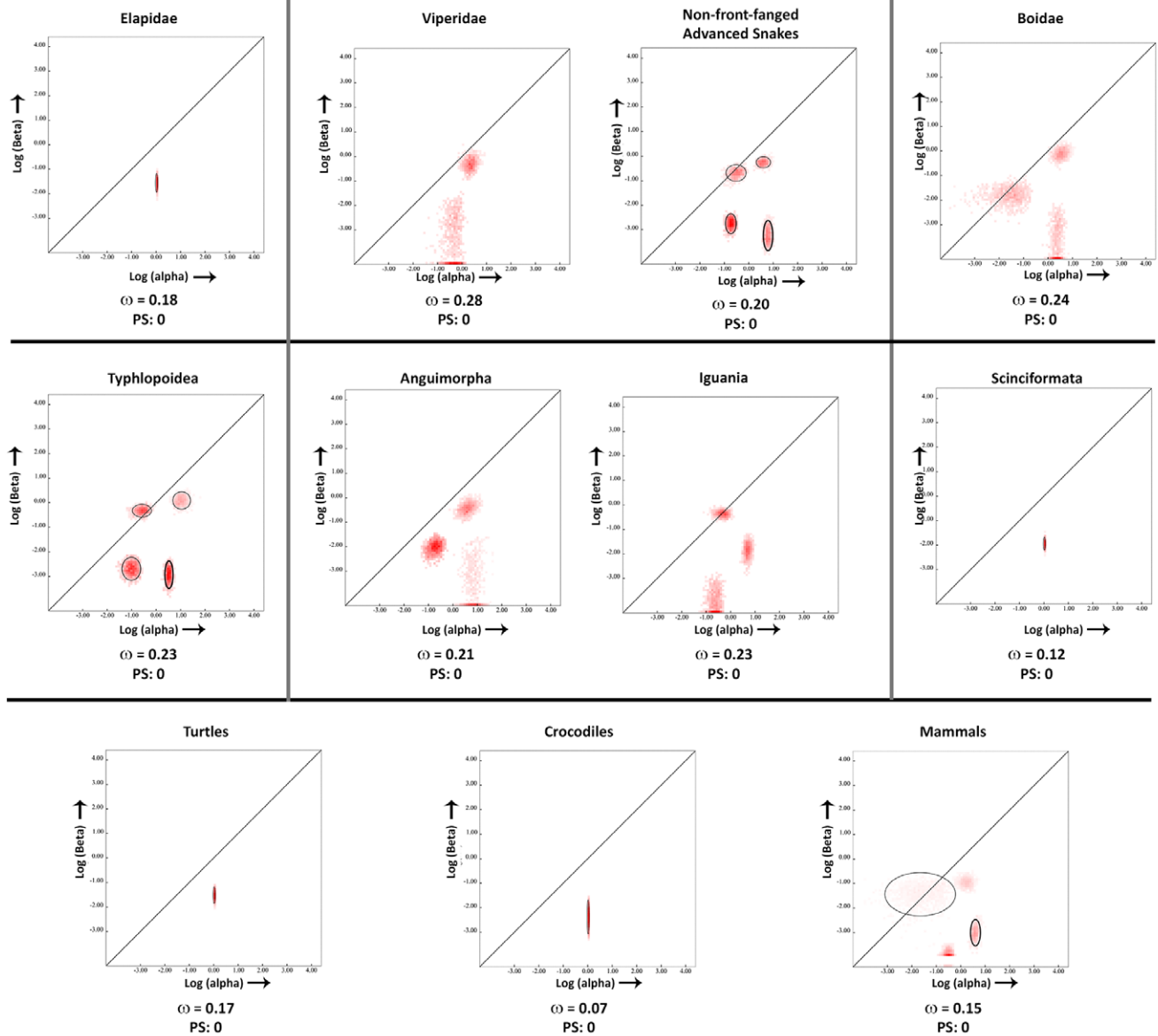

Supplement: Figure S8 — Evolutionary fingerprint of neurotrophin-3 (NT3). Estimates of the distribution of synonymous (a) and non-synonymous (b) substitution rates inferred for various reptilian and mammalian neurotrophin-3 (NT-3) lineages are shown here. The ellipses reflect a Gaussian-approximated variance in each individual rate estimate, and coloured pixels show the density of the posterior sample of the distribution for a given rate. The diagonal line represents the idealized neutral evolution regime (ω = 1), points above and below the line correspond to positive selection (ω>1) and negative selection (ω<1), respectively. Site model 8 omega (w) along with the total number of positively selected sites detected by its Bayes Empirical Bayes (BEB) approach is also indicated below. (PDF) [file pone.0081827.s013.pdf]
