# Supplementary material for: Molecular Evolution of Vertebrate Neurotrophins: Co-Option of the Highly Conserved Nerve Growth Factor Gene into the Advanced Snake Venom Arsenalf
Source: PLoS One. 2013 Nov 29;8(11):e81827. doi: 10.1371/journal.pone.0081827 (PMC3843689; doi:10.1371/journal.pone.0081827)

S4. **Molecular phylogeny of Nerve Growth Factor (NGF), Brain-derived Neurotrophic factor and Neurotrophin-3 (NT-3) genes**

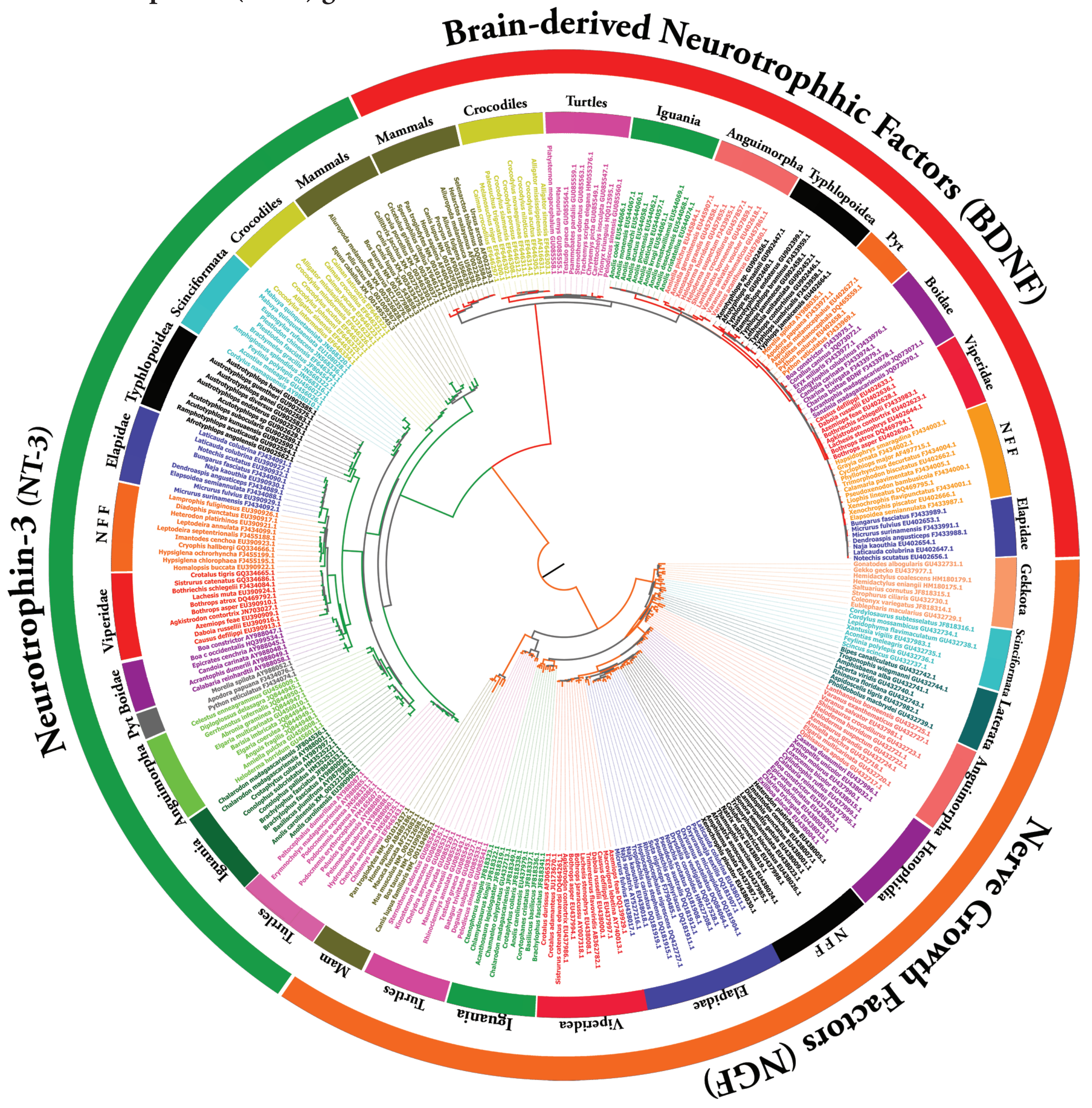

Supplement: Figure S4 — Maximum-likelihood phylogeny of neurotrophins. Branches with bootstrap support of less than 850 (out of 1000 bootstrap replicates) are highlighted in grey. [NFF: ‘non-front-fanged’ advanced snakes; Pyt: Pythonidae; Mam: Mammals]. (PDF) [file pone.0081827.s009.pdf]
