# Supplementary material for: Molecular Evolution of Vertebrate Neurotrophins: Co-Option of the Highly Conserved Nerve Growth Factor Gene into the Advanced Snake Venom Arsenalf
Source: PLoS One. 2013 Nov 29;8(11):e81827. doi: 10.1371/journal.pone.0081827 (PMC3843689; doi:10.1371/journal.pone.0081827)

S7. Evolutionaryfingerprint of Brain-derived Neurotrophic Factors (BDNF)

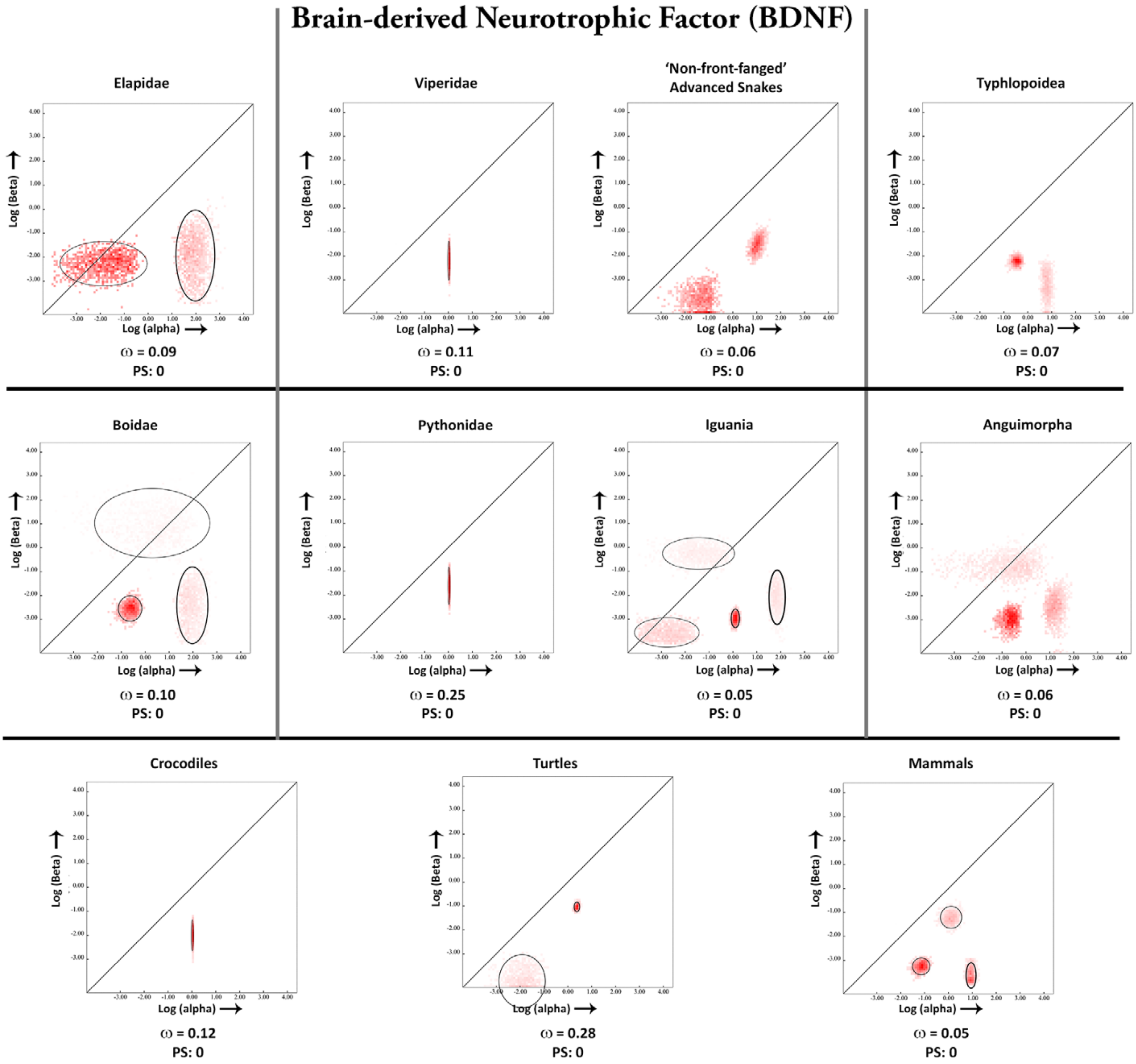

Supplement: Figure S7 — Evolutionary fingerprint of brain-derived neurotrophic factors (BDNF). Estimates of the distribution of synonymous (a) and non-synonymous (b) substitution rates inferred for various reptilian and mammalian brain-derived neurotrophic factor (BDNF) lineages are shown here. The ellipses reflect a Gaussian-approximated variance in each individual rate estimate, and coloured pixels show the density of the posterior sample of the distribution for a given rate. The diagonal line represents the idealized neutral evolution regime (ω = 1), points above and below the line correspond to positive selection (ω>1) and negative selection (ω<1), respectively. Site model 8 omega (w) along with the total number of positively selected sites detected by its Bayes Empirical Bayes (BEB) approach are also indicated below. (PDF) [file pone.0081827.s012.pdf]
