# Supplementary material for: Molecular Evolution of Vertebrate Neurotrophins: Co-Option of the Highly Conserved Nerve Growth Factor Gene into the Advanced Snake Venom Arsenalf
Source: PLoS One. 2013 Nov 29;8(11):e81827. doi: 10.1371/journal.pone.0081827 (PMC3843689; doi:10.1371/journal.pone.0081827)

9. Branch-site Random Effects Likelihood

A. Elapidae nerve growth factors (NGF)

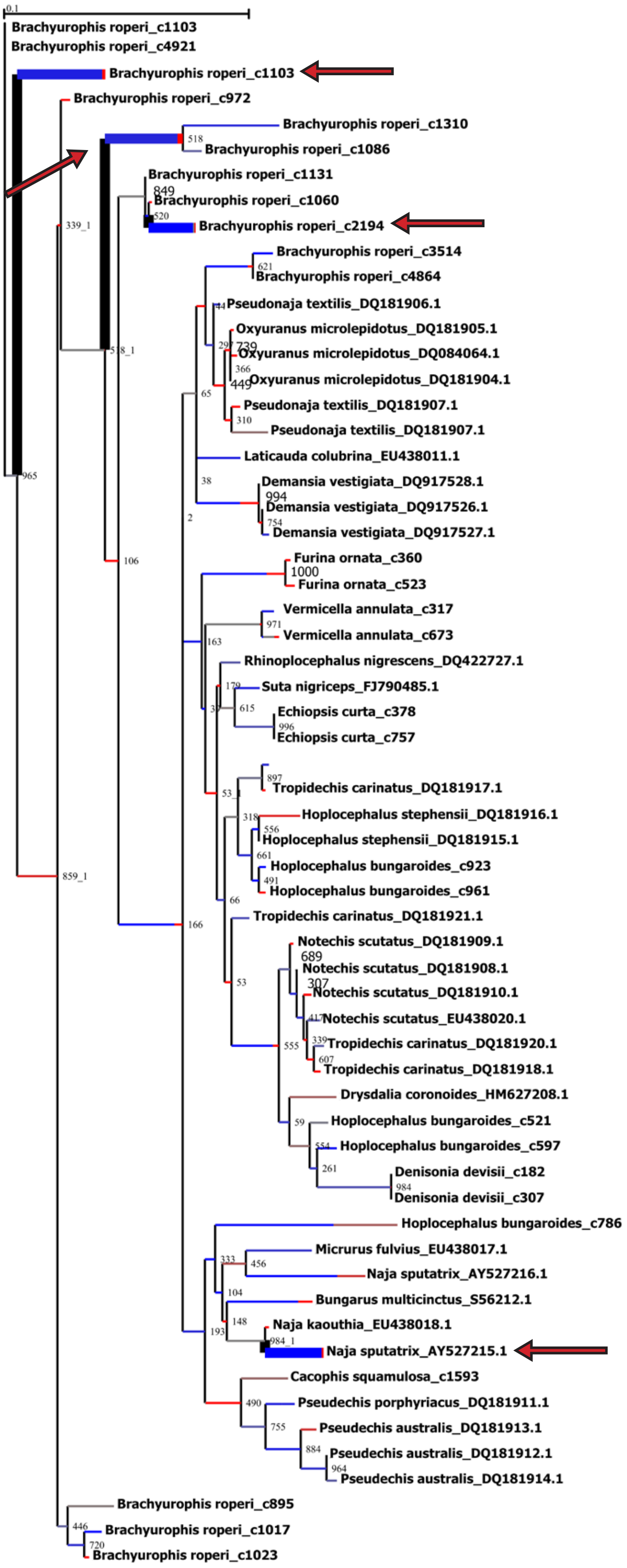

B. Viperidae nerve growth factors (NGF)

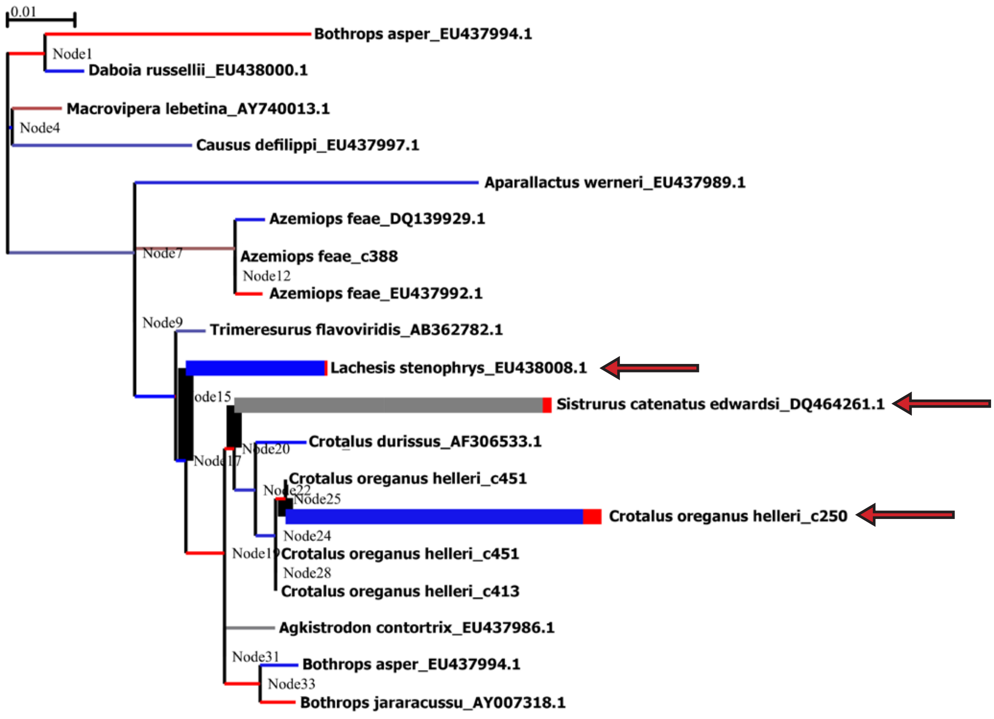

C. 'Non-front-fanged' advanced snake nerve growth factors (NGF)

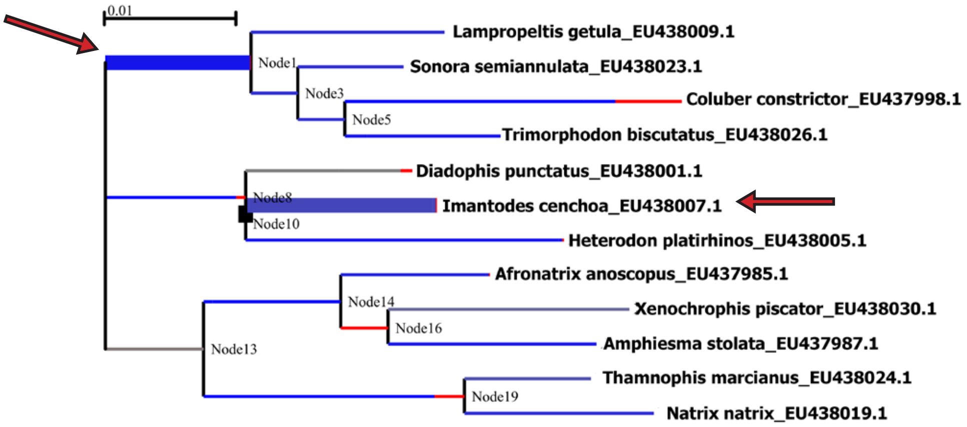

Supplement: Figure S9 — Branch-site REL: Caenophidian nerve growth factors (NGF). The hue of each colour indicates strength of selection, with primary red corresponding to ω > 5, primary blue to ω = 0 and grey to ω=1. The width of each colour component represents the proportion of sites in the corresponding class. Thicker branches have been classified as undergoing episodic diversifying selection (indicated by arrows) by the sequential likelihood ratio test at corrected p ≤ 0.05. (PDF) [file pone.0081827.s014.pdf]
