# Supplementary material for: Molecular Evolution of Vertebrate Neurotrophins: Co-Option of the Highly Conserved Nerve Growth Factor Gene into the Advanced Snake Venom Arsenalf
Source: PLoS One. 2013 Nov 29;8(11):e81827. doi: 10.1371/journal.pone.0081827 (PMC3843689; doi:10.1371/journal.pone.0081827)
Supplement: File S1 — Unpublished sequences. (DOCX) [file pone.0081827.s015.docx]

>Bse_RL2_rep_c1017

ATGCATAGCGTAACATCCATGTTGTGCTACACTCTGATTATAGCATTTCTGATTGGCATATGGGCAGCACCAAAATCTGAAGATAATGTTCCTCTGGGCTCCCCTGCAACATCTGACCTTTCTGACACCAGCTGTGCTCAAACTCACGAGGGTCTGAAAATATCTCTAAATACAGATCAGCGCCATCCTGCTCCTAAGAAGGCAGAAGATCAAGAACTTGGATCAGCAGCCAACATCATTGTGGATCCCAAGCTTTTC

>Bse_RL2_rep_c1023

CCCCCTGCAACATCTGACCTTTCTGACACCAGCTGTGCTCAAACTCACGAGGGTCTGAAAATATCTCTAAATACAGATCAGCGCCATCCTGCTCCTAAGAAGGCAGAAGATCAAGAACTTGGATCAGCAGCCAACATCATTGTGGATCCCAAGCTTTTTCAGAAGAGGCGTTTCCAGTCACCTCGTGTTCTGTTCAGCACTCAGCCCCCACCGTTGTCAAGAGATGAGCAAAGTGTGGAGTTCCCCGACAATGAAGACGCTCTTAACATGAGTATCCGGGCCAAACGTGAAAATCATCCTGCGTATAATGTAGGGGAACATTCTGTGTGTGACAGTATCAATAGCTGGGTTACCAAAACCACAGCAACGGACATCCACGACAATAGGGTGACTGTGACGGCGGAGATAAATATTAATAACCATTTCTACAAGCAGTACTTTTTTGAAACCAAGTGCATAAATCCAAATCCAGTACCGAGTGGATGCAGGGGCATTGATTCCAGGTATTGGAATTCTTATTGCACCACAACACAGTCATTTGTCATGGCATTAACCATGGAAGGCAATCGGACATCCTGGCGCTTCATTCGGATTGACACTGCCTGTGTGTGCGTAATCAGTAGAAAATCTGACAGCTTC

>Bse_RL2_rep_c1058

ATGCATAGCGTAACGTCCATGTTGTGCTACACTCTGATTATAGCATTTCTGATTGGTATATGGGCAGCACCAAAATCTGAAGATAATGTCCCTCTGGGCTTCCCTGCAACATCTGACCTTTATGACACCAGCTGTGCTCAAACTCACAAGGGTCTGAAAACATCTCTAAATACAGATCAGCGCCATCCTGCTCCTAAGAAGGCAGAGGATCAAGAACTTGGATCAGCAGCCAACATCATTGTGGATCCCAAGCTTTTTCAGAAGAGGCGTTTCCAGTCACCTCGTGTTCTGTTCTGCACTCAGCCCCCACCGTTGTCAAGAGATGAGCAAAGTATGGAGCTCCCCGACAATGAAGATGCTCTTAACATGAGTATCTGGGCCCAACGTGAAAATCATCCTGCGTATAATGTAGGGGAACATTCTGTGTGTGACAGTATCAATAGCTGGGTTACCAAAACCACAGCAACGGACATCTACGACAATAGGGTGATTGTGACGGCGGGGATAAATCTTAATAACCATGTCTACAAGCAGTACTTTTTTGAAACCAAGTGCAGAAATCCAAATCCAGTACCGAGTGGGTGCAGGGGCATTGATTCCAGGTATTGGAATTCTTATTGCACCACAACACAGTCATTTGTCAAGGCATTAACCATGGAAGGCAATCAGGCATCCTGGCGCTTCATTCGGATTGACACTGCCTGTGTGTGCGTAATCAGTAGAAAATCTGACAGCTTC

>Bse_RL2_rep_c1060

ATGCATAGCGTAACGTCCATGTTGTGCTACACTCTGATTATAGCATTTCTGATTGGCATATGGGCAGCACCAAAATCTGAAGATAATGTCCCTCTGGACTCCCCTGCAACATCTGACCTTTCTGACACCAGCTGTGCTCAAACTCACGAGGGTCTGAAAACATCTCTAACTACAGATCAGCGCCATCCTGCTCCTAAGAAGGCAGAAGATCAAGAACTTGGATCAGCAGCCAACATCATTGTGGATCCCAAGCTTTTTCAGAAGAGGCGTTTCCAGTCACCTCGTGTTCTATTCAGCACTCAGCCCCCACCATTGTCAAGAGATGAGCAAAGTGTGGAGTTCCTGGACAATGAAGACACTCTTAACAGGAATATCCGGACCAAACGTGAAACTCATCCTGTGCATAACCGAGGGGAATATTCTGTGTGTGACAGTATCGATAGCTGGGTTACCAAAACCACAGCAACGGACATCCACGACAATAGGGTGACTGTGATGGTGGATGTAAATCTTAATAACCATTTCTACAAGCAGTACTTTTTTGAAACCAAGTGCACAAATCCAAATCCATTACCGAGTGGGTGCAGGGGCATTGATTCCAGGTATTGGAATTCTTATTGCACCACAACACAGTCATTTGTCATGGCATTAACCATGGAAGGCAATCGGACATCCTGGCGCTTCATTCGGATTGACACTGCCTGTGTGTGCGTAATCAGTAGAAAATCTGACAGCTTC

>Bse_RL2_rep_c1086

ATGCATAGCGTAACGTCCATGTTGTGCTACACTCTGATTATAGCATTTCTGATTGGCATATGGGCAGCACCAAAATCTGAAGATAATGTCCCTCTGGGCTCCCCTGCAACATCTGACCTTTCTGACACCAGCTGTGCTCAAACTCACAAGGGTCTGAAAACATCTCTAAATACAGATCAGCACCATCCTGCTCCTAAGAAGGCAGAGGATCAAGAACTTGGATCAGTAGCAAACATCATTGTGGATCCCAAGCTTTTTCAGAAGAGGCGTTTCCAGTCACCTCGTGTTCTGTTCAGCACTCAGCCCCCACCGTTGTCAAGAGATGAGCAAAGTGTGGAGTTCCTGGACAATGAAGACACTCTTAACAGGAATATCCGGACAAAC

>Bse_RL2_rep_c1103

ATGCATAGCATAACCTCCATGTTGTGCTACACTCTGATTATAGCATTTCTGATTGGTATATGGGCAGCACCAAAATCTGAAGATAATGTCCCTCTGGGCTCCCCTGCAACATCTGACCTTTATGACACCAGCTGTGCTCAAACTCACAAGGGTCTGAAAACATCTCTAAATACAGATCAGCGCCATCCTGCTCCTAAGAAGGCAGTGGATCAAGAACTTGGATCAGCAGCCAACATCATTGTGGATCCCAAGCTTTTTCAGAAGAGGCGTTTCCAGTCACCTCGTGTTCTGTTCAGCACTCAGCCCCCACCATTGTCAAGAGATGAGCAAAGTATGGAGCTCCCCGACAATGAAGACGCTCTTAACATGAGTATCTGGGCCCAACGTGAAAATCATCCTGCGTATAATGTAGGGGAACATTCTGTGTGTGACAGTATCAATAGCTGGGTTACCAAAACCACAGCAACGGACATCTACGACAATAGGGTGATTGTGACGGCGGGGATAAATCTTAATAACCATCGTCTACAAGCAGTACTTTTT

>Bse_RL2_rep_c1131

ATGCATAGCGTAACGTCCATGTTGTGCTACACTCTGATTATAGCATTTCTGATTGGCATATGGGCAGCACCAAAATCTGAAGATAATGTCCCTCTGGACTCCCCTGCAACATCTGACCTTTCTGACACCAGCTGTGCTCAAACTCACGAGGGTCTGAAAACATCTCTAACTACAGATCAGCGCCATCCTGCTCCTAAGAAGGCAGAAGATCAAGAACTTGGATCAGCAGCCAACATCATTGTGGATCCCAAGCTTTTTCAGAAGAGGCGTTTCCAGTCACCTCGTGTTCTATTCAGCACTCAGCCCCCACCGTTGTCAAGAGATGAGCAAAGTGTGGAGTTCCTGGACAATGAAGACACTCTTAACAGGAATATCCGGACCAAACGTGAAACTCATCCTGTGCATAACCGAGGGGAATATTCTGTGTGTGACAGTATCGATAGCTGGGTTACCAAAACCACAGCAACGGACATCCACGACAATAGGGTGACTGTGATGGTGGATGTAAATCTTAATAACCATGTCTACAAGCAGTACTTTTTTGAAACCAAGTGCACAAATCCAAATCCATTACCGAGTGGGTGCAGGGGCATTGATTCCAGGTATTGGAATTCTTATTGCACCACAACACAGTCATTTGTCATGGCATTAACCATGGAAGGCAATCGGACATCCTGGCGCTTCATTCGG

>Bse_RL2_rep_c1310

ATGAAGGTGCATAGCGTAATGTCCATGCTGTGCTACACTCTGATTATAGCATTTCTGATTGGCATATGGGCAGCACCAAAATCTGAAGATAATGTCCCTCTGGGCTCCCCTGCAACATCTGACCTTTCTGACACCAGCTGTGCTCAAACTCACAAGGGTCTGAAAACATCTCTAAATACAGATCAGCGCCATCCTGCTCCTAAGAAGGCAGAGGATCAAGAACTTGGATCAGCAGCCAACATCATTGTGGATCCCAAGCTTTTTCAGAAGAGGCGTTTCCAGTCACCTCGTGTTCTGTTCAGCACTCAGCCCCCACCATTGTCAAGAGATGAGCAAAGTGTGGAGTTCCTGGACAATGAAGACACTCTTAACAGGAATATCCGGACAAACGTGAAACTCATCCTGTGCATAACCGAGGGA

>Bse_RL2_rep_c2194

ATGCATAGCGTAACGTCCATGTTGTGCTACACTCTGATTATAGCATTTCTGATTGGCATATGGGCAGCACCAAAATCTGAAGATAATGTCCCTCTGGACTCCCCTGCAACATCTGACCTTTCTGACACCAGCTGTGCTCAAACTCACGAGGGTCTGAAAACATCTCTAACTACAGATCAGCGCCATCCTGCTCCTAAGAAGGCAGAAGATCAAGAACTTGGATCAGCAGCCAACATCATTGTGGATCCCAAGCTTTTTCAGAAGAGGCGTTTCCAGTCACCTCGTGTTCTATTCAGCACTCAGCCCCCACCCTTGTCAAGAGATGAGCAAAGTGTGGAGTTCCTGGACAATGAAGACACTCTTAACAGGAATATCCGGGACCAAACG

>Bse_RL2_rep_c3514

ATGCATAGCGTAACCTCCATGTTGTGCTACACTCTGATTATAGCATTTCTGATTGGCATATGGGCAGCACCAAAATCTGAAGATAATGTCCCTCTGGGCTCCCCTGCAACATCTGACCTTTCTGACACCAGCTGTGCTCAAACTCACAAGGGTCTGAAAACATCTCTAAATACAGATCAGCGCCATCCTGCTCCTAAGAAGGCAGAGAATCAAGAACTTGGATCAGCAGCCAACATCATTGTGGATCCCAAGCTTTTC

>Bse_RL2_rep_c4864

ATGCATAGCGTAACATCCATGTTGTGCTACACTCTGATTATAGCATTTCTGATTGGCATATGGGCAGCACCAAAATCTGAAGATAATGTCCCTCTGGGCTCCCCTGCAACATCTGACCTTTCTGACACCAGCTGTGCTCAAACTCACAAGGGTCTGAAAACATCTCTAAATACAGATCAGCGCCATCCTGCTCCTAAGAAGGCAGAGAATCAAGAACTTGGATCAGCAGCCAACATCATTGTGGATCCCAAGCTTTTTCAGAAGAGGCGTTTCCAGTCACCTCGTGTTCTGTTCAGCACTCAGCCCCCACCATTGTCAAGAGATGAGCAAAGTGTGGAGTTCCTGGACAATGAAGACACTCTTAACAGGAATATCCGGACCAAACGTGAAACTCATCCTGTGCATAACCGAGGGGAATATTCTGTGTGTGACAGTATCAGTGTCTGGGTTGCCAACAAAACCAAAGCAATGGACATCAAAGGCAAACCGGTGACTGTGATGGCGGATGTAAATCTTAATAACCATGTCTACAAGCAGTACTTTTTTGAAACCAAGTGCAAAAATCCAAATCCAGTACCGAGTGGGTGCAGGGGCACC

>Bse_RL2_rep_c4921

ATGCATAGCGTAACGTCCATGTTGTGCTACACTCTGATTATAGCATTTCTGATTGGTATATGGGCAGCACCAAAATCTGAAGATAATGTCCCTCTGGGCTTCCCTGCAACATCTGACCTTTATGACACCAGCTGTGCTCAAACTCACAAGGGTCTGAAAACATCTCTAAATACAGATCAGCGCCATCCTGCTCCTAAGAAGGCAGAGGATCAAGAACTTGGATCAGCAGCCAACATCATTGTGGATCCCAAGCTTTTTCAGAAGAGGCGTTTCCAGTCACCTCGTGTTCTGTTCTGCACTCAGCCCCCACCGTTGTCAAGAGATGAGCAAAGTATGGAGCTCCCCGACAATGAAGATGCTCTTAACATGAGTATCTGGGCCCAACGTGAAAATCATCCTGCGTATAATGTAGGGGAACATTCTGTGTGTGACAGTATCAATAGCTGGGTTACCAAAACCACAGCAACGGACATCTACGACAATAGGGTGATTGTG

>Bse_RL2_rep_c895

ATGCATAGCGTAACATCCATGTTGTGCTACACTCTGATTATAGCATTTCTGATTGGCATATGGGCAGCACCAAAATCTGAAGATAATGTCCCTCTGGGCTCCCCTGCAACATCTGACCTTTCTGACACCAGCTGTGCTCAAACTCACAAGGGTCTGAAAACATCTCTAAATACAGATCAGCACCATCCTGCTCCTAAGAAGGCAGAGGATCAAGAACTTGGATCAGTAGCAAACATCATTGTGGATCCCAAGCTTTTTCAGAAGAGGCGTTTCCAGTCACCTCGTGTTCTGTTCAGCACTCAGCCCCCACCGTTGTCAAGAGATGAGCAAAGTGTGGAGTTCCCCGACAATGAAGACGCTCTTAACATGAGTATCCGGGCCAAACGTGAAAATCATCCTGCGTATAATGTAGGGGAACATTCTTTGTGTGACAGTATCAATAACTGGGTTACCAAAACCACAGCAACGGACATCCACGACAATAGGGTGACTGTGACGGCGGAGATAAATATTAATAACCAATTCTTCAAGCAGTACTTTTTTGAAACCAAGTGCAGAAATCCAAATCCATTACCGAGTGGGTGCAGAGGCATTGATTCCAGGTATTGGAATTCTTATTGCACCACAACACAGTCATTTGTCATGGCATTAACAACGGAAGACAATCGGGCATCCTGGCGCTTCATTCGGATTGACACTGCCTGTGTGTGCGTAATCAGTAGAAAATCTGACAGCTTC

>Bse_RL2_rep_c972

ATGCATAGCGTAACGTCCATGTTGTGCTACACTCTGATTATAGCATTTCTGATTGGCATATGGGCAGCACCAAAATCTGAAGATAATGTCCCTCTGGGCTCCCCTGCAACATCTGACCTTTCTGACACCAGCTGTGCTCAAACTCACAAGGGTCTGAAAACATCTCTAAATACAGATCAGCGCCATCCTGCTCCTAAGAAGGCAGAGGATCAAGAACTTGGATCAGCAGGCAACATCATTGTGGATCCCAAGCTTTTTCAGAAGAGGCGTTTCCAGTCACCTCGTGTTCTGTTCAGCACTCAGCCCCCACCGTTGTCAAGAGATGAGCAAAGTGTGGAGTTCCCCGACAATGAAGACGCTCTTAACATGAATATCCGGGCCAAACGTGAAAATCATCCTGCGTATAATGTAGGGGAACATTCTGTGTGTGACAGTATCAATAGCTGGGTTACCAAAACCACAGCAACGGACATCCACGACAATAGGGTGACTGTGACGGCGGAGATAAATCTTAATAACCATGTCTACAATCAGTACTTTTTTGAAACCAAGTGCATAAATCCAAATCCAGTACCGAGTGGGTGCAGGGGCATTGATTCCAGGTATTGGAATTCTTATTGCACCACAACACAGTCATTTGTCATGGCATTAACCATGGAAGGCAATCGGACATCCTGGCGCTTCATTCGGATTGACACTGCCTGTGTGTGCGTAATCAGTAGAAAATCTGACAGCTTC

>GC_RL8_rep_c1593

ATGTCCATGCTGTGCTACACTCTGATTATAGCATTTCTGATTGGCATATGGGCAGCACCAAAATCTGAAGATAATGTCCCTCTGGGCTCCCCTGTAACATCTGACCTTTCTGACACCAGCTGTGCTCAAACTCACAAGGGTCTGAAAACATCTCGAAATACAGATCAGCACCATCCTGCTCCTAAGAAGGCAGAGGATCAAGAACTTGGATCAGTAGCAAACATCATTGTGGATCCCAAGCTTTTTCAGAAGAGGCAGTTCCAGTCGTCTCGTGTTCTGTTCAGCACTCAGCCCCCACGT

>Dde_RL7_rep_c182

ATGATGTATAGCGTAATGTCCATGTTGTGCTACACTCTGATTATTGCATTTCTGATTGGCATATGGGCAGCACCAAAATCTGAAGATAATGTCCCTCTGGGCTCCCCTGCAACCAGCTGTGCTCAAACTCACGAGGGTCTGAAAACATCTCGAAATACAGATCAGCGCCCTCCTGCTCCTAAGAAGGCAGAGGATCAAGAACTTGGATCAGTAGCAAACATCATTGTGGATCCCAAGCTTTTTCAGAAGAGGCGTTTCCAGTCACCTCGTGTTCTGTTCAGCACTCAGCCCCCACCATTGTCAAGAGATGAGCAAAGTGTGGAGTTCCTCGACAATGAAGATGCTCTTAACAGGAATATCCGGGCCAAACGTGAAAATCATCCTGTGCATAACCTAGGGGAACATTCTGTGTGTGACAGTATCAGTGGCTGGGTTAACAAAACCACAGCAACGGACATGTACGGCAATATGGTGACTGTGATGGCGGAGATAAATCTTAATAATGAAGTCTATAAGCAGTACTTTTTTGAAACCAAGTGCAGAAATCCAAATCCAAATCCACTACTGAGTGAGTGCAGGGGCATTGATTCCAGGCTTTGGAATTCTTATTGCACCAGAACACAGACATTTGTCAGGGCATTAACCATGGCAGGCAATGTGACATCCTGGCGCTTCATTCGGATTGACACTGCCTGTGTGTGTGTAATCATTAGAAAAACTGACAACTTC

>Dde_RL7_rep_c307

ATGTATAGCGTAATGTCCATGTTGTGCTACACTCTGATTATTGCATTTCTGATTGGCATATGGGCAGCACCAAAATCTGAAGATAATGTCCCTCTGGGCTCCCCTGCAACCAGCTGTGCTCAAACTCACGAGGGTCTGAAAACATCTCGAAATACAGATCAGCGCCCTCCTGCTCCTAAGAAGGCAGAGGATCAAGAACTTGGATCAGTAGCAAACATCATTGTGGATCCCAAGCTTTTTCAGAAGAGGCGTTTCCAGTCACCTCGTGTTCTGTTCAGCACTCAGCCCCCACCATTGTCAAGAGATGAGCAAAGTGTGGAGTTCCTCGACAATGAAGATGCTCTTAACAGGAATATC

>Ecu_RL8_rep_c378

ATGATGCATAGCGTAATGTCCATGCTGTGCTACACTCTGATTATTGCATTTCTGATTGGCATATCGGCAGCACCAAAATCTGAAGATAATGTCCCTCTGGGCTCCCCTGCAACCAGCTTTGCTCAAACTCATGAGGGTCTGAAAACATCTCGAAATACAGATCAGCACCATCCTGCTCCTAAGAAGGCAGAGGATCAAGAACTTGGATCAGTAGCAAACATCATTGTGGATCCCAAGCTTTTTCAGAAGAGGCATTTCCAGTCACTTCGTGTTCTGTTCAGCACTCAGCCCCCACCATTGTCAAGAGATGAGCAAAGTGTGGAGTTCCTGGACAATGAAGATGCTCTTAACAGGAATATCCGGACCAAACGTGAAAATCATCCTGTGCATAACCTAGGGGAACATTCTGTGTGTGACAGTATCAGTGTCTGGGTTAGCAACAAAACCAACGCAACGGACATCAAAGGAAATATGGTGACTGTGATGGTAGACATAAATCTTAATAATGAAGTCTACAAGCAGTACTTTTTTGAAACCAAGTGCAGAAATCCAAATCCAGTACCGAGTGGGTGCAGGGGCACTGATTCCAGGCATTGGAATTCTTATTGCACCACAACACAGACATTTGTCAGGGCATTAACCATGGAAGGCAATCGGGCATCCTGGCGCTTCATTCGGATTGACACTGCCTGTGTGTGCGTAATCAGTAGAAAAACTGTCAACTTC

>Ecu_RL8_rep_c757

CTGATGCATAGCGTAATGTCCATGCTGTGCTACACTCTGATTATTGCATTTCTGATTGGCATATCGGCAGCACCAAAATCTGAAGATAATGTCCCTCTGGGCTCCCCTGCAACCAGCTTTGCTCAAACTCATGAGGGTCTGAAAACATCTCGAAATACAGATCAGCACCATCCTGCTCCTAAGAAGGCAGAGGATCAAGAACTTGGATCAGTAGCAAACATCATTGTGGATCCCAAGCTTTTTCAGAAGAGGCATTTCCAGTCACTTCGTGTTCTGTTCAGCACTCAGCCCCCACCATTGTCAAGAGATGAGCAAAGTGTGGAGTTCCTGGACAATGAAGATGCTCTTAACAGGAATATCCGGACCAAACGTGAAAATCATCCTGTGCATAACCTAGGGGAACATTCTGTGTGTGACAGTATCAGTGTCTGGGTTAGCAAC

>For_RL9_rep_c360

ATGTCCATGCTGTGCTACACTCTGATTATAGCATTTCTGATTGGCACATGGGCAGCACCAAAATCTGAAGATAATGTCCCTCTGGGCTCCCCTGCAACATCTGACCTTTCTGACACCGGCTGTGCTAAAACTCATGAGGGTCTGAAAACATCTCGAAATACAGATCAGCGCCATCCTGCTCCTAAGAAGGCAGAAGATCAAGAACTTGGATCAGCAGCCAACATCATTGTGGATCCCAAGCTTTTTCAGAAGTGGCGTTTCCAGTCACCTCGTGTTCTGTTCAGCACTCAGCCCCCACCATTGTCAAGAGATGAGCAAAGTGTGGAGTTCCTGGACAATGAAGACACTCTTAACAGGAATATCCGGACCAAACGTGAACATCATCCTGTGCATGACCAAGGAGAACATTCTGTGTGTGACAGTATCAGTGTCTGGGTTGCCAACAAAACTATAGCAACAGACATCAAAGGCAATGTGGTGACTGTGATAGTGGACATAAATTTTAATAATGAAGTCTACAAGCAGTACTTTTTTGAAACCAAGTGCAGAAATCCAAATCCAGTATCGAGTGGGTGCAGGGGCATTGATTCCAGGCATTGGAATTCTTATTGCACCACAAAACAAACATTTGTCAAGGCATTAACCATGGAAGACAATCTGACATTCTGGCGCTTCATTCGGATTGACACTGCCTGTGTGTGCGTAATCAGTAGAAAAGCTGACAACTTC

>For_RL9_rep_c523

ATGTCCATGCTGTGCTACACTCTGATTATAGCATTTCTGATTGGCACATGGGCAGCACCAAAATCTGAAGATAATGTCCCTCTGGGCTCCCCTGCAACATCTGACCTTTCTGACACCGGCTGTGCTAAAACTCATGAGGGTCTGAAAACATCTCGAAATACAGATCAGCGCCATCCTGCTCCTAAGAAGGCAGAAGATCAAGAACTTGGATCAGCAGCCAACATCATTGTGGATCCCAAGCTTTTTCAGAAGTGGCGTTTCCAGTCACCTCGTGTTCTGTTCAGCACTCAGCCCCCACCATTGTCAAGAGATGAGCAAAGTGTGGAGTTCCTGGACAATGAAGACACTCTTAACAGGAATATCCGGACCAAACGTGAACATCATCCTGTGCATGACCAAGGAGAACATTCTGTGTGTGACAGTATCAGTGTCTGGGTTGCCAACAAAACTATAGCAACAGACATCAAAGGCAATGTGGTGACTGTGATAGTGGACATAAATCTTCATAATGAAGTCTACAAGCAGTACTTTTTG

>Hbu_RL1_rep_c521

ATGATGCATAGCGTAATGTCCATGTTGTGCTACACTCTGATTATTGCATTTCTGATTGGCATATGGGCAGCACCAAAATCTGAGGATAATGTCCCTCTGGGCTCCCCTGCAACATCTGACCTTTCTGACACCAGCTGTGCTCAAATTCACGAGGGTCTGAAAACATCTCGAAATACAGATCAGCGCCATCCTGCTCCTAAGAAGGCAGAGGATCAAGAACTTGGATCAGCAGCCAACATCATTGTGGATCCCAAGCTTTTTCAGAAGAGGCGTTTCCAGTCACCCCGTGTTCTGTTCAGCACTCAGCCCCCACCGTTGTCAAGAGATGAGCAAAGTGTGGAGTTCCTCGACAATGAAGACGCTCTTAACAGGAATATCCGGGCCAAACGTGAAAATCATCCTGTGCATAACCAAGGGGAACATTCTGTGTGTGACAGTGTCAGTGACTGGGTTATCAAAACCACAGCAACGGACATCCACGGCAATATGGTGACTGTGATGGAGGACATAACTCTTAATAATGAGGTCTACAAGCAGTACTTTTTTGAAACCAAGTGCAGAAATCCAAATCCAAATCCACTACAGAGTGAGTGCAGGGGCATTGATTCCGGGCTTTGGAATTCTTATTGCACCAGAACACAGACATTTGTCAAGGCATTAACCATGGTAGGCAATCAGGCATCCTGGCGCTTCATTCGTATTGACACTGCCTGTGTGTGCGTAATCATTAGAAAAACTGACAACTTC

>Hbu_RL1_rep_c597

ATGATGCATAGTGTAATGTCCATGTTGTGTTACACTCTGATTATTGCATTTCTGATTGGCATATGGGCAGCACCAAAATCTGAGGATAATGTCCCTCTGGGCTCCCCTGCAACATCTGACCTTTCTGACACCAGCTGTGCTCAAACTCATGAGGGTCTGAAAACATCTCGAAATACAGATCAGCGCCATCCTGCTCCTAAGAAGGCAGAGGATCAAGAACTTGGATCAGTAGCAAACATCATTGTGGATCCCAAGCTTTTTCAGAAGAGGCGTTTCCAGTCACCTCGTGTTCTTTTCAGCACTCAGCCCCCACCGTTGTCAAGAGATGAGCAAAGTGTGGAGTTCCTCGACAATGAAGACGCTCTTAACAGGAATATCCGGGCCAAACGTGAAAATCATCCTGTGCATAACCAAGGGGAACATTCTGTGTGTGACAGTGTCAGTGATTGGGTTATCAAAACCACAGCAACGGACATCCACGGCAATATGGTGAATGTGATGGAGGACATAAATCTTAATAATGAGGTCTACAAGCAGTACTTTTTTGAAACCAAGTGCAGAAATCCAAATCCAAATCCACTACAGAGTGAGTGCAGGGGCATTGATTCCAGGCTTTGGAATTCTTATTGCACCAGAACACAGACATTTGTCAAGGCATTAACCATGGCAGGCAATCAGGCATCCTGGCGCTTCATTCGTATTGACACTGCCTGTGTGTGCGTAATCATTAGAAAAACTGACAACTTC

>Hbu_RL1_rep_c786

GGTCCCCCTGCAACATCTGACCTTTCTGACACCAGCTATGCTAAAACTCATGAAGCTCTGAAAACATCTCGAAACACAGATCAGCACTATCCTGCTCCTAAAAAGGCAGAGGATCAAGAATTTGGGTCAGCAGCAAATATCATTGTGGATCCAAAGCTTTTTCAGAAGAGGCGGTTCCAGTCGCCTCGTGTTTTGTTCAGCACTCAGCCCCCACCATTGTCAAGAGATGAGCAAAGTGTGGACAATGCAAACTCTCTTAATAGGAATATCCGGGCCAAACGTGAAGATCATCCTGTGCATAAACGAGGGGAATATTCTGTGTGTGACAGTGTCGATGTCTGGGTTGCCAACAAAACCACAGCAACGGACATCAGAGGCAATCTGGTGACTGTGATGGTGGATGTAAACATTAATAACAATGTCTACAAGCAGTTACTTTTTGAGACCAAGTGCAGAAATCCAAACCCAGTACCAACTGGGTGCAGGGGCATTGATGCCAGGCATTGGAATTCGTATTGCACCACAACAAACACATTTGTCAAGGCATTAACCATGGAAGGCAATCAGGCATCCTGGCGCTTCATTCGGATTGACAGTGCCTGTGTGTGTGTAATCAGTAGAAAA

>Hbu_RL1_rep_c923

ATGTTGTGCTACACTCTGATTATTGCATTTCTGATTGGCATATGGGCAGCACCAAAATCTGAGGATAATGTCCCTCTGGGCTCCCCTGCAACATCTGACCTTTCTGACACCAGCTGTGCTCAAACTCATGAGGGTCTGAAAACATCTCGAAATACAGATCAGCGCCATCCTGCTCCTAAGAAGGCAGAGGATCAAGAACTTGGATCAGCAGCCAACATCATTGTGAATCCCAAGCTTTTTCAGAAGAGGCGTTTCCAGTCACCTCGTGTTCTGTTCAGTACTCAGCCCCCACCGTTGTCAAGAGATGAGCAAAGTGTGGAGTTCCTGGACAATGAAGACGCTCTTAACAGGAATATCCGGGCCAAACGTGAAAATCATCCTGTGCATAACAGAGGGGAACATTCTGTGTGTGACAGTATCAGTGTCTGGGTTACCAACAAAACCAAAGCAACGGACATCAAAGGCAATATGGTGACTGTGATGGTGGATGTAAATCTTAATAATGAAGTCTACAAGCAGTACTTTTTTGAAACCAAGTGCAGAAATCCAAATCCAGTACCGAGTGGGTGCAGGGGCACTGATTCCAGGCATTGGAATTCTTATTGCACCACAACACAGACATTTGTCAGGGCATTAACCATGGAAGGCAATCGGGCATCCTGGCGCTTCATTCGGATTGACACTGCCTGTGTGTGCGTAATAATTAGAAAAACTGACAACTTC

>Hbu_RL1_rep_c961

ATGGTGCATAGCGTAATGTCCATGTTGTGCTACACTCTGATTATTGCATTTCTGATTGGCATATGGGCAGCACCAAAATCTGAAGATAATGTCCCTCTGGGCTCCCCTGCAACATCTGACCTTTCTGACACCAGCTGTGCTCAAACTCATGAGGGTCTGAAAACATCTCGAAATACAGATCAGCACCATCCTGCTCCTAAGAAGGCAGAGGATCAAGAACTTGGATCAGCAGCCAACATCATTGTGGATCCCAAGCTTTTTCAGAAGAGGCGTTTCCAGTCACCTCGTGTTCTGTTCAGTACTCAGCCCCCACCGTTGTCAAGAGATGAGCAAAGTGTGGAGTTCCTGGACAATGAAGACGCTCTTAACAGGAATATCCGGGCCAAACGTGAAAATCATCCTGTGCATAACAGAGGGGAACATTCTGTGTGTGACAGTATCAGTGTCTGGGTTACCAACAAAACCAAAGCAACGGACATCAAAGGCAATATGGTGACTGTGATGGTGGATGTAAATCTTAATAATGAAGTCTACAAGCAGTACTTTTTTGAAACCAAGTGCAGAAATCCAAATCCAGTACCGAGTGGGTGCAGGGGCACTGATTCCAGGCATTGGAATTCTTATTGCACCACAACACAGACATTTGTCAGGGCATTAACCATGGAAGGCAATCGGGCATCCTGGCGCTTCATTCGGATTGACACTGCCTGTGTGTGCGTAATCATTAGAAAAACTGACAACTTC

>Pmo_RL10_rep_c337

ATGTCCATGTTGTGCTACACTCTGATTATAGCATTTCTGATTGGCATATGGGCAGCACCAAAATCTGAAGATAATGTCCCTCTGGGCTCCCCTGCAACATCTGACCTTTCTGACACCAGCTGTGCTCAAACTCACAAGGCTCTGAAAACATCTCGAAATACAGATCAGCGCCATCCTGCTCCTAAGAAGGCAGAAGATCAAGAACTTGGATCAGCAGCCAACATCATTGTGGATCCCAAGCTTTTTCAGAAGAGGCGTTTCCAGTCACCTCGTGTTCTGTTCAGCACTCAGCCCCCACCATTGTCAAGAGATGAGCAAAGTGTGGAGTTCCTGGACAATGAAGACGCTCTTAACAGGAATTTCCGGGCCAAACGTGAAACTCATCCTGTGCATAACCAAGGGGAATATTCTGTGTGTGACAGTATCAGTGTCTGGGTTGGCAACAAAACCAAAGCAATAGACATCAAAGACAAACCGGTGACTGTGATGGTCGATGTAAATCTTAATAACCATGTCTTCAAGCAGTACTTTTTTGAAACCAAGTGCAGAAATCCAAATCCAGTACCAAGTGGGTGCAGGGGCATTGATTCCAGGCATTGGAATTCTTATTGCACCACAACACAGACATATGTCAGGGCATTAACCATGGAAGGCAATCAGGCATCCTGGCGCTTCATTCGGATTGACGCTGCCTGTGTGTGCGTAATCAGTAGAAAA

>Van_RL12_rep_c317

ATGCATAGCGTAATGTCCATGTTGTGCTACACTCTGATTATTGCATTTCTGATTGGCATATGGGCAGCACCAAAATCTGAAGATAATGTCCCTCTGGGCTCCCCTGCAACATCTGACCTTTCTGACACCAGCTGTGCTCAAACTCACGAGGGTCTGAAAACATCTCGAAATACAGATCAGCGCCATCCTGCTCCTAAGAAGGCAGAGGATCAAGAACTTGGATCAGCAGCCAACATCATTGTGGATCCCAAGCTTTTTCAGAAGAGGCGTTTCCAGTCACCTCGTGTTCTGTTCAGCACTCAGCCCCCACCATTGTCAAGAGATGAGCAAAGTGTGGAGTTCCTAGACAATGAAGACACTCTTAACAGGAATATCTGGACCAAACGTGAAACTCATCCTGTGCATAACCAAGGGGAATATTCTGTGTGTGACAGTATCAGTGTCTGGGTTGCGGACAAAACCACAGCAACGGACATCAAAGGCAATACGGTGACTGTGATGACGGACATAAATATTAATAATGAAGTCTACAAGCAGTACTTT

>Van_RL12_rep_c673

CTGAAAACATCTCGAAATACAGATCAGCGCCATCCTGCTCCTAAGAAGGCAGAGGATCAAGAACTTGGATCAGCAGCCAACATCATTGTGGATCCCAAGCTTTTTCAGAAGAGGCGTTTCCAGTCACCTCGTGTTCTGTTCAGCACTCAGCCCCCACCATTGTCAAGAGATGAGCAAAGTGTGGAGTTCCTGGACAATGAAGACACTCTTAACAGGAATATCCGGACCAAACGTGAAACTCATCCTGTGCATAACCAAGGGGAATATTCTGTGTGTGACAGTATCAGTATCTGGGTTGCCGACAAAACCACAGCAACGGACATCAAAGGCAATACGGTGACTGTGATGACGGACATAAATATTAATAATGAAGTCTACAAGCAGTACTTTTTTGAAACCAAGTGCAGAAATCCAAATCCAGTACCGAGTGGGTGCAGGGGCATTGATTCCAGGCATTGGAATTCTTATTGCAGCACAACAGACACATTTGTCAAGGCATTAACCATGGAAGGCAATCGGGCATCCTGGCGCTTCCAAATTAAA

>Afe_RL4_rep_c388

GACAATGCAGACTCTCTTAATAGGAATATCCGGGCCAAACGTGGAACTCATCCTGTGCATAACCAAGGGGAATATTCTGTGTGTGATAGTGTCAGTGTCTGGGTTGCCAACAAAACCACAGCAACGGACATCAGAGGCAATCTGGTGACTGTGATGGTGGATATAAACCTTAATAACAATGTCTACAAGCAGTACTTTTTTGAGACCAAGTGCAGAAATCCAAACCCAGTACCAAGTGGGTGCAGGGGCATTGATGCCAGGCATTGGAATTCGTATTGCACCACAACACACACATTTGTCAGGGCATTAACCAAGGAAGGCAATCAGGCATCCTGGCGCTTCATTCGGATTGACACTGCCTGTGTGTGTGTAATCAGTAGAATAACTGAGAACTTTGGA

>CohCI9_RL3_c37

ATGTCCATGCTGTGCTACACTCTGATTATAGCATTTCTGATTGGCATATGGGCAGCACCAAAATCTGAAGATAATGTCCCTCTGGGGTCCCCTGCAACATCTGACCTTTCTGACACCAGCTATGCTAAAACTCATGAAGCTCTGAAAACATCTCGAAACACAGATCAGCACTATCCTGCTCCTAAAAAGGCAGAGGATCAAGAATTTGGGTCAGCAGCAAATATCATTGTGGATCCAAAGCTTTTTCAGAAGAGGCGGTTCCAGTCGCCTCGTGTTTTGTTCAGCACTCAGCCCCCACCATTGTCAAGAGATGAGCAAAGTGTGGACAATGCAAACTCTCTTAATAGGAATATCCGGGCCAAACGTGAAGATCATCCTGTGCATAAACGAGGGGAATATTCTGTGTGTGACAGTGTCAATGTCTGGGTTGCCAACAAAACCACAGCAACGGACATCAGAGGCAATCTGGTGACTGTGATGGTGGATGTAAACATTAATAACAATGTCTACAAGCAGTACTTTTTTGAGACCAAGTGCAGAAATCCAAACCCAGTACCAACTGGGTGCAGGGGCATTGATGCCAGGCATTGGAATTCGTATTGCACCACAACAAACACATTTGTCAAGGCATTAACCATGGAAGGCAATCAGGCATCCTGGCGCTTCATTCGGATTGACAGTGCCTGTGTGTGTGTAATCAGTAGAAAAAATGAGAACTTTGGA

>CohID2_RL5_rep_c250

ATGGGCAGCACCAAAAATCTGAAGATAATGTCCCTCTGGGGTCCCCCTGCAACATCTGACCTTTCTGACACCAGCTATGCTAAAACTCATGAAGCTCTGAAAACATCTCGAAACACAGATCAGCACTATCCTGCTCCTAAAAAGGCAGAGGATCAAGAATTTGGGTCAGCAGCAAATATCATTGTGGATCCAAAGCTTTTTCAGAAGAGGCGGTTCCAGTCGCCTCGTGTTTTGTTCAGCACTCAGCCCCCACCATTGTCAAGAGATGAGCAAAGTGTGGACAATGCAAACTCTCTTAATAGGAATATCCGGGCCAAACGTGAAGATCATCCTGTGCATAAACGAGGGGAATATTCTGTGTGTGACAGTGTCGATGTCTGGGTTGCCAACAAAACCACAGCAACGGACATCAGAGGCAATCTGGTGACTGTGATGGTGGATGTAAACATTAATAACAATGTCTACAAGCAGTTACTTTTTGAGACCAAGTGCAGAAATCCAAACCCAGTACCAACTGGGTGCAGGGGCATTGATGCCAGGCATTGGAATTCGTATTGCACCACAACAAACACATTTGTCAAGGCATTAACCATGGAAGGCAATCAGGCATCCTGGCGCTTCATTCGGATTGACAGTGCCTGTGTGTGTGTAATCAGTAGAAAA

>CohLL5_RL4_rep_c451

ATGTCCATGCTGTGCTACACTCTGATTATAGCATTTCTGATTGGCATATGGGCAGCACCAAAATCTGAAGATAATGTCCCTCTGGGGTCCCCTGCAACATCTGACCTTTCTGACACCAGCTATGCTAAAACTCATGAAGCTCTGAAAACATCTCGAAACACAGATCAGCACTATCCTGCTCCTAAAAAGGCAGAGGATCAAGAATTTGGGTCAGCAGCAAATATCATTGTGGATCCAAAGCTTTTTCAGAAGAGGCGGTTCCAGTCGCCTCGTGTTTTGTTCAGCACTCAGCCCCCACCATTGTCAAGAGATGAGCAAAGTGTGGACAATGCAAACTCTCTTAATAGGAATATCCGGGCCAAACGTGAAGATCATCCTGTGCATAAACGAGGGGAATATTCTGTGTGTGACAGTGTCGATGTCTGGGTTGCCAACAAAACCACAGCAACGGACATCAGAGGCAATCTGGTGACTGTGATGGTGGATGTAAACATTAATAACAATGTCTACAAGCAGTACTTTTTTGAGACCAAGTGCAGAAATCCAAACCCAGTACCAACTGGGTGCAGGGGCATTGATGCCAGGCATTGGAATTCGTATTGCACCACAACAAACACATTTGTCAAGGCATTAACCATGGAAGGCAATCAGGCATCCTGGCGCTTCATTCGGATTGACAGTGCCTGTGTGTGTGTAATCAGTAGAAAAAATGAGAACTTTGGA

>CohLL5_RL4_rep_c668

ATGTCCATGCTGTGCTACACTCTGATTATAGCATTTCTGATTGGCATATGGGCAGCACCAAAATCTGAAGATAATGTCCCTCTGGGGTCCCCTGCAACATCTGACCTTTCTGACACCAGCTATGCTAAAACTCATGAAGCTCTGAAAACATCTCGAAACACAGATCAGCACTATCCTGCTCCTAAAAAGGCAGAGGATCAAGAATTTGGGTCAGCAGCAAATATCATTGTGGATCCAAAGCTTTTTCAGAAGAGGCGGTTCCAGTCGCCTCGTGTTTTGTTCAGCACTCAGCCCCCACCATTGTCAAGAGATGAGCAAAGTGTGGACAATGCAAACTCTCTTAATAGGAATATCCGGGCCAAACGTGAAGATCATCCTGTGCATAAACGAGGGGAATATTCTGTGTGTGACAGTGTCAATGTCTGGGTTGCCAACAAAACCACAGCAACGGACATCAGAGGCAATCTGGTGACTGTGATGGTGGATGTAAACATTAATAACAATGTCTACAAGCAGTACTTTTTTGAGACCAAGTGCAGAAATCCAAACCCAGTACCAACTGGGTGCAGGGGCATTGATGCCAGGCATTGGAATTCGTATTGCACCACAACAAACACATTTGTCAAGGCATTAACCATGGAAGGCAATCAGGCATCCTGGCGCTTCATTCGGATTGACAGTGCCTGTGTGTGTGTAATCAGTAGAAAAAATGAGAACTTTGGA

>CohPH1_RL6_rep_c413

ATGTCCATGCTGTGCTACACTCTGATTATAGCATTTCTGATTGGCATATGGGCAGCACCAAAATCTGAAGATAATGTCCCTCTGGGGTCCCCTGCAACATCTGACCTTTCTGACACCAGCTATGCTAAAACTCATGAAGCTCTGAAAACATCTCGAAACACAGATCAGCACTATCCTGCTCCTAAAAAGGCAGAGGATCAAGAATTTGGGTCAGCAGCAAATATCATTGTGGATCCAAAGCTTTTTCAGAAGAGGCGGTTCCAGTCGCCTCGTGTTTTGTTCAGCACTCAGCCCCCACCATTGTCAAGAGATGAGCAAAGTGTGGACAATGCAAACTCTCTTAATAGGAATATCCGGGCCAAACGTGAAGATCATCCTGTGCATAAACGAGGGGAATATTCTGTGTGTGACAGTGTCAATGTCTGGGTTGCCAACAAAACCACAGCAACGGACATCAGAGGCAATCTGGTGACTGTGATGGTGGATGTAAACATTAATAACAATGTCTACAAGCAGTACTTT

>Abro_RL8_rep_c474

ATGTCTGGGAGGGAAAAGCACAATTTTGCCTGGAAAAGCACCTTTTTCAAGGTGCATAGCGTAATGTCCATGCTGTGCTACACTCTGATTATAGCGTTTCTGGTCGGCATATGGGCAGCACCAAAATGCGAAGATAATGCACCACTGGGGTCTCCTGCAACATCTGACATTTCTGAAAGCAGCCGGTCTAAGACACACCATGTTATGAAAACGTCTCGGCACAGAGGCCAGAACCAGCCTGTCACTGGGAAGGTAGAGGACAGAAAAACTGGGCAAGCTGCAAACATTATTGTGGATCCAAAGCTTTTTCAGAAGAGGCGGTTCCAGTCACCTCGGGTTTTGTTCAGCACACAGCCTCCACCTTTGTCAAGGGACGGGCAGAATGTGGAGTTCCTAGACAGTACAGACTCTCTCAATAGGAATATCCGTGCCAAGCGTGCAACTCATCCTGTGCATAACCGGGGAGAGTACTCCGTATGCGACAGTGTTAGTGTATGGGTAGCCAACAAAACCACGGCAACAGACATCAAAGGCAAAGAGGTGACTGTGCTGGTGGATGTAAACTTTAACAACAATGCTTACAAGCAATACTTTTTTGAGACCAAGTGCAGAGACCCTAAGCCAGTGTCCAGCGGGTGCCGAGGCATTGATGCCAGGCATTGGAATTCCTACTGCACCACTACGCACACCTTTGTCAAGGCACTGACCACGGAAGGCAAGCAGGCAGCCTGGCGCTTCATTCGGATTGACACTGCCTGTGTGTGCGTAATCAGTAGGAAAACTGAGAACCTC

>Abro_RL8_rep_c785

CCTCGGGTTTTGTTCAGCACACAGCCTCCACCTTTGTCAAGGGACGGGCAGAATGTGGAGTTCCTAGACAGTACAGACTCTCTCAATAGGAATATCCGTGCCAAGCGTGCAACTCATCCTGTGCATAACCGGGGAGAGTACTCCGTATGCGACAGTGTTAGTGTATGGGTAGCCAACAAAACCACGGCAACAGACATCAAAGGCAAAGAGGTGACTGTGCTGGTGGATGTAAACTTTAACAACAATGCTTACAAGCAATACTTTTTTGAGACCAAGTGCAGAGACCCTAAGCCAGTGTCCAGCGGGTGCCGAGGCATTGATGCCAGGCATTGGAATTCCTACTGCACCACTACGCACACCTTTGTCAAGGCACTGACCACGGAAGGCAAGCAGGCAGCCTGGCGCTTCATTCGGATTGACACTGCCTGTGTGTGCGTAATCAGTAGGAAAACTGAGAACCTC

>Hsu_RL11_rep_c662

ATGTCTGGAAGAGAAAAGCACAATTTTGCTGGGAAAAGCACTTTTTTCAAGGTGCATAGCGTAATGTCCATGCTGTGCTACACTCTGATTATAGCGTTTCTGATCGGCACATGGGCAGCACCAAAATGCGAAGATAATGCACCACTGGGGTCTCCTGCAACATCTGACATTTCTGAAAGAAGCCAGACTAAAACACGTCATGTTATGAAAACATCTCTGCACCGAGGCCAGAACCAGCCTGTTACTGGGAGGGTCAAGGAGAGAGAAACTGGGCAAGCTGCAAACATCATTGTGGATCCAAAGCTTTTTCAGAAGAGGCGGTTCCAGTCACCTCGGGTTTTGTTCAGCACACAGCCCCCACCTTTGTCAAGGGATGGGCAGAATGTGGAGTTCCTAGACAGTACAGACTCTCTCAGTAGGAATATCCGTGCTAAGCGTGCAACTCATCCTGTGCATAACCAGGGAGAGTACTCTGTATGTGACAGTGTTAGTGTGTGGGTTGCCAACAAAACCACAGCGACAGACATCAAAGGCAAAGAGGTGGATGTGATGGTGGATGTAAACATTAACAATAATGCTTACAAGCAGTACTTTTTTGAGACCAAGTGCAGAGACCCTAAGCCAGTGCCCAGTGGGTGCAGAGGCATTGATGCCAGGCATTGGAATTCCTACTGCACCACTACACACACCTTTGTCAGAGCACTGACCATGGAAGGCAAGCAGGCAGCCTGGCGTTTCATTCGGATTGACGCTGCCTGTGTATGCGTCATCAGTAGGAAAACCGAGAACCTC
